# Structural mechanisms underlying resistance to Switch-II pocket inhibitors in KRAS

**DOI:** 10.1038/s41467-026-78133-4

**Published:** 2026-09-29

**Authors:** Matthew J. Whitley, Marcin Dyba, Mayukh Chakrabarti, Brian P. Smith, Albert H. Chan, John-Paul Denson, Simon A. Messing, Ken Lin, Gabriel Cornilescu, Trent E. Balius, Dwight V. Nissley, Frank McCormick, Anna E. Maciag, Dhirendra K. Simanshu

**Affiliations:** 1 NCI RAS Initiative, Cancer Research Technology Program, Frederick National Laboratory for Cancer Research, Frederick, MD USA; 2 BridgeBio Oncology Therapeutics, South San Francisco, CA USA; 3 Helen Diller Family Comprehensive Cancer Center, University of California, San Francisco, CA USA

**Keywords:** X-ray crystallography, Molecular biophysics, Cancer therapeutic resistance, Drug discovery

## Abstract

Direct targeting of KRAS through the Switch-II pocket is an effective therapeutic strategy, but acquired resistance limits clinical benefit. We examine clinically observed resistance mutations (R68S, H95Q, Y96C, Y96D and Q99L) arising during KRAS G12C inhibitor therapy using biochemical, structural, computational and cellular approaches. Covalent and noncovalent binding measurements reveal mutation- and inhibitor-specific reductions in drug engagement across inhibitor classes. Structural analyses identify two principal resistance mechanisms: Type I, involving loss of key inhibitor contacts, and Type II, involving stabilization of pocket-occluding Switch-II conformations. Y96D exhibits both mechanisms through nucleotide-dependent non-native intramolecular interactions, whereas R68S primarily exhibits Type I resistance with modest Type II effects on pocket architecture and Switch-region dynamics. Y96C, H95Q and Q99L predominantly exhibit Type I resistance. All mutants retain RAF1 binding, whereas PI3Kα binding is selectively reduced by R68S and Q99L. Together, these findings provide structural insights for understanding on-target resistance to Switch-II pocket inhibitors.

## Abstract (web summary)

This study explores how clinically observed KRAS resistance mutations alter drug-binding interactions and dynamics of the Switch-II pocket while preserving oncogenic signaling, providing a structural framework for developing more durable KRAS inhibitors.

## Introduction

RAS proteins are small GTPases that function as molecular switches, cycling between inactive GDP- and active GTP-bound states to control downstream effector signaling. Among the three canonical RAS isoforms, KRAS is the most frequently mutated in human cancers, particularly in lung, colorectal, and pancreatic tumors, and is a central oncogenic driver. Targeting the KRAS G12C allele enabled the development of the first clinically effective direct RAS inhibitors by exploiting the nucleophilic mutant cysteine near the Switch-II pocket for covalent modification^1^, leading to the regulatory approval of sotorasib and adagrasib. These inhibitors bind to an inducible allosteric pocket in and around Switch-II, selectively engage the inactive, GDP-bound state, stabilize an inactive conformation of KRAS G12C, and suppress downstream MAPK signaling, resulting in beneficial clinical responses in subsets of patients. In practice, however, responses are often incomplete and transient. Resistance emerges through a combination of on-target and off-target mechanisms, including reactivation of adaptive pathways and acquired alterations that restore signaling despite continued drug exposure. On-target resistance includes secondary mutations in KRAS that interfere with inhibitor binding or alter nucleotide-state preferences^2–5^, whereas off-target resistance arises from rewiring of upstream and parallel pathways, activation of alternative RAS isoforms, and feedback reactivation of downstream effectors^5–8^.

KRAS inhibitors currently in clinical and advanced preclinical development fall into two broad mechanistic categories. One class comprises direct inhibitors that bind within the Switch-II pocket, including covalent and noncovalent agents that engage the GDP-bound state, as well as more recent compounds that bind both GDP- and GTP-bound KRAS. These span several allele-selective and emerging pan-KRAS or pan-RAS chemotypes^9–20^. A second class comprises indirect inhibitors that act by forming a ternary complex between KRAS and cyclophilin A, thereby blocking effector binding through a composite interface rather than by directly occupying the Switch-II pocket^21^. These distinct strategies impose different structural constraints on KRAS and are associated with overlapping but nonidentical resistance profiles^22^.

For direct Switch-II pocket inhibitors, analyses of tumors and experimental models with acquired resistance to KRAS G12C therapy have identified secondary substitutions that cluster around the Switch-II pocket, particularly at the Switch-II/helix α3 interface^2,4,5,23,24^. Recurrent clinically acquired mutations at residues R68, H95, Y96, and Q99 have been observed across independent clinical cohorts treated with different KRAS G12C inhibitors. Precise frequencies of individual mutations are difficult to estimate, as available clinical datasets are small in both number and sample size and often focus on identifying mutants and mechanisms rather than establishing mutational prevalence. In a 143-patient post-progression cohort in which Switch-II pocket mutations emerged in 14% of patients overall, Y96D was the most frequently observed Switch-II pocket substitution^5^. Additional recurrent mutations, including R68S, H95Q, Y96C, and Q99L, have also been reported in patients or preclinical resistance models^2,4^. Furthermore, resistance-associated substitutions have also been reported at positions outside the Switch-II pocket, including sites within Switch-I, the P-loop, and more distal regions that influence global KRAS conformational dynamics and nucleotide-state occupancy^2,5,23,24^. Structural and computational studies indicate that the Switch-II pocket residues help define the pocket’s geometry, physicochemical environment, and conformation. Additionally, they define its flexibility and contribute to inhibitor recognition through a combination of direct contacts and stabilization of pocket architecture. Because individual inhibitors adopt distinct binding conformations within this shared pocket, substitutions at these sites, whether within the Switch-II pocket itself or at distal positions that indirectly reshape it, are expected to confer mutation- and inhibitor-specific resistance rather than uniformly disrupting all direct KRAS inhibitors.

Beyond effects on inhibitor binding, the functional consequences of Switch-II pocket resistance mutations for effector engagement and signaling output remain less well defined. Although these substitutions emerge under therapeutic pressure, they must retain sufficient effector interactions to preserve oncogenic fitness. Many Switch-II pocket residues are spatially separated from the core RAF1 binding interface but lie closer to regions implicated in PI3Kα recognition, raising the possibility that resistance mutations may differentially modulate downstream pathways rather than leaving effector interactions unaffected. However, how individual resistance mutations balance inhibitor escape with preservation of signaling competence has not been sufficiently systematically examined.

In this work, we use an integrated combination of biochemical and biophysical measurements, structural analysis, molecular simulations, and cellular signaling assays to examine a panel of resistance mutations representing the major recurrent Switch-II pocket hotspots that are relevant to the growing number of clinically approved and emerging Switch-II pocket inhibitors. We characterize mutation-dependent effects on covalent engagement by representative KRAS G12C inhibitors, assess how resistance substitutions alter non-covalent binding in the Switch-II pocket, and determine structures of five recurrent Switch-II pocket resistance mutants to define the structural basis of resistance. We further examine how individual mutations influence RAF1 and PI3Kα effector binding and evaluate MAPK pathway suppression across inhibitors in a mutation-specific manner in cells. Together, these results link mutation-specific pocket remodeling to inhibitor sensitivity, effector engagement, and signaling output, providing a mechanistic understanding on-target resistance to direct KRAS inhibitors and informing the design of next-generation agents with improved response to clinically relevant resistance mutations.

## Results

### Resistance mutations in the Switch-II pocket and their structural context across covalent KRAS G12C inhibitors

**Fig. 1:**
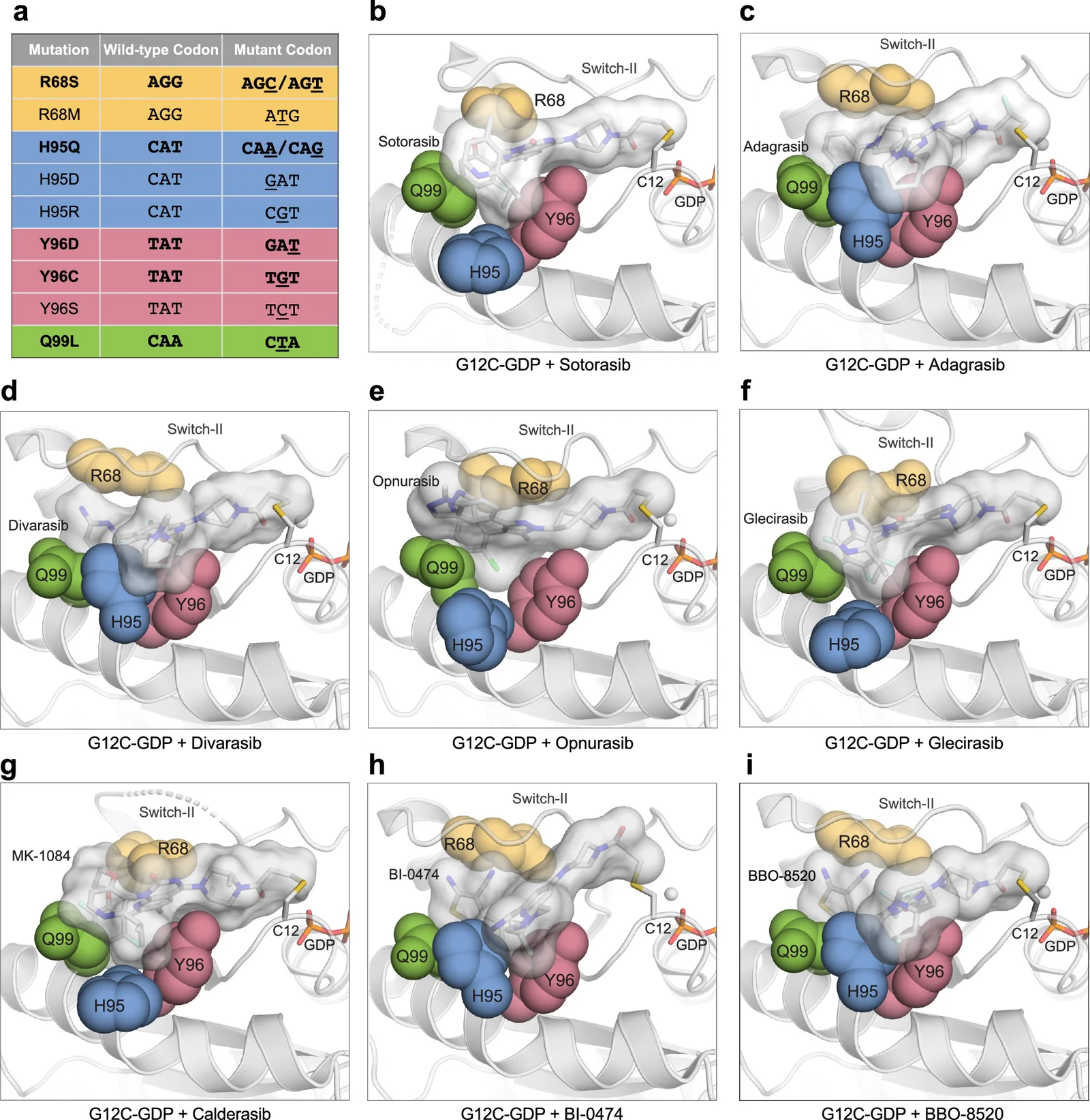
Acquired resistance mutations in KRAS targeted by Switch-II pocket inhibitors and their structural context across covalent KRAS G12C inhibitors. **a** Overview of selected acquired resistance mutations associated with direct KRAS Switch-II pocket inhibitors that localize to the Switch-II/helix α3 region, together with the corresponding wild-type and mutant codons; the specific codon changes are underlined. The five mutations analyzed in the present study, spanning four distinct sites, are highlighted in bold. **b–i** Structures of KRAS G12C bound to GDP and covalently modified by a representative subset of G12C inhibitors selected from the broader set reported to date. The selected inhibitors are sotorasib (PDB 6OIM), adagrasib (PDB 6UT0), divarasib (PDB 9DMM), opnurasib (PDB 7R0M), glecirasib (PDB 9KPN), calderasib (PDB 8S8C), BI-0474 (PDB 8AFB), and BBO-8520 (PDB 8V3A). Side chains at positions R68, H95, Y96, and Q99 are shown as spheres and color-coded to match (**a**), while the covalently bound inhibitors are depicted as gray sticks beneath a transparent gray surface. These structures illustrate that different G12C inhibitors engage the Switch-II pocket and resistance sites in distinct ways. Notably, BBO-8520 targets both the GDP- and GTP-bound states of KRAS G12C (only the GDP-bound structure is shown), whereas the remaining inhibitors selectively bind the inactive GDP-bound state.

Analysis of clinically acquired resistance to covalent KRAS G12C inhibitors has repeatedly identified secondary mutations that cluster in and around the Switch-II pocket, particularly at the Switch-II/helix α3 interface^2,4,5,23,24^. This region constitutes the principal binding site for direct KRAS G12C inhibitors and is formed in part by residues R68, H95, Y96, and Q99, which directly shape pocket geometry and contribute key inhibitor contacts. Figure 1a summarizes representative clinically relevant mutations at these four sites: R68S/M, H95Q/D/R, Y96D/C/S, and Q99L, together with the corresponding wild-type and mutant codons. For further analysis, we selected five mutations: R68S, H95Q, Y96C, Y96D, and Q99L, as they represent the most recurrent clinically acquired Switch-II pocket resistance mutations, including two chemically distinct substitutions at Y96.

To place these resistance mutations in a structural context, we compared crystal structures of KRAS G12C covalently modified by a representative set of clinical Switch-II pocket inhibitors (Fig. 1b–i). This set includes the FDA-approved inhibitors sotorasib (AMG510) and adagrasib (MRTX849), as well as late-stage or clinical candidates divarasib (GDC-6036), opnurasib (JDQ-443), glecirasib (JAB-21822), calderasib (MK-1084), BI-0474, and the dual GDP/GTP inhibitor BBO-8520^9,11,13–18^. With the exception of BBO-8520, all inhibitors engage KRAS G12C exclusively in the inactive, GDP-bound state via covalent modification of C12.

Despite targeting a common Switch-II pocket, these inhibitors adopt distinct binding poses that reflect differences in the core scaffold and substituent chemistries, resulting in differential interactions with the resistance residues R68, H95, Y96, and Q99. The G12C covalent compounds generally contain a core that is sandwiched between Y96 and Switch-II, a single ring or a bi-cycle that interacts with the C-terminal parts of Switch-II and helix α3, including R68 and Q99, another single ring or bi-cycle that points to the solvent and often interacts with H95 and/or Q99, and an acrylamide warhead that points toward the nucleotide and C12. The chemical structures of these inhibitors, with atoms contacting R68, H95, Y96, and Q99 highlighted, are shown in Supplementary Fig. 1. Sotorasib has almost no contacts with H95 and induces a distinct conformation of R68 that allows it to form a hydrogen bond with its phenol -OH. Adagrasib, on the other hand, has more contacts with H95 and fewer with Q99, because its solvent-directing substituent is located at a different position on the bicyclic core. Importantly, this rearrangement allows the core to form a hydrogen bond with H95, a strategy that is observed again in divarasib and BBO-8520. Divarasib is similar to adagrasib, but with Cl and F added to the core, resulting in more contacts with R68 and H95. Opnurasib has the smallest core and consequently makes the least contact with Y96, and it projects its solvent-facing indazole away from H95. Glecirasib is a close analog of sotorasib, but it lacks the -OH on the benzene ring to make a hydrogen bond with R68. Calderasib is also a sotorasib analog, but it bridges the benzene and pyridine with a linker that forms a macrocycle. It does not form a hydrogen bond with R68, but the linker improves its contacts with Q99 compared to sotorasib. BI-0474 has a very different Y96-contacting core, in which the two rings are separated by a bond instead of being fused together. Like adagrasib, it forms a hydrogen bond with H95 but using its oxadiazole nitrogen. Finally, the dual inhibitor BBO-8520 resembles adagrasib and divarasib, but with more contacts with R68, including a water-mediated interaction with the cyano group. The additional methyl on the piperazine also allows BBO-8520 to maintain van der Waals contacts with the active form of KRAS-G12C when the warhead is shifted to avoid steric clashes with the γ-phosphate of GTP.

Overall, the analysis of these structures demonstrates that although all direct covalent KRAS G12C inhibitors bind within the Switch-II pocket, they exploit this site in inhibitor-specific ways dictated by scaffold chemistry and binding geometry. Consequently, resistance mutations at common residues are poised to differentially disrupt inhibitor engagement, providing a structural framework for understanding both shared and drug-specific resistance mechanisms.

### Resistance mutations differentially alter covalent engagement kinetics of KRAS G12C inhibitors

**Fig. 2:**
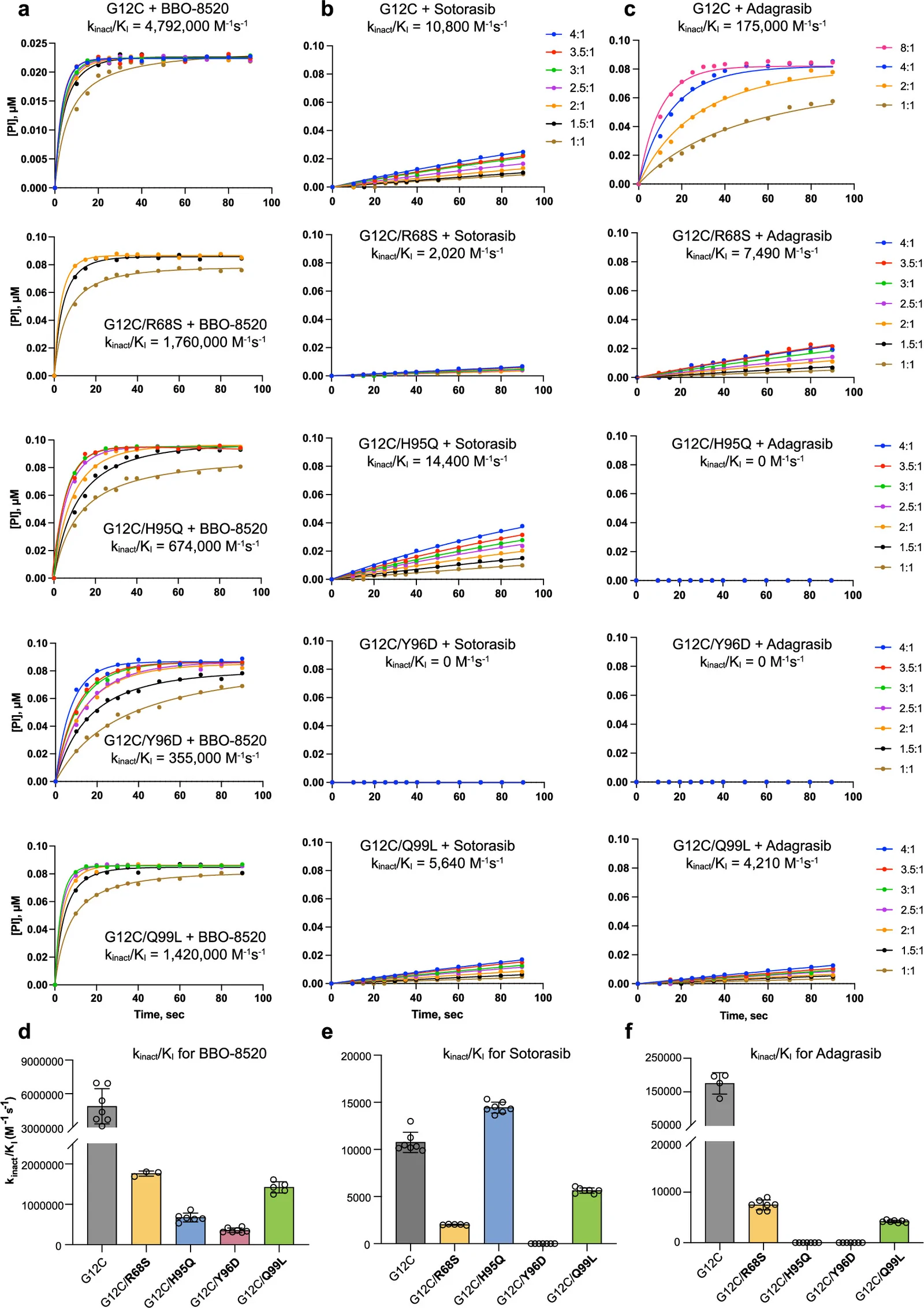
Kinetic characterization of covalent engagement of KRAS G12C and resistance mutants by selected G12C inhibitors. Kinetic parameters (*k*_inact_/*K*_I_), reflecting the overall efficiency of covalent inhibitor engagement, were determined for KRAS G12C and the resistance mutants G12C/R68S, G12C/H95Q, G12C/Y96D, and G12C/Q99L using three inhibitors—BBO-8520, sotorasib, and adagrasib—selected from the broader set shown in Fig. 1. All proteins were analyzed in the GDP-bound state in the C118S background. **a**–**c** Time-course data and fits used to derive *k*_inact_/*K*_I_ values for BBO-8520 (**a**), sotorasib (**b**), and adagrasib (**c**) across KRAS G12C and resistance mutants, measured at multiple compound-to-protein ratios (indicated at the end of each row). Experiments were performed at 0.1 µM protein, except for KRAS G12C with BBO-8520, which was analyzed at 0.025 µM protein due to its markedly high *k*_inact_/*K*_I_ values observed even at the lowest compound-to-protein ratio. **d**–**f** Bar graphs summarizing the mean *k*_inact_/*K*_I_ values ± standard deviation derived from fits to individual datasets for BBO-8520 (**d**), sotorasib (**e**), and adagrasib (**f**). Three or more independent measurements were performed at different compound-to-protein ratios for each KRAS variant–inhibitor condition, with individual *k*_inact_/*K*_I_ values shown as open circles. The y-axis scales are different for each inhibitor to accommodate the differing magnitudes of *k*_inact_/*K*_I_. Source data are provided as a Source Data file.

To quantify the impact of clinically relevant resistance mutations on covalent inhibitor engagement, two FDA-approved KRAS G12C inhibitors, sotorasib and adagrasib, were evaluated alongside the recently reported dual GDP/GTP-state inhibitor BBO-8520. These compounds possess distinct chemical scaffolds and differ in the positioning of their electrophilic warheads relative to C12, enabling direct comparison of mutation-dependent effects on inhibitor binding kinetics. Covalent engagement efficiency was quantified using *k*_inact_/*K*_I_, a second-order rate constant that integrates the affinity of the initial reversible binding step (*K*_I_) with the rate of subsequent covalent modification (*k*_inact_). *k*_inact_/*K*_I_ thus provides an integrated measure of overall covalent engagement efficiency and is particularly sensitive to mutations that weaken the initial noncovalent binding step within the Switch-II pocket. We measured *k*_inact_/*K*_I_ values for KRAS G12C and the resistance mutants R68S, H95Q, Y96D, and Q99L using BBO-8520, sotorasib, and adagrasib (Fig. 2a–f). All measurements were performed using GDP-bound proteins.

BBO-8520 exhibited the highest overall *k*_inact_/*K*_I_ values for KRAS G12C and maintained measurable covalent engagement across all resistance mutants tested (Fig. 2a, d). The value reported here is approximately 2-fold higher than our previous measurement^18^, likely owing to expanded time-course sampling and experimental conditions optimized to better resolve the rapid engagement kinetics of BBO-8520. Compared to G12C, resistance mutations led to moderate, mutation-dependent reductions in engagement efficiency, typically ranging from several-fold to approximately an order of magnitude. Substitutions at R68 and Q99 resulted in smaller decreases, while H95Q and Y96D caused more substantial reductions. Nevertheless, measurable covalent engagement was observed for all mutants.

Sotorasib demonstrated pronounced mutation-specific sensitivity (Fig. 2b, e). Relative to G12C, the Q99L and R68S mutations resulted in five- and two-fold reductions in *k*_inact_/*K*_I_, while H95Q produced a slight increase in apparent engagement efficiency. This modest increase is within the range of experimental variability but may also indicate improved compatibility of glutamine at position 95 with the sotorasib binding pose. In comparison, the Y96D mutation abolished detectable covalent engagement, indicating a substantial disruption of productive inhibitor binding, covalent reaction, or both.

Adagrasib exhibited the highest overall sensitivity to resistance mutations among the three inhibitors examined (Fig. 2c, f). Compared to G12C, the R68S and Q99L mutations led to >95% reductions in *k*_inact_/*K*_I_, while H95Q and Y96D abolished covalent engagement entirely under the conditions tested. These findings suggest that efficient covalent modification by adagrasib requires interactions among multiple Switch-II pocket residues.

Collectively, these results demonstrate that resistance mutations at different positions exert quantitatively distinct effects on covalent KRAS G12C inhibitors and that these kinetic differences largely align with the inhibitor-specific binding modes observed structurally (Fig. 1). BBO-8520 exhibits the highest baseline *k*_inact_/*K*_I_ and retains measurable covalent engagement across all mutants, albeit with moderate to substantial reductions, whereas sotorasib, and particularly adagrasib, show pronounced, mutation-dependent decreases or complete loss of engagement. Across all three inhibitors tested, Y96D confers the greatest overall reduction in engagement. The mutation-specific vulnerability patterns observed here for sotorasib and adagrasib are consistent with orthogonal approaches measuring IC_50_, which also showed that R68S and Y96D confer substantial resistance to divarasib^4,5^. Together, these findings provide a quantitative framework linking mutation position, inhibitor binding mode, and covalent engagement efficiency in KRAS G12C resistance.

### Impact of resistance mutations on noncovalent engagement of Switch-II pocket inhibitors

**Fig. 3:**
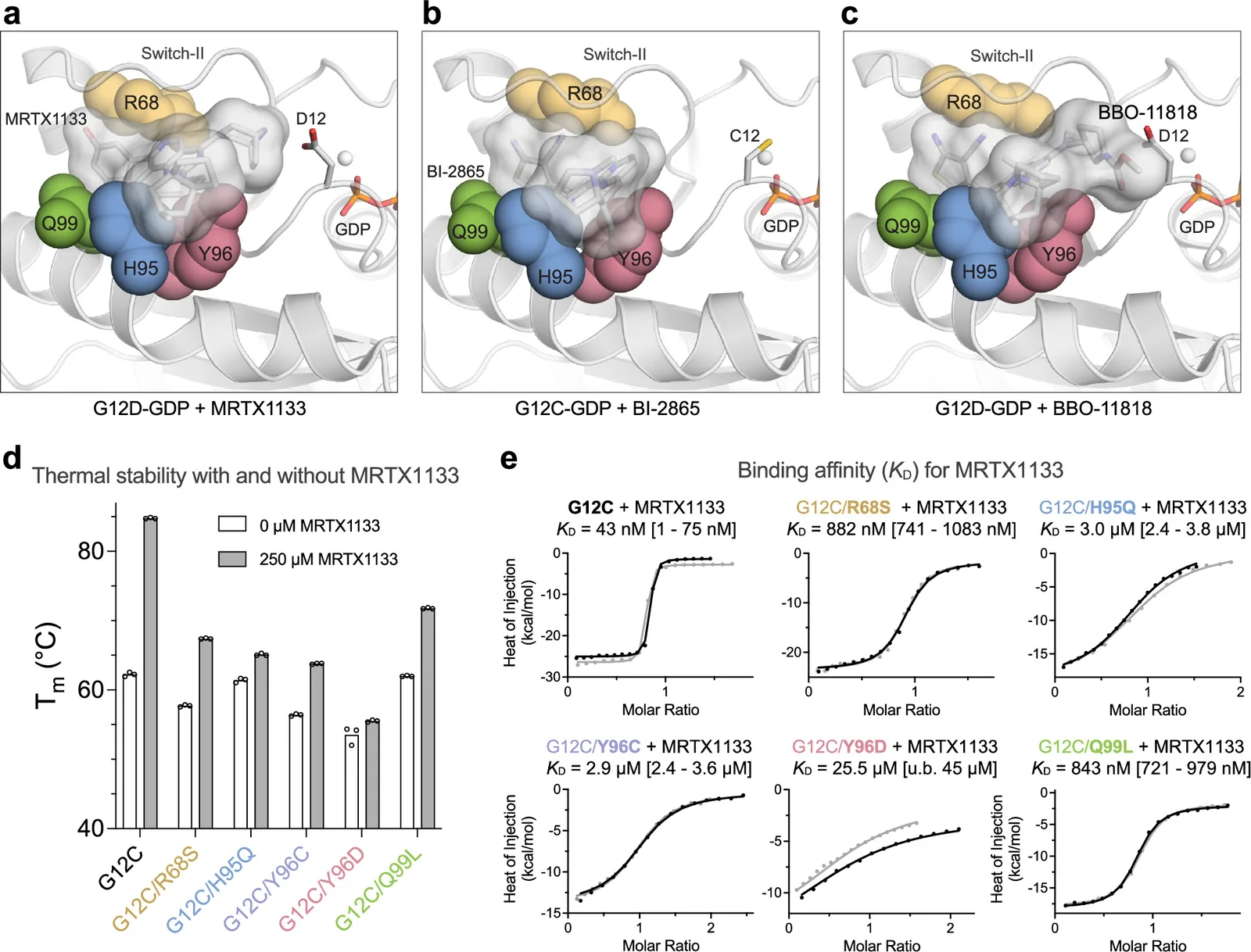
Noncovalent Switch-II pocket inhibitor engagement across KRAS resistance mutations. **(a-c)** Structures of three representative noncovalent direct KRAS inhibitors bound in the Switch-II pocket. **a** MRTX1133 bound to KRAS G12D in the GDP-bound state (PDB 7RPZ); MRTX1133 is a G12D-selective inhibitor that engages both GDP- and GTP-bound KRAS. **b** BI-2865 bound to KRAS G12C-GDP (PDB 8AZX); BI-2865 is a pan-KRAS inhibitor selective for the inactive GDP-bound state. **c** BBO-11818 bound to KRAS G12D-GDP (PDB 9P44); BBO-11818 is a pan-KRAS inhibitor capable of engaging both GDP- and GTP-bound states. In all structures, residues corresponding to resistance sites analyzed in this study are shown as spheres and color-coded as in Fig. 1. **d** Differential scanning fluorimetry (DSF) analysis of GDP-bound KRAS G12C and resistance mutants in the absence and presence of 250 µM MRTX1133. Bar heights represent mean melting temperatures (*T*_m_) from three technical replicates; individual data points are plotted as open circles. **e** Isothermal titration calorimetry (ITC) analysis of MRTX1133 binding to GDP-bound KRAS G12C and resistance mutants. Wiseman plots show integrated heats of injection, with dissociation constants (*K*_D_) and associated 1σ confidence intervals obtained from global fitting of two independent experiments (black and gray curves). For G12C/Y96D, only an upper bound (u.b.) for the confidence interval could be obtained. Source data are provided as a Source Data file.

Because most direct KRAS inhibitors, whether covalent or noncovalent, bind to the Switch-II pocket, noncovalent inhibitors provide a direct readout of how resistance substitutions alter pocket recognition, independent of covalent chemistry. To assess the impact of resistance mutations on engagement of direct KRAS inhibitors at this site, representative noncovalent Switch-II pocket inhibitors were analyzed (Fig. 3a–c). MRTX1133 is a KRAS G12D-selective inhibitor that binds both GDP- and GTP-bound KRAS with high affinity^25^. BI-2865 is a pan-KRAS inhibitor selective for the inactive GDP-bound state^19^, while BBO-11818 is a pan-KRAS inhibitor capable of binding both nucleotide states^26^. Structural comparisons indicate that, although all three inhibitors occupy the Switch-II pocket, they adopt distinct binding poses and interact with the resistance residues R68, H95, Y96, and Q99 in different ways, suggesting that these sites will differentially influence reversible binding.

MRTX1133, a derivative of adagrasib, replaces the piperazine–acrylamide warhead with a bridged piperazine to accommodate G12D, and incorporates a pyridopyrimidine core that preserves the hydrogen bond to H95 while introducing a new hydrogen bond with R68 (Supplementary Fig. 1). BI-2865, derived from BI-0474, lacks the piperazine–acrylamide warhead; its oxadiazole core maintains the H95 hydrogen bond, and a solvent-facing pyrrolidine increases van der Waals contact with H95 relative to BI-0474. BBO-11818, a dual-state pan-KRAS inhibitor derived from BBO-8520, replaces the piperazine–acrylamide warhead with a methyl carbamate that hydrogen bonds to the backbone of residue 12 and contacts Y96 via a water molecule, while retaining the H95 hydrogen bond and the water-mediated interaction with R68.

Although developed as a KRAS G12D inhibitor, MRTX1133 also binds KRAS G12C, as well as several KRAS G12, G13, and Q61 mutants, with high affinity^27^. Structural studies have further shown that its binding pose is highly conserved across KRAS mutants and closely resembles the pre-covalent binding mode of clinical KRAS(G12C) inhibitors. Accordingly, MRTX1133 serves as a well-characterized noncovalent probe for assessing how resistance mutations alter Switch-II pocket recognition, while recognizing that it does not fully recapitulate the binding properties of all covalent inhibitors. The intrinsic thermal stability of KRAS G12C and resistance mutants was evaluated using differential scanning fluorimetry (DSF) in the absence of inhibitor (Fig. 3d and Supplementary Fig. 2). Compared to G12C, the Y96D, Y96C, and R68S mutants exhibited modest decreases in melting temperature, indicating subtle destabilization of the folded state. In contrast, H95Q and Q99L exhibited melting temperatures comparable to G12C. These results demonstrate that substitutions at Switch-II pocket hotspot residues can differentially influence intrinsic KRAS stability, even in the absence of an inhibitor. Addition of MRTX1133 resulted in strong stabilization of KRAS G12C, producing a large increase in melting temperature (>20 °C), consistent with tight noncovalent binding. In contrast, all resistance mutants showed attenuated ligand-induced stabilization, with the magnitude of the Δ*T*_m_ shift varying by mutation. Y96D exhibited the smallest stabilization, followed by H95Q, whereas R68S, Y96C, and Q99L showed intermediate responses.

To quantify these effects, we measured binding affinities using isothermal titration calorimetry (ITC) (Fig. 3e and Supplementary Fig. 3). KRAS G12C bound MRTX1133 with low-nanomolar affinity (*K*_D_ ≈ 43 nM), whereas resistance mutations produced graded losses in binding strength (Supplementary Fig. 4). R68S and Q99L weakened binding by ~20-fold, H95Q and Y96C by ~70–80-fold, and Y96D by ~600-fold relative to G12C. Together, the DSF and ITC data demonstrate that resistance mutations impair noncovalent binding of MRTX1133 to the Switch-II pocket in a mutation-dependent manner. Similar trends observed in ligand-induced thermal stabilization and binding affinity are consistent with progressive weakening of noncovalent engagement at the Switch-II pocket as a function of mutation. Although the current data do not allow us to determine the relative contributions of altered reversible binding (*K*_I_) and covalent bond formation (*k*_inact_) to the observed resistance phenotypes, the strong concordance between the noncovalent MRTX1133 binding data and the defects in overall binding efficiency (reflecting the noncovalent and covalent contributions) observed across multiple KRAS(G12C) inhibitors suggests that disruption of reversible inhibitor binding is a major contributor to resistance conferred by mutations at these sites. These findings provide a mechanistic basis for the differential kinetic defects observed with the covalent KRAS G12C inhibitors (Fig. 2).

### Distinct structural rearrangements induced by Y96D underlie resistance in both the inactive and active states

**Fig. 4:**
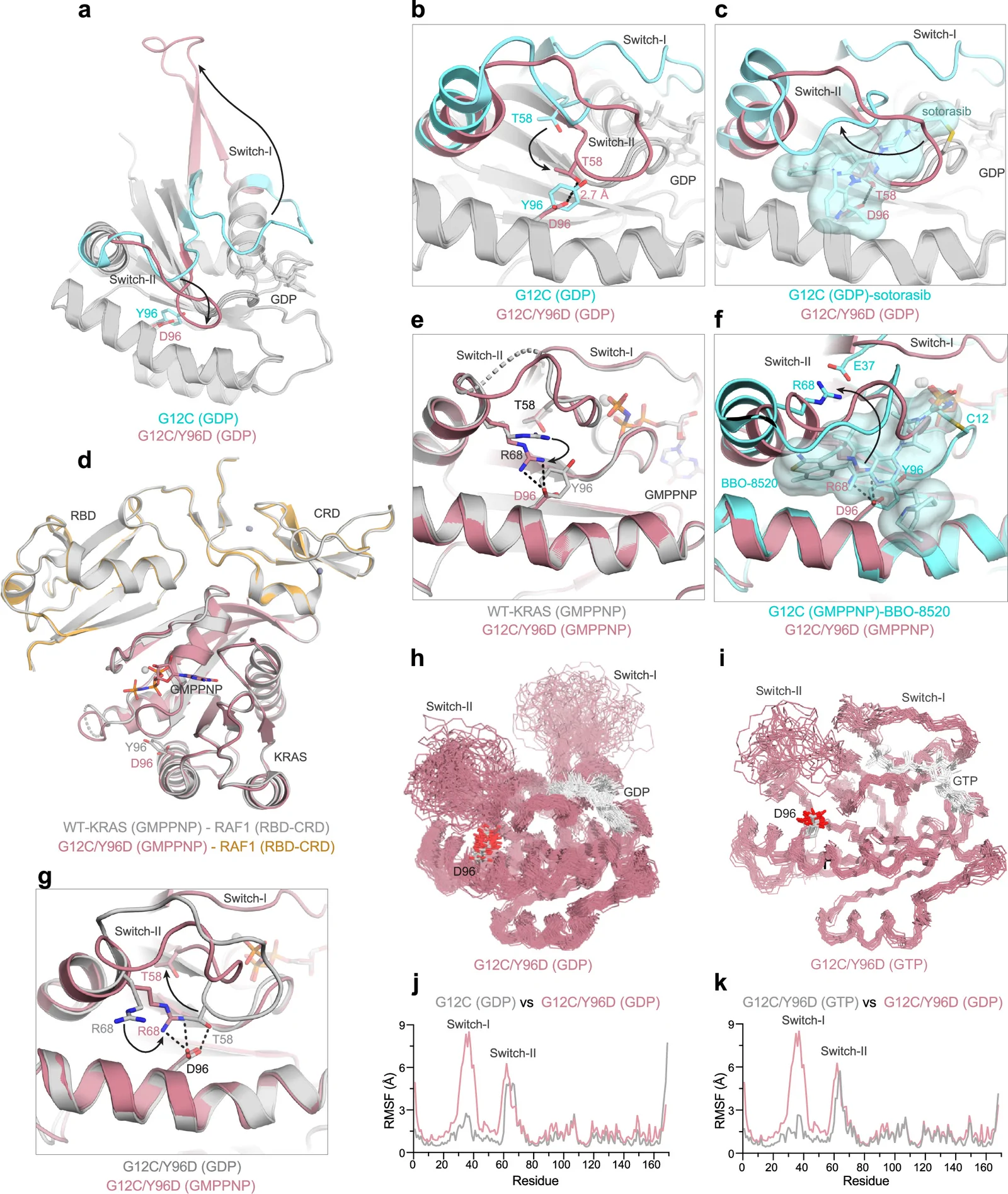
Structural remodeling and conformational dynamics of the KRAS Y96D resistance mutation in the inactive and active states. **a** Structure of KRAS G12C/Y96D bound to GDP solved in this study, superimposed on KRAS G12C-GDP (PDB 4LDJ). Regions of structural similarity are shown in gray, while regions of divergence are colored pink for G12C/Y96D and cyan for G12C. Notable differences are observed in Switch-I and in regions proximal to Switch-II. The side chain at position 96 is shown as sticks. **b** Close-up view of the local structural rearrangement induced by the Y96D mutation in the GDP-bound state. D96 forms a non-native hydrogen bond with the side chain of T58 (2.7 Å), pulling the main chain into the Switch-II pocket. In the G12C control, the native Y96 hydroxyl is 6.7 Å away from the T58 hydroxyl. **c** Superposition of KRAS G12C/Y96D-GDP (pink) with KRAS G12C-GDP covalently bound to sotorasib (cyan; PDB 6OIM). Sotorasib is shown as cyan sticks within the transparent surface. The non-native D96-T58 interaction draws the backbone around T58 into the Switch-II pocket, causing steric occlusion of the sotorasib binding site and accounting for the maximal resistance conferred by the Y96D mutation. **d** Superposition of KRAS G12C/Y96D-GMPPNP bound to RAF1 RBD-CRD (pink and orange), solved in this study, with the WT KRAS-GMPPNP-RAF1 RBD-CRD complex (gray; PDB 6XHB). The near-identical alignment, with only localized conformational differences around Y96D, indicates that the resistance mutation does not impact effector binding in the active, GTP-bound state. **e** Close-up view of the Y96D region in the GMPPNP-bound state. The RAF1 RBD-CRD is omitted for clarity. In contrast to the GDP-bound structure, T58 no longer interacts with D96; instead, R68 forms a non-native salt bridge with D96 that is absent in the structure of WT or the G12C mutant of KRAS. **f** Superposition of KRAS G12C/Y96D-GMPPNP (pink) from the RAF1 RBD-CRD complex with KRAS G12C-GMPPNP covalently bound to BBO-8520 (cyan; PDB 8V39). BBO-8520 is shown as sticks within the transparent surface. In the G12C structure, R68 forms a salt bridge with E37; in the Y96D mutant, the non-native D96-R68 interaction occupies overlapping space with the bound inhibitor, explaining the reduced efficacy of active-state Switch-II pocket inhibitors. **g** Superposition of KRAS G12C/Y96D in the GDP- and GMPPNP-bound states solved in this study, illustrating nucleotide-dependent conformational switching and altered interactions with the mutant D96. The RAF1 RBD-CRD is omitted for clarity. In the GDP-bound state, the D96-T58 interaction stabilizes a Switch-II conformation that occludes the inhibitor-binding pocket, whereas in the GMPPNP-bound state, this interaction is replaced by a D96-R68 salt bridge, allowing Switch-II to adopt a conformation compatible with effector engagement. **h, i** Representative structural ensembles from five independent 1-µs molecular dynamics simulations of KRAS G12C/Y96D in the GDP-bound (**h**) and GMPPNP-bound (**i**) states. The D96 side chain is shown as sticks, and the bound nucleotide is depicted as white sticks. **j** Comparison of root-mean-square fluctuations (RMSF) between KRAS G12C/Y96D-GDP and the KRAS G12C-GDP control from five independent 1-µs simulations. **k** Comparison of RMSF between KRAS G12C/Y96D in the GDP- and GMPPNP-bound states from five independent 1-µs simulations. The GMPPNP-bound simulations were initiated from the structure of KRAS G12C/Y96D as found in the RAF1 RBD-CRD complex, revealing suppression of Switch-I dynamics in the active state. Source data are provided as a Source Data file.

Kinetic and biophysical analyses (Figs. 2 and 3) demonstrate that Y96D produces the most severe impairment of inhibitor engagement among the resistance mutations tested. To define the structural basis of this effect, we determined the structure of G12C/Y96D in both the inactive and active states. The G12C/Y96D-GDP structure was solved at 1.5 Å resolution; composite omit electron density for the mutated residue is shown in Supplementary Fig. 5. Superposition with KRAS G12C-GDP (Fig. 4a) shows preservation of the overall fold, but pronounced conformational differences localized to Switch-I and Switch-II. In G12C/Y96D, Switch-I adopts a fully open conformation, characterized by the formation of non-native antiparallel β-strands decoupled from the G-domain core.

The critical structural change occurs within Switch-II (Fig. 4b). The mutant D96 side chain forms a non-native hydrogen bond (2.7 Å) with the hydroxyl group of T58; in the G12C control structure, Y96 and T58 are separated by >6 Å. This D96-T58 interaction pulls the Switch-II backbone toward the Switch-II/helix α3 drug-binding pocket, partially occluding the cavity. Superposition with sotorasib-bound G12C-GDP (Fig. 4c), as well as with structures containing covalently bound adagrasib and BBO-8520 and noncovalently bound MRTX1133 (Supplementary Fig. 6), shows that the repositioned Switch-II segment directly overlaps the space occupied by the various inhibitors. This structural occlusion explains both the substantial loss of covalent engagement for BBO-8520 and unmeasurable engagement for sotorasib and adagrasib (Fig. 2), as well as the ~600-fold reduction in MRTX1133 affinity relative to G12C (Fig. 3e).

To assess whether the fully open Switch-I conformation observed in the crystal structure persists in solution, the ^1^H-^15^N HSQC spectra of G12C/Y96D-GDP and G12C-GDP were compared (Supplementary Fig. 7). The Y96D mutation induces widespread chemical shift perturbations, including residues in Switch-I and Switch-II, suggesting that the observed conformational rearrangements are not solely crystal lattice-induced. The largest spectral changes are observed proximal to the nucleotide binding site and Switch-II, with more modest perturbations in Switch-I. Thus, the NMR data are not definitive as to whether the fully open Switch-I conformation captured in the crystal is significantly populated in solution. Nevertheless, they show that the Y96D mutation induces both structural perturbations and conformational dynamics absent in the G12C control. The fully open Switch-I conformation observed in the G12C/Y96D crystal has been reported in multiple GDP-bound KRAS structures and is consistently associated with crystallization in space group *P*321 with similar unit cell parameters, suggesting that this lattice is diagnostic of the open state (Supplementary Fig. 8). As in prior examples, GDP density is well defined, whereas Mg²⁺ is absent, consistent with disruption of the ion’s coordination network in the fully open Switch-I conformation. Critically, the Switch-II arrangement in G12C/Y96D remains distinct from Switch-II conformations observed in other open Switch-I structures, indicating that the intrusion of Switch-II into the drug-binding pocket reflects Y96D-specific effects rather than simply adoption of the fully open Switch-I state.

We next examined the active state. Although isolated GMPPNP-bound G12C/Y96D could not be crystallized despite extensive screening, we determined the structure of G12C/Y96D-GMPPNP in complex with the RAF1 RBD-CRD (Fig. 4d). The mutant-effector complex closely superimposes with the WT KRAS-RAF1 RBD-CRD structure (backbone RMSD 0.22 Å), indicating preservation of the global active-state architecture. However, local interactions involving residue 96 differ markedly from those in the GDP-bound state (Fig. 4e). In the GMPPNP-bound structure, D96 no longer hydrogen bonds with T58 (~7 Å separation) but instead forms a ~3 Å salt bridge with R68. In WT KRAS, the larger Y96 side chain restricts R68 rotamer positioning; substitution with Asp allows R68 to adopt a position creating a stabilizing salt bridge with the smaller D96 side chain, an interaction previously predicted by simulations of the modeled structure of G12C/Y96D–GDP^28^. Superposition of G12C/Y96D-GMPPNP with GMPPNP-bound G12C covalently modified by BBO-8520 (Fig. 4f) shows that both the D96-R68 salt bridge and the repositioned Switch-II backbone encroach upon the Switch-II pocket. In the presence of native Y96 and bound BBO-8520, R68 cannot occupy the Switch-II pocket, but instead makes a salt bridge with E37 from Switch-I. Thus, Y96D induces distinct structural rearrangements in both the inactive and active states: a D96-T58 hydrogen bond draws Switch-II into the pocket in the GDP-bound state, but upon transition to the active state, a D96-R68 salt bridge replaces the D96-T58 hydrogen bond, abolishing the non-native conformation of Switch-II and allowing it to assume a conformation competent for effector binding (Fig. 4g). In the presence of either nucleotide, productive inhibitor binding requires disruption of energetically favorable non-native hydrogen-bonding or electrostatic interactions that result in partial occlusion of the binding cavity. The need to break these stabilizing contacts increases the energetic barrier for pocket opening and inhibitor accommodation, providing a structural rationale for why Y96D confers the strongest resistance phenotype among the mutations examined.

To further characterize conformational dynamics, five independent 1-µs all-atom molecular dynamics simulations were performed for GDP- and GMPPNP-bound G12C/Y96D starting from the experimentally determined structures (GMPPNP was modified to GTP). Representative conformational states are overlaid in Fig. 4h, i. In the GDP-bound state, the largest fluctuations localize to the Switch regions, particularly Switch-I, consistent with the fully open crystallographic conformation. RMSF analysis (Fig. 4j) shows increased Switch-I mobility relative to G12C-GDP, whereas Switch-II fluctuations are modestly elevated. Simulations initiated from the GTP-bound structure (RAF1 RBD-CRD omitted) reveal marked suppression of Switch-I dynamics compared to the GDP-bound state (Fig. 4k), while Switch-II fluctuations remain similar between nucleotide states. Importantly, H-bond interaction analyses corroborate the structural observations: the D96-T58 interaction in the GDP-bound state persists for 76%–90% of the time in 3 out of 5 simulations and more than 33% of the time in all replicas, while the D96–R68 salt bridge in the GTP-bound state is maintained for 65%–95% of the time in 4 out of 5 simulations and more than 34% of the time in all replicas (Supplementary Table 2). Although the GDP-bound fully open Switch-I conformation exhibits increased dynamics, the presence of the γ-phosphate in GTP stabilizes a binding-competent active conformation. Together, these structural and dynamical analyses show that Y96D confers resistance by stabilizing pocket-occluding conformations of Switch-II in both inactive and active states, thereby reducing inhibitor access and binding regardless of which nucleotide is bound.

### Structural perturbations and altered dynamics in KRAS resistance mutants Y96C, R68S, H95Q, and Q99L

**Fig. 5:**
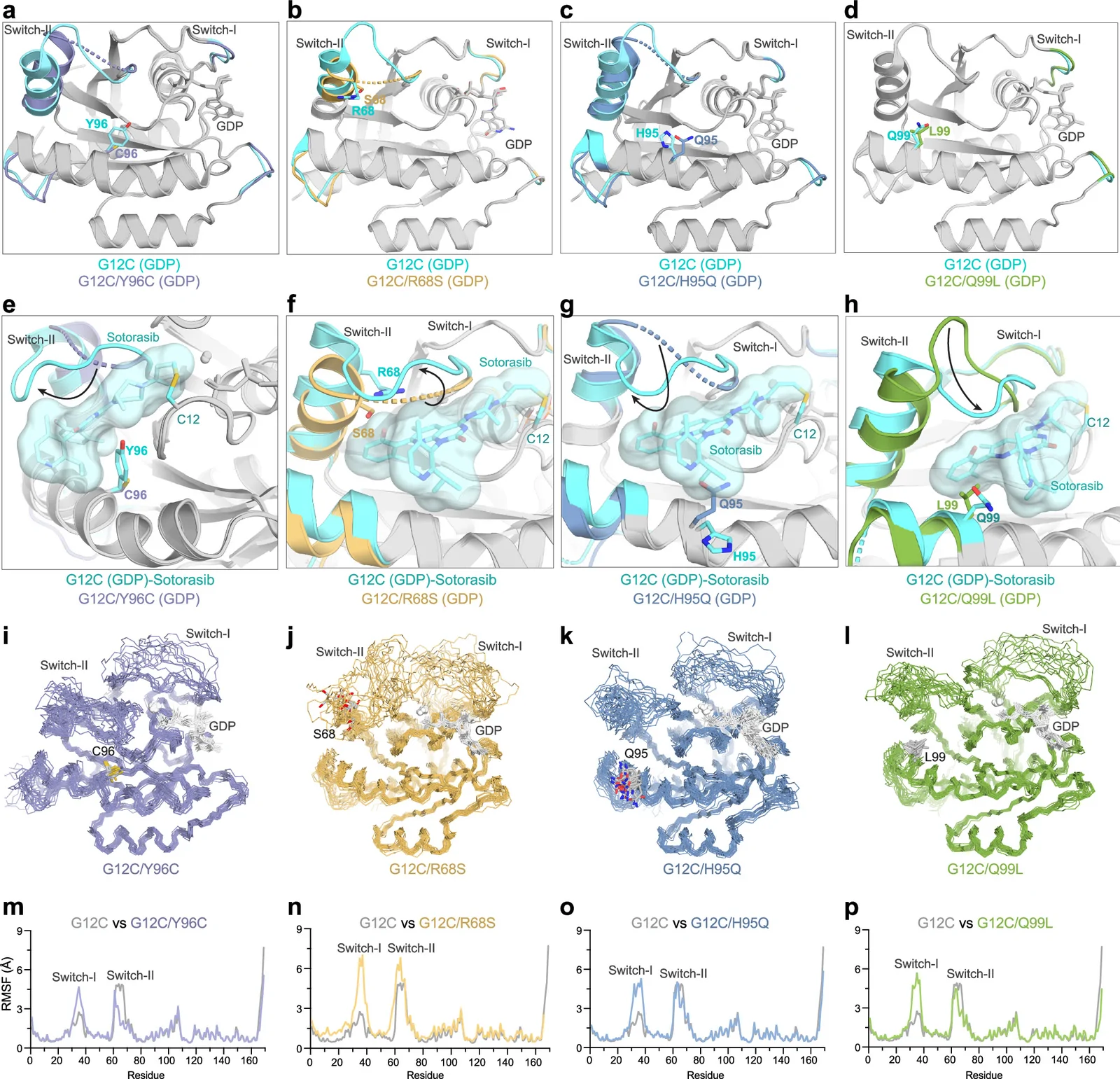
Structural and dynamical consequences of resistance mutations R68S, Y96C, H95Q, and Q99L within the KRAS G12C Switch-II pocket. Structural superpositions of the resistance mutants G12C/Y96C-GDP (**a**), G12C/R68S-GDP (**b**), G12C/H95Q-GDP (**c**), and G12C/Q99L-GDP (**d**) solved in this study with the previously reported KRAS G12C-GDP structure (PDB 4LDJ). Regions of structural similarity are shown in gray, while regions of divergence are colored cyan in the G12C control and purple (Y96C), yellow (R68S), blue (H95Q), or green (Q99L) in the corresponding mutant structures. Side chains at the sites of mutation are shown as sticks. Structural superpositions of the same resistance mutants—G12C/Y96C-GDP (**e**), G12C/R68S-GDP (**f**), G12C/H95Q-GDP (**g**), and G12C/Q99L-GDP (**h**)—with KRAS G12C-GDP covalently bound to sotorasib (PDB 6OIM). Regions of similarity are shown in gray, while structural differences are colored cyan in the G12C-GDP-sotorasib structure and purple, yellow, blue, or green in the corresponding mutant structures. Sotorasib is shown as cyan sticks within a transparent cyan surface, illustrating how each resistance mutation perturbs the Switch-II pocket relative to the drug-bound conformation. Representative structural ensembles from five independent 1-µs molecular dynamics simulations of G12C/Y96C-GDP (**i**), G12C/R68S-GDP (**j**), G12C/H95Q-GDP (**k**), and G12C/Q99L-GDP (**l**). Comparison of root-mean-square fluctuations (RMSF) for each resistance mutant—G12C/Y96C-GDP (**m**), G12C/R68S-GDP (**n**), G12C/H95Q-GDP (**o**), and G12C/Q99L-GDP (**p**)—relative to KRAS G12C-GDP, derived from five independent 1-µs molecular dynamics simulations, highlighting mutation-specific effects on Switch-I and Switch-II dynamics. Source data are provided as a Source Data file.

To define how additional recurrent resistance mutations reshape the Switch-II pocket, we determined crystal structures of GDP-bound KRAS G12C/Y96C, G12C/R68S, G12C/H95Q, and G12C/Q99L, and compared them to the previously reported KRAS G12C-GDP structure (Fig. 5a–d). In all four cases, the overall fold and Switch-I conformation are largely preserved, and structural differences localize primarily to the Switch-II pocket and adjacent regions. The Y96C, R68S, and H95Q mutants crystallized in space groups distinct from G12C, whereas Q99L crystallized in the same space group and with nearly identical unit cell parameters, permitting close backbone comparison.

In the Y96C structure (Fig. 5a), substitution of Y96 with cysteine removes the aromatic ring without introducing new polar interactions, unlike the distinct non-native interaction observed for Y96D (Fig. 4b). In the G12C structure, Y96 contributes hydrophobic and π-stacking interactions to multiple Switch-II pocket inhibitors, including sotorasib and adagrasib, and supports van der Waals contacts across diverse scaffolds. Replacement with cysteine eliminates these aromatic interactions while preserving backbone geometry. In the Y96C structure, a water molecule occupies part of the space previously filled by the larger Y96 side chain. A hydrogen bond between R68 and Q99 (~3.4 Å) is observed, but no non-native interaction draws Switch-II into the pocket. Thus, Y96C primarily weakens hydrophobic and stacking interactions with inhibitors, consistent with a moderate resistance phenotype.

Like Y96, R68 plays an important architectural role in shaping the Switch-II pocket. In inhibitor-bound structures, the large, positively charged arginine side chain forms a salt bridge with E37 (or an indirect interaction via water in the case of sotorasib), stabilizing Switch-I in a conformation incompatible with effector binding, helping define the pocket contour, and stabilizing local inter-residue interactions that couple Switch-II to helix α3. Additionally, R68 makes van der Waals contacts with bound inhibitors and, in some cases, hydrogen bonding or cation–π interactions. The R68S mutation (Fig. 5b) replaces this bulky, positively charged residue with a smaller, polar serine, thereby eliminating positive charge and introducing a new S68-Q99 interaction. This substitution precludes the formation of a salt bridge with E37, alters local contacts, and reduces steric support at the pocket entrance, thereby broadening conformational fluctuations within the Switch region. Both the crystal structure and simulations described below indicate that R68S perturbs Switch-II architecture more extensively than mutations that primarily disrupt protein-ligand contacts.

For H95Q (Fig. 5c), substitution of histidine at position 95 with glutamine preserves polarity but alters side-chain geometry and interaction potential. In most G12C inhibitor complexes, except for opnurasib and sotorasib-like compounds, H95 forms direct contacts with ligand functional groups or contributes to shaping the base of the pocket. In the H95Q structure, no major backbone rearrangements are observed, and the side chain of Q95 adopts a conformation different from that of H95 in G12C. Accordingly, resistance in this mutant likely reflects disruption of specific side chain-inhibitor interactions rather than global pocket remodeling.

In the Q99L structure (Fig. 5d), the backbone conformation is essentially indistinguishable from G12C. In most inhibitor-bound structures, except for sotorasib, the polar Q99 side chain is stabilized by a hydrogen bond with R102. Although L99 can adopt rotamers that do not occlude the inhibitor binding site, loss of the Q99-R102 hydrogen bond likely increases local flexibility and destabilizes the pocket. In the sotorasib-bound structure, Q99 does not hydrogen bond with R102, but instead contributes polar character at the Switch-II/helix α3 interface to accommodate the hydroxyl group on sotorasib. Overall, the Q99L mutation subtly alters local packing and removes polar character that can contribute to inhibitor positioning.

Superposition of each mutant with the sotorasib-bound G12C-GDP structure (Fig. 5e–h) shows that none of these substitutions produces the pronounced pocket occlusion observed for Y96D. Instead, each mutation weakens inhibitor binding through different mechanisms: Y96C removes aromatic stacking; R68S eliminates key electrostatic interactions and alters pocket dynamics in a way that increases the population of conformers less favorable to drug binding; H95Q modifies potential contacts at the base of the cavity; and Q99L disrupts polar and water-mediated interactions. These modest structural perturbations are reflected in less severe reductions in covalent and noncovalent drug engagement observed biochemically compared with the Y96D mutant.

To assess mutation-dependent effects on conformational dynamics, we performed five independent 1-µs all-atom molecular dynamics simulations for each GDP-bound mutant (Fig. 5i–l). RMSF analysis relative to G12C-GDP (Fig. 5m–p) reveals mutation-specific alterations in Switch-I and Switch-II flexibility. Y96C shows modest increases in Switch-I and Switch-II mobility, consistent with reduced packing at the pocket base. R68S exhibits the largest fluctuations in both Switch regions, supporting the structural observation that loss of arginine at position 68 perturbs broader Switch-II architecture, with the effects also propagating to Switch-I and resulting in the largest Switch-I fluctuations for any resistance mutant other than Y96D. H95Q and Q99L display moderate changes in Switch dynamics, with localized differences near the end of helix α3. Collectively, these data indicate that Y96C, R68S, H95Q, and Q99L confer resistance through mutation-specific remodeling of Switch-II pocket contacts and local dynamics, in contrast to the overt change in Switch-II conformation caused by Y96D. These substitutions primarily weaken key residue-inhibitor interactions and, in the case of R68S, disrupt the electrostatic and steric framework that defines the Switch-II/helix α3 interface, leading to broader destabilization of the Switch-II pocket architecture rather than eliminating a single contact.

### Resistance mutations differentially modulate binding to effectors RAF1 and PI3Kα

**Fig. 6:**
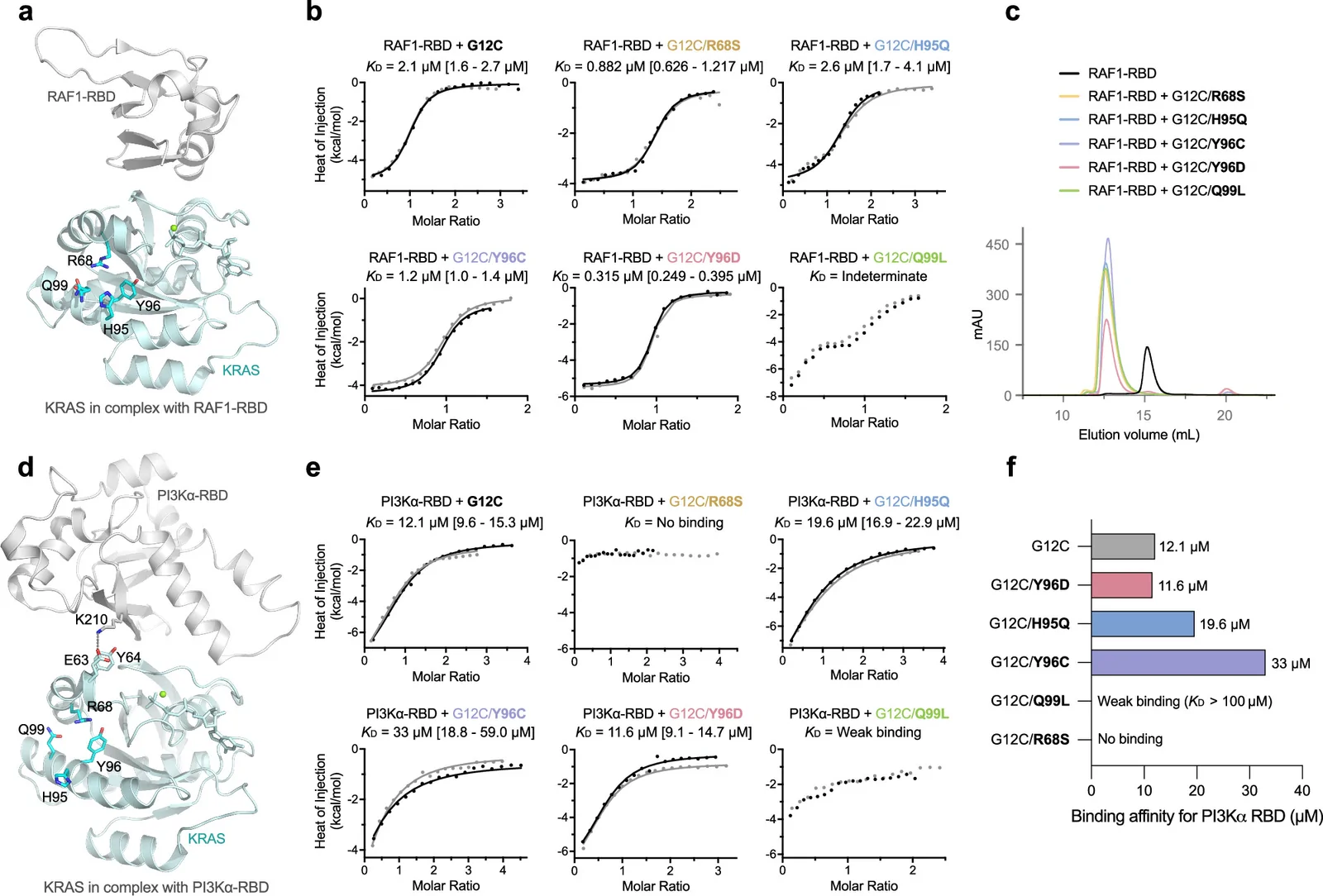
Binding of KRAS resistance mutants to RAF1 and PI3Kα effectors. **a** Structure of KRAS bound to the RAF1 RBD (PDB 6VJJ). Side chains corresponding to the resistance mutation sites analyzed in this study are labeled and shown as cyan sticks. **b** ITC analysis of RAF1 RBD binding to GMPPNP-bound KRAS control and resistance mutants. Wiseman plots show integrated heats of injection, with dissociation constants (*K*_D_) and their associated 1σ confidence intervals obtained from global fitting of two independent experiments (black and gray curves). **c** Analytical size-exclusion chromatography (Superdex 75) was used as an orthogonal approach to assess complex formation between the RAF1 RBD and KRAS resistance mutants. **d** Structure of KRAS bound to the PI3Kα RBD (PDB 9C15). Side chains corresponding to the resistance mutation sites are labeled and shown as cyan sticks. In contrast to RAF1, the PI3Kα RBD engages residues within Switch-II of KRAS, including E63 and Y64, through direct contacts involving K210 and neighboring residues. **e** ITC analysis of PI3Kα RBD binding to GMPPNP-bound KRAS control and resistance mutants. Wiseman plots show integrated heats of injection, with dissociation constants (*K*_D_) and their associated 1σ confidence intervals obtained from global fitting of two independent experiments (black and gray curves). **f** Summary of binding affinities for interactions between the PI3Kα RBD and KRAS control or resistance mutants derived from ITC measurements. Source data are provided in the Source Data file.

To determine how resistance mutations that impair Switch-II pocket inhibitor engagement affect downstream signaling competence, we examined the binding of KRAS resistance mutants to two key effectors, RAF1 and PI3Kα. The structures of KRAS in complex with the RAF1 RBD (Fig. 6a) and the PI3Kα RBD (Fig. 6d) show that the resistance hotspot residues R68, H95, Y96, and Q99 are largely distal from the core RAF1 binding interface, which is dominated by interactions with Switch-I^29^. In contrast, the PI3Kα RBD engages both Switch-I and Switch-II, positioning these resistance sites closer to the PI3Kα interaction surface, particularly R68. Specifically, PI3Kα makes direct contacts with Switch-II residues E63 and Y64^30^, suggesting that mutations within this region are more likely to influence PI3Kα engagement than RAF1 binding.

To quantitatively assess these effects, we measured binding of GMPPNP-loaded KRAS G12C and resistance mutants in the G12C background to RAF1 and PI3Kα using ITC. Previous studies by surface plasmon resonance (SPR) and other methods have shown that KRAS G12C binds the RAF1 RBD with an affinity comparable to that of wild-type KRAS (*K*_D_ of 60–300 nM for G12C vs. 50–250 nM for WT KRAS in various buffer conditions)^27,31^. In contrast, ITC measurements report a weaker apparent affinity for KRAS G12C (*K*_D_ ~2 µM), suggesting that the reduced affinity observed by ITC reflects methodological effects rather than truly impaired effector engagement.

ITC measurements revealed that all resistance variants retained binding to the RAF1 RBD (Fig. 6b). Among these, Y96D displayed the strongest interaction, with a *K*_D_ of 0.32 µM, followed by R68S (*K*_D_ = 0.88 µM), Y96C (*K*_D_ = 1.2 µM), and H95Q (*K*_D_ = 2.6 µM). Q99L produced a biphasic ITC isotherm that could not be reliably fitted to a 1:1 interaction model, likely suggesting complex binding behavior, but still indicating interaction with the RAF1 RBD. Thermograms and Wiseman plots for all RAS-RAF1 RBD ITC measurements are given in Supplementary Fig. 9. To validate the ITC results, we employed analytical size-exclusion chromatography and SDS-PAGE as an orthogonal assay for complex formation. In agreement with the calorimetric data, all resistance variants, including Q99L, co-eluted with the RAF1 RBD (Fig. 6c and Supplementary Fig. 10), confirming stable complex formation across all substitutions examined.

In contrast to RAF1 binding, binding to the PI3Kα RBD was markedly more sensitive to resistance mutations (Figs. 6e and 6f). KRAS G12C-bound PI3Kα with a *K*_D_ of 12.1 µM. Several resistance substitutions weakened substantially or abolished this interaction altogether. R68S exhibited no detectable binding, while Q99L showed only weak binding (estimated *K*_D_ > 100 µM). H95Q and Y96C bound with *K*_D_ values of 19.6 and 33 µM, representing ~2-fold and ~3-fold decreases, respectively. Conversely, Y96D bound PI3Kα with a *K*_D_ of 11.6 µM, essentially indistinguishable from G12C. Thermograms and Wiseman plots for all RAS-PI3Kα RBD ITC measurements are given in Supplementary Fig. 11.

Together, these data demonstrate that resistance mutations exert pathway-selective effects on effector engagement in the KRAS G12C context. RAF1 binding is largely preserved across resistance variants, whereas PI3Kα binding is selectively compromised by substitutions proximal to the Switch-II interface, particularly R68S and Q99L. Notably, Y96D, which confers the strongest resistance to Switch-II pocket inhibitors, retains near-native binding to both RAF1 and PI3Kα. These findings indicate that resistance mutations can decouple inhibitor sensitivity from effector engagement, providing a mechanistic basis for the differential signaling responses observed downstream of KRAS.

### Mutation- and inhibitor-dependent modulation of MAPK signaling downstream of KRAS G12C

**Fig. 7:**
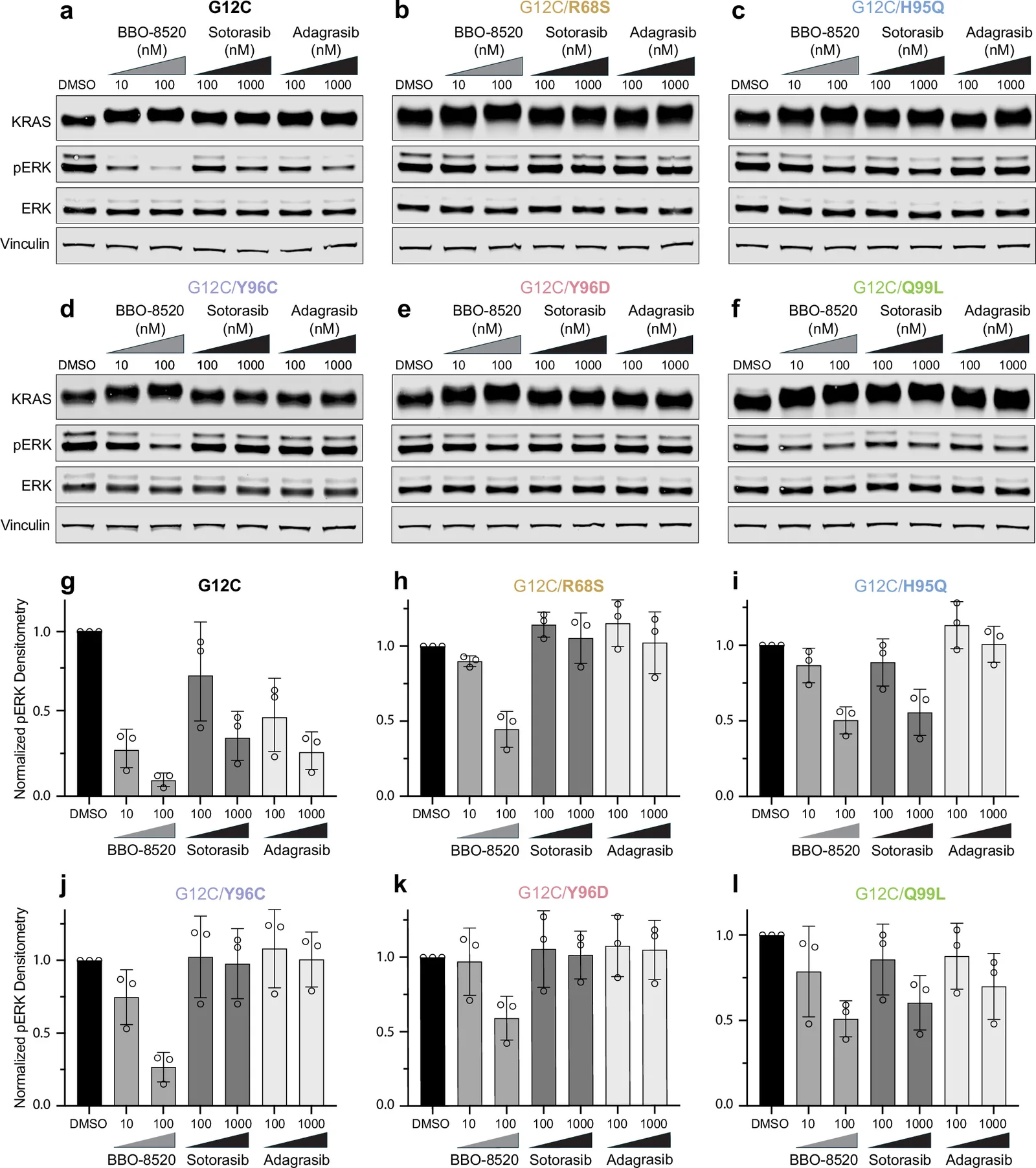
Differential MAPK pathway suppression by KRAS G12C inhibitors across resistance mutants. Representative Western blot analysis of ERK1/2 phosphorylation in HEK293 cells transiently expressing KRAS4b variants following treatment with the covalent KRAS G12C inhibitors BBO-8520, sotorasib, and adagrasib. Cells expressing **a** G12C, **b** G12C/R68S, **c** G12C/H95Q, **d** G12C/Y96C, **e** G12C/Y96D, and **f** G12C/Q99L were treated for 2 h with either DMSO or inhibitor at the indicated concentrations (BBO-8520 at 10 or 100 nM; sotorasib and adagrasib at 100 or 1000 nM). Levels of phosphorylated ERK1/2 (pERK1/2) and total ERK1/2 were assessed by immunoblotting. KRAS band upshift indicates covalent target engagement, with band broadening indicating a partial C12 population labeled with the drug in resistant mutants. Vinculin is shown as a loading control. The mean pERK band intensity and standard deviation from three biological replicates are plotted for **g** G12C, **h** G12C/R68S, **i** G12C/H95Q, **j** G12C/Y96C, **k** G12C/Y96D, and **l** G12C/Q99L; individual data points are plotted as open circles. For each mutant, the pERK band intensity in the presence of drug was normalized to the corresponding DMSO-treated control. Uncropped blots with labeled molecular weight standards are provided in the Source Data file.

Effector-binding measurements indicated that resistance mutations largely preserve interactions with downstream partners in vitro. To determine whether these mutations nonetheless alter signaling output in cells, we examined MAPK pathway suppression by covalent KRAS G12C inhibitors across the same panel of resistance variants. ERK1/2 phosphorylation was analyzed in HEK293 cells transiently expressing KRAS G12C or G12C-based resistance variants following treatment with BBO-8520, sotorasib, or adagrasib. Cells were treated for 2 h with increasing inhibitor concentrations, BBO-8520 at 10 and 100 nM, and sotorasib and adagrasib at 100 nM and 1 µM, and MAPK pathway activity was assessed by immunoblotting for phosphorylated ERK1/2 (pERK). Total ERK1/2 and vinculin served as expression and loading controls (Fig. 7a–f). In cells expressing KRAS G12C, all three inhibitors reduced ERK phosphorylation in a dose-dependent manner (Fig. 7a). Treatment caused an upward shift in the KRAS band, consistent with covalent target engagement. BBO-8520 achieved robust suppression at lower concentrations, whereas sotorasib and adagrasib required higher doses and produced less complete inhibition, consistent with their potency differences and distinct biochemical engagement profiles.

Resistance mutations reduced target engagement and diminished MAPK pathway suppression in a mutation- and inhibitor-dependent manner. Figure 7g–l shows the mean and standard deviation of pERK quantitation from three biological replicates at increasing drug concentrations; for each resistance mutant, the data are normalized to the DMSO-only control. BBO-8520 retained measurable activity in all mutants. At 100 nM, residual pERK relative to control followed the trend Y96D (59%) > R68S, H95Q, Q99L (~45–51%) > Y96C (26%), indicating that none of the substitutions completely abrogated MAPK inhibition by this compound. In comparison to BBO-8520, sotorasib was more sensitive to resistance mutations. At 1 µM, there was essentially no pERK suppression with R68S, Y96C, or Y96D, but sotorasib retained moderate efficacy against the H95Q and Q99L mutants (~56%–60% residual pERK). Adagrasib exhibited the greatest overall vulnerability to resistance mutations. At 1 µM, pERK suppression was nearly abolished for all mutants except Q99L (~70%).

From a resistance mutation-centric perspective, Y96D and R68S confer the most pronounced signaling resistance, particularly to sotorasib and adagrasib, despite preserved KRAS expression and pathway activation in DMSO controls. Y96D produces MAPK pathway impairment across three inhibitors, whereas R68S shows severe insensitivity to sotorasib and adagrasib but retains partial responsiveness to BBO-8520. Y96C is also insensitive to sotorasib and adagrasib but remains highly responsive to BBO-8520. H95Q demonstrates scaffold-selective resistance, strongly resisting adagrasib while allowing moderate pERK suppression by BBO-8520 and sotorasib. Q99L represents the least severe phenotype overall, maintaining substantial responsiveness to all three inhibitors and exhibiting only partial attenuation of pERK suppression at the highest doses tested.

We also examined AKT phosphorylation (pAKT) as a readout of PI3Kα signaling. In KRAS G12C-expressing cells, the highest doses of BBO-8520, sotorasib, and adagrasib modestly reduced pAKT levels to 79%–86% of control (Supplementary Fig. 12), consistent with our prior report^18^. Across the resistance mutants, pAKT responses remained considerably less pronounced than the corresponding effects on pERK. BBO-8520 retained modest pAKT suppression, reducing pAKT levels to approximately 63%–80% of control depending on the mutant, whereas sotorasib and adagrasib generally produced little or no reduction in pAKT. These findings indicate that PI3Kα/AKT signaling is less sensitive than MAPK signaling to KRAS(G12C) inhibition in this cellular context. This behavior is consistent with the partial dependence of AKT signaling on RAS–PI3Kα engagement, while also reflecting contributions from additional upstream inputs and compensatory signaling pathways. Overall, these results demonstrate that resistance mutations selectively relieve MAPK inhibition by KRAS G12C inhibitors, with effects that depend on both the specific mutation and the inhibitor used.

## Discussion

Direct KRAS Switch-II pocket inhibitors have been established as a viable therapeutic strategy against a long-standing oncogenic driver, yet clinical benefit is often limited by adaptive and acquired resistance. In this study, we examined recurrent on-target resistance mutations at the Switch-II pocket and its interface with helix α3 and defined how they impair inhibitor engagement while preserving signaling competence. By combining orthogonal biochemical, structural, computational, and cellular approaches, we delineate mechanistically distinct paths to resistance, rather than a single uniform mechanism. Importantly, our data show that these resistance mutations arise within a shallow, flexible pocket formed by the mobile Switch-II region and helix α3, underscoring the inherent plasticity of the KRAS fold under therapeutic pressure. Although the resistance mutations examined here were initially identified in adagrasib resistance studies^2,4^, they represent the major recurrent Switch-II pocket hotspots observed across clinical and preclinical resistance datasets. Importantly, these mutations exhibit distinct effects across KRAS(G12C) inhibitor scaffolds. R68S and Y96 substitutions impair both adagrasib and sotorasib, whereas H95 substitutions preferentially affect adagrasib and have comparatively little effect on sotorasib^5,28,32^. Consistent with these observations, our kinetic, structural, and cellular analyses reveal both shared and inhibitor-specific resistance mechanisms, highlighting how differences in Switch-II pocket engagement influence mutational vulnerability and shape the emergence of resistance under therapeutic pressure.

Our data indicate that resistance arises primarily through two mechanistic classes that vary with the mutation and the inhibitor. The first, which we term Type I resistance, is due to altered inhibitor-pocket interactions. The second, which we term Type II resistance, is caused by stabilization of pocket-occluding protein conformations. Type I resistance results from the loss of key noncovalent contacts or the structural framework required for high-affinity engagement of the inhibitor within the Switch-II pocket and, for covalent agents, from inefficient reaction with C12. Type II resistance reflects mutations that favor Switch-II conformations that distort and partially occlude the pocket, thereby increasing the energetic barrier to drug binding, often to a prohibitive extent. These mechanisms also reflect distinct ways in which mutations reshape the conformational energy landscape of KRAS.

Y96D exemplifies a combined Type I/Type II mutation. Replacement of tyrosine with aspartate removes aromatic and packing interactions at position 96 that support inhibitor binding (Type I), while simultaneously creating pocket-occluding conformations (Type II). In the GDP-bound state, D96 forms a non-native hydrogen bond with T58 that draws Switch-II into the cavity; in the active state, it forms a salt bridge with R68, again accompanied by the inward displacement of Switch-II. In both nucleotide states, productive binding would require disrupting these stabilizing interactions, thereby increasing the energetic cost of engagement. The combined loss of favorable contacts and stabilization of pocket-occluding conformations explain the pronounced resistance conferred by Y96D across inhibitor classes, including opnurasib, which makes the least contact with Y96 but is nonetheless affected by the blocked Switch-II pocket^14^. Notably, this mutation does not destabilize the KRAS overall fold but shifts its conformational ensemble away from drug-compatible states, while preserving effector-competent configurations. The Type II resistance mechanism may also synergize with other proposed inhibition escape mechanisms, such as escape driven by increased KRAS GTP-loading^33^. Because first-generation drugs such as adagrasib and sotorasib target only the GDP-bound state, increasing the population of the GTP-bound state renders these drugs ineffective while simultaneously enhancing downstream signaling. Second-generation drugs such as BBO-8520 should be less susceptible to inhibition escape via increased KRAS-GTP because they can target the GTP-bound state as well, but they remain susceptible to the Type II mechanism of pocket occlusion regardless of which nucleotide is bound.

In contrast, Y96C, H95Q, and Q99L primarily exhibit Type I behavior. These mutations preserve the overall fold and Switch-I architecture in the GDP-bound state and primarily impair engagement by weakening pocket interactions, rather than by active occlusion. Y96C removes the aromatic side chain that mediates hydrophobic, aromatic stacking, and/or hydrogen bonding contacts with the inhibitor without introducing compensatory intramolecular interactions. H95Q induces minimal backbone rearrangement, consistent with loss of inhibitor-specific contacts at the pocket base. Q99L eliminates a polar residue and associated water-mediated stabilization at the Switch-II/helix α3 interface, subtly altering local packing while remaining structurally permissive. These substitutions show that even small changes in pocket geometry or electrostatics are sufficient to reduce engagement in a cavity that is inherently shallow and dynamic. In these cases, redistribution or loss of favorable ligand interactions reduces drug binding and efficacy, but to a lesser extent than mutations that directly occlude the pocket.

R68S displays features of both Type I and Type II, although less strongly than Y96D. R68 has broad architectural effects, helping define the Switch-II pocket boundary and coupling Switch-II to helix α3. Substitution with serine removes a key electrostatic and steric element at the pocket entrance and promotes altered dynamics that reduce the population of binding-competent pocket conformations. Thus, R68S combines Type I loss of favorable contacts with partial Type II remodeling of the pocket architecture. Unlike Y96D, however, R68S does not strongly stabilize a single occluded conformation; instead, it increases conformational heterogeneity within the Switch regions. Together, this Type I/Type II framework provides a unified model for interpreting mutation-specific resistance phenotypes and explains why distinct inhibitors exhibit differential vulnerability depending on how heavily they rely on specific pocket contacts versus conformational accessibility. Although defined using KRAS G12C resistance mutations, the Type I/Type II resistance framework is likely to apply to other KRAS G12X mutants targeted by Switch-II pocket inhibitors, although its broader applicability remains to be verified.

The graded reductions in MRTX1133 affinity (Fig. 3e) indicate progressive disruption of noncovalent Switch-II pocket engagement, which can arise from weakened inhibitor-pocket interactions (Type I) and/or reduced accessibility of a binding-competent pocket conformation (Type II). Mutations such as R68S, H95Q, Y96C, and Y96D disrupt interactions that help orient inhibitors in the binding pocket, conceivably also resulting in less efficient covalent engagement. Because our measurements of *k*_inact_/*K*_I_ do not yield individual values of *k*_inact_ and *K*_I_, we are unable to determine the precise magnitude of the relative contributions of the noncovalent and covalent binding steps. For covalent inhibitors, *k*_inact_/*K*_I_ reflects the combined efficiency of reversible binding (*K*_I_) and irreversible covalent bond formation (*k*_inact_). A decrease in *k*_inact_/*K*_I,_ therefore, signifies a lower probability of productive drug binding at a given concentration. Because C12 is unchanged and the resistance substitutions are distal to the reactive thiol, the most likely explanation for an overall reduction in *k*_inact_/*K*_I_ is disrupted formation of the pre-reaction encounter complex, effectively increasing *K*_I_ and possibly also misaligning the electrophile relative to C12, rather than altered intrinsic cysteine reactivity. This framework accounts for the mutation- and scaffold-dependent resistance phenotypes observed across inhibitors.

An important functional question is whether these resistance mutations impair effector engagement. RAF1 binding was largely preserved across the panel, consistent with the dominant contribution of Switch-I to the RAS-RAF interface. In contrast, PI3Kα binding was more sensitive to substitutions near Switch-II, which contributes directly to the RAS-PI3Kα interface, and the effects were mutation-specific: R68S showed no detectable binding, Q99L bound only weakly, and H95Q and Y96C displayed reduced affinity, whereas Y96D retained near-native binding. For mutations such as R68S and Q99L that markedly weaken PI3Kα binding, resistance may shift signaling dependence toward the MAPK pathway while reducing direct KRAS-driven PI3K-AKT activation. Notably, Y96D, despite combining strong Type I and Type II effects on Switch-II pocket inhibitors, preserves near-native binding to both RAF1 and PI3Kα and remains signaling-competent in cells. The fact that effector interactions are preserved highlights that the resistance mutations emerging in the Switch-II pocket are due to drug binding, rather than their role in KRAS-mediated downstream signaling. This capacity to lessen or abolish inhibitor binding while maintaining productive effector interactions likely contributes to the persistence of such substitutions in the clinical setting.

Cellular MAPK suppression broadly parallels the biochemical *k*_inact_/*K*_I_ measurements. Mutations that abolish or markedly reduce covalent engagement generally confer strong resistance to signaling. For example, Y96D eliminates detectable *k*_inact_/*K*_I_ for sotorasib and adagrasib and correspondingly yields essentially no pERK suppression, while R68S strongly reduces engagement by these agents and similarly essentially eliminates MAPK inhibition. In contrast, BBO-8520 retains measurable *k*_inact_/*K*_I_ across all mutants, including Y96D and R68S, and also maintains moderate pERK suppression, consistent with its higher baseline engagement efficiency. H95Q further shows consistency between biochemical and cellular results: adagrasib shows no detectable *k*_inact_/*K*_I_ and no suppression of pERK, whereas sotorasib and BBO-8520 retain measurable engagement and moderate pathway inhibition. Q99L represents a milder phenotype in which reduced but detectable *k*_inact_/*K*_I_ across all three inhibitors corresponds to partial suppression of pERK.

Importantly, the magnitude of cellular resistance does not scale directly with fold-reductions in *k*_inact_/*K*_I_. Even substantial decreases in BBO-8520 engagement permit meaningful pERK suppression, consistent with its higher baseline intrinsic engagement efficiency. In contrast, inhibitors with lower baseline *k*_inact_/*K*_I_ values, particularly adagrasib, are more sensitive to engagement defects, such that moderate impairment of drug binding can translate into near-complete loss of pERK suppression. These findings indicate that cellular MAPK output reflects not only covalent reaction efficiency but also baseline engagement strength, intracellular drug exposure, KRAS nucleotide-state distribution, and the nonlinear relationship between fractional KRAS occupancy and MAPK pathway activation.

One open clinical question is whether improved covalent engagement efficiency, reflected in higher *k*_inact_/*K*_I_ values, translates into improved clinical efficacy. Although direct comparisons across clinical trials are complicated by differences in study design and patient populations, the substantially higher *k*_inact_/*K*_I_ of BBO-8520, together with its ability to engage both GDP- and GTP-bound KRAS(G12C), is directionally consistent with the encouraging early clinical activity reported for this compound. Future clinical studies will be required to determine the extent to which enhanced covalent engagement efficiency contributes to durable improvements in patient outcomes.

The distinction between first- and second-generation inhibitors further clarifies how structural features influence susceptibility to Type I and Type II resistance mechanisms. First-generation KRAS G12C inhibitors, such as sotorasib and adagrasib, are GDP-state selective and concentrate binding energy within a relatively defined network of Switch-II pocket contacts. Their lower baseline *k*_inact_/*K*_I_ values indicate that disruption of even a single hotspot interaction (Type I) or modest distortion of pocket geometry (Type II) can substantially diminish productive engagement. In contrast, second-generation chemotypes, including dual-state inhibitors such as BBO-8520 and related compounds, are designed to engage a broader conformational and nucleotide-state landscape and exhibit higher baseline *k*_inact_/*K*_I_ values. Although resistance mutations still impair drug binding in a mutation-specific manner, compounds with greater intrinsic engagement efficiency are more likely to retain measurable activity across variants that combine Type I and Type II features. Together, these findings indicate that both intrinsic engagement strength and the capacity to accommodate conformational variability are important determinants of robustness to recurrent on-target resistance mutations.

These findings suggest several considerations for future KRAS inhibitor development in the context of Type I and Type II vulnerability. Distributing binding interactions across multiple regions of the Switch-II pocket, rather than concentrating them in one or two hotspot residues, may reduce sensitivity to single Type I substitutions. Incorporating contacts with backbone atoms or structurally constrained elements and designing ligands that tolerate modest shifts in Switch-II geometry may reduce susceptibility to Type II conformational bias. Such strategies will not eliminate resistance but may reduce its frequency by requiring combinations of mutational events that are collectively less probable than the emergence of single hotspot substitutions.

Targeting surfaces beyond the canonical Switch-II pocket provides an additional means of diversifying selective pressure arising during therapy. Indirect tricomplex inhibitors that engage KRAS together with cyclophilin A demonstrate that composite interfaces can lessen or bypass resistance resulting from many Switch-II-specific mutations characterized here, including the most severe mutations such as Y96D^5,23^, although they suffer from their own resistance liabilities, such as substitutions at Y64^22^. These observations underscore that resistance is interface-specific rather than universal. Development of inhibitors directed at additional allosteric surfaces or transient conformations would expand therapeutic options and create opportunities for rational sequencing of combinations of drugs with mechanistically distinct inhibitory mechanisms.

Durable suppression of KRAS-driven tumors will likely require integrating optimized KRAS inhibitors with pathway-level interventions. Clinical strategies already combine KRAS G12C inhibitors with receptor tyrosine kinase inhibitors^34–36^, or SHP2 inhibitors^37^ to reduce levels of KRAS G12C in the GTP-bound state, MEK inhibitors^38–40^ to blunt adaptive MAPK reactivation, or immune checkpoint blockade^41^. Additional strategies under evaluation include vertical inhibition of the RAS-MAPK cascade, co-targeting PI3K-AKT signaling where retained, and rational sequencing of mechanistically distinct KRAS inhibitors to modulate selective pressure^19,20,38,42,43^. By simultaneously constraining adaptive signaling and reducing the fitness advantage of individual resistance alleles, such approaches may delay or redirect resistance trajectories.

In summary, this work defines mechanisms of resistance at the Switch-II pocket and highlights how different substitutions differentially combine Type I and Type II mechanisms while preserving oncogenic signaling. Anticipating these mechanisms at both the level of inhibitor design and the therapeutic context will be critical for extending the durability of KRAS-targeted therapies.

## Methods

### Cloning, expression, and purification of recombinant proteins

The entry clone encoding KRAS4b(1–169)-G12C/C118S was generated using the QuikChange II Site-Directed Mutagenesis Kit (Agilent), and the KRAS4b(1–188)-G12C entry clone was generated by PCR (GenBank NM_004985.4). Entry clones encoding the KRAS4b(1–169)-G12C resistance variants (R68S, H95Q, Y96C, Y96D, and Q99L), all in the C118S background, and the KRAS4b(1–188)-G12C resistance variants (R68S, H95Q, Y96C, Y96D, and Q99L) were generated by ATUM via gene synthesis and optimized for expression in bacterial and mammalian cells, respectively. All bacterial entry clones incorporated an upstream tobacco etch virus (TEV) protease cleavage site (ENLYFQ/G) and were subcloned by Gateway LR recombination into pDest-566 (Addgene #11517) to generate final expression constructs encoding proteins fused to an N-terminal His₆-maltose-binding protein (MBP) tag^44^. Mammalian multisite expression constructs were assembled by Gateway multisite recombination using an entry clone containing the CMV51 promoter sequence, generated by PCR from pDest-720 (Sabine Geisse, Novartis), and the destination vector pDest-303 (Addgene #159678). Plasmid DNA was prepared using the Plasmid Plus Maxi Kit (QIAGEN). RAF1 RBD (52–131), RAF1 RBD-CRD (52–188), and PI3Kα RBD (157–300) constructs were generated and expressed using previously established bacterial expression systems^30,45^.

KRAS G12C and resistance mutants were expressed in *E. coli* BL21 (DE3) STAR (Thermo Fisher Scientific) using the Dynamite medium protocol, a modified Studier auto-induction system that supports high-density growth and efficient overnight expression following IPTG induction at 16 °C^46^. Proteins were purified using a multistep workflow. Clarified bacterial lysates were subjected to immobilized metal affinity chromatography (IMAC) to capture His₆-MBP-tagged proteins, followed by removal of the affinity tag via cleavage with His7-tagged TEV protease^47^. Untagged target proteins were recovered in the IMAC flowthrough, and final polishing was performed by size-exclusion chromatography in buffer containing 20 mM HEPES (pH 7.3), 150 mM NaCl, and 2 mM MgCl₂^48^. Protein purity and integrity were assessed by SDS-PAGE, thermal melting by DSF, and mass spectrometry^49^. Uniformly ^15^N-labeled KRAS4b(1-169)-G12C/C118S and KRAS4b(1-169)-G12C/Y96D/C118S were expressed in *E. coli* BL21 (DE3) STAR grown in M9 minimal medium supplemented with ^15^NH_4_Cl as the sole nitrogen source^50^. The labeled proteins were purified using the same workflow as the corresponding unlabeled constructs. For ITC measurements and co-crystallization with the RAF1 RBD-CRD, GDP-loaded KRAS proteins were first exchanged to the non-hydrolyzable GTP analog GMPPNP^51,52^. The extent and efficiency of nucleotide exchange were evaluated by ion-pairing HPLC^53^.

### Mass spectrometry-based kinetic analysis of covalent inhibitor engagement

Covalent modification of KRAS G12C and resistance mutants by covalent Switch-II pocket inhibitors was quantified using a MALDI-TOF intact protein mass spectrometry-based assay^27^. All KRAS proteins were analyzed in the C118S background and were predominantly GDP-bound at the start of the experiments. The relative populations of GDP- and GTP-bound states were assessed prior to analysis and incorporated into the final kinetic calculations. Unless otherwise indicated, experiments were performed at a protein concentration of 0.1 µM in 20 mM HEPES (pH 7.3), 150 mM NaCl, and 2 mM MgCl₂. KRAS G12C and the resistance mutants G12C/R68S, G12C/H95Q, G12C/Y96D, and G12C/Q99L were incubated with varying molar ratios of covalent inhibitors (BBO-8520, sotorasib, or adagrasib), as specified for each experiment. Independent measurements were performed at different compound-to-protein ratios for each KRAS variant–inhibitor combination. For BBO-8520, the number of measurements was *n* = 7 (G12C), 3 (G12C/R68S), 6 (G12C/H95Q), 7 (G12C/Y96D), and 5 (G12C/Q99L). For sotorasib, *n* = 7 (G12C), 5 (G12C/R68S), 7 (G12C/H95Q), 7 (G12C/Y96D), and 7 (G12C/Q99L). For adagrasib, *n* = 4 (G12C), 7 (G12C/R68S), 7 (G12C/H95Q), 7 (G12C/Y96D), and 7 (G12C/Q99L). Each measurement was performed at a different compound-to-protein ratio and therefore represents a separate kinetic measurement rather than a technical replicate. For KRAS G12C treated with BBO-8520, reactions were performed at a reduced protein concentration of 0.025 µM due to the high reaction rates observed even at the lowest compound-to-protein ratios.

Reactions were initiated by adding the inhibitor and allowed to proceed at room temperature. At defined time points, 60 µL aliquots were removed and quenched by the addition of 90 µL of 100 mM HCl. Samples were desalted and concentrated using ZipTip pipette tips and eluted with 4 µL of 60% acetonitrile and 0.4% trifluoroacetic acid (TFA). The eluted protein was mixed with a MALDI matrix consisting of saturated sinapinic acid prepared in 1:1 acetonitrile/water with 0.15% TFA, and spectra were acquired by MALDI-TOF mass spectrometry. MALDI-TOF measurements were performed on a Bruker Daltonics rapifleX Tissuetyper MALDI-TOF/TOF mass spectrometer in linear mode over a mass range of 18.6–21.6 kDa. No liquid or gas chromatography separation was performed. Spectra were processed using Bruker flexAnalysis (version 4.0, Build 14). Spectra were smoothed using the Savitzky–Golay algorithm, baseline-subtracted using the median algorithm, and subjected to centroid peak detection.

The extent of covalent modification was quantified by monitoring the relative signal intensities of unmodified and covalently modified protein species over time. Protein species were assigned based on the observed intact masses of unmodified KRAS and the corresponding inhibitor-modified KRAS species. Percent modification was calculated from peak heights as the signal corresponding to covalently modified protein divided by the sum of the signals corresponding to unmodified and covalently modified protein. We used peak heights, rather than peak areas, for quantification. Time-course data collected at multiple compound-to-protein ratios were used to derive *k*_inact_/*K*_I_^16,54^. Data were analyzed using GraphPad Prism. Individual *k*_inact_/*K*_I_ values obtained from the independent measurements are shown in Fig. 2d–f and provided in the Source Data file.

### Measurement of protein melting temperature by differential scanning fluorimetry

All DSF experiments to measure protein melting temperature (*T*_m_) in the absence and presence of the noncovalent inhibitor MRTX1133 were carried out using an Applied Biosystems QuantStudio 3 RT-PCR instrument. Data were recorded continuously while ramping the temperature from 25 °C to 98 °C at a rate of 0.05 °C/s. The optical filter pair x2-m2 was used for data collection. The total volume of all samples was 20 µL, consisting of 7.5 µM protein in a buffer of 20 mM HEPES (pH 7.4), 150 mM NaCl, 5 mM MgCl_2_, 1 mM TCEP, 5% v/v DMSO, and either 0 or 250 µM MRTX1133. At 250 µM MRTX1133, the fraction of protein with bound inhibitor should be 90% for the Y96D mutant and ~99% for all other mutants. In all samples, SYPRO Orange dye was used at a concentration of 5×, diluted from the manufacturer’s stock concentration of 5000×. For each KRAS mutant, three technical replicate measurements were performed. The melting temperature was extracted from the measured data using the first-derivative method in the Applied Biosystems Protein Thermal Shift software version 1.3. For each mutant, the statistical significance of the difference in melting temperature measured at 0 and 250 µM MRTX1133 was determined using a one-tailed Welch’s t-test.

### Binding affinity measurements using isothermal titration calorimetry

All ITC experiments were performed at 25 °C using a MicroCal PEAQ-ITC instrument with a stirring rate of 750 rpm and the feedback mode set to high. All KRAS proteins used in ITC experiments (G12C, G12C/R68S, G12C/H95Q, G12C/Y96C, G12C/Y96D, and G12C/Q99L) contained the C118S mutation. For the experiments measuring binding to MRTX1133 or the RAF1 RBD, all proteins were dialyzed overnight at 4 °C against 20 mM HEPES (pH 7.4), 150 mM NaCl, 5 mM MgCl_2_, and 1 mM TCEP; for measuring binding to the PI3Kα RBD, the dialysis buffer formulation was the same except for using 50 mM NaCl instead of 150 mM. All samples were degassed for ~8 min before being loaded into the calorimeter.

The binding affinity of the noncovalent inhibitor MRTX1133 to the G12C control and resistance mutants was measured with KRAS in the GDP-bound state. After overnight dialysis, the dialysis buffer was used to dilute MRTX1133 in 100% DMSO to a final concentration of 423 µM in 5% v/v DMSO, and the composition of the dialyzed protein samples was also adjusted to contain 5% v/v DMSO. ITC experiments were performed using 19 total injections with a spacing of 180 s between injections and a KRAS-GDP concentration of 30–50 µM in the cell. Binding measurements of the GMPPNP-bound KRAS mutants to either the RAF1 or PI3Kα RBD were performed similarly, using 30–50 µM KRAS in the cell and 300–700 µM RAF1 RBD or PI3Kα RBD in the syringe.

For all datasets, raw thermograms were integrated using NITPIC^55^, and binding parameters were extracted by simultaneous global fitting^56^ of two independent measurements performed at different [syringe]:[cell] ratios to a 1:1 binding model using the program SEDPHAT^57^. Thermograms and integrated data were plotted using either GraphPad Prism version 10.6.1 (GraphPad Software, LLC) or GUSSI^58^ version 2.1.6.

### Crystallization and X-ray data collection

For all proteins, crystallization was carried out by vapor diffusion using the sitting-drop method, mixing equal volumes of protein and crystallization solution and incubating the drops at either 20 °C or 4 °C. Crystallization of G12C/Y96D-GDP proved challenging, with no crystals obtained after extensive screening using commercially available kits at both temperatures. Crystals were ultimately obtained from a single condition in the non-commercial Sweet16 screen^59^. Comprehensive crystallization and incubation conditions are given in Supplementary Table 1. Crystals of the G12C/Y96D-GDP, G12C/R68S-GDP, and G12C/H95Q-GDP resistance mutants were harvested and vitrified in liquid nitrogen without additional cryoprotectant. Diffraction data for these crystals were collected at the FMX beamline^60^ of the National Synchrotron Light Source II, Brookhaven National Laboratory (Upton, NY, USA), using X-rays of wavelength 0.9793 Å and a Dectris EIGER 16M detector. Crystals of the G12C/Y96C-GDP and G12C/Q99L-GDP resistance mutants were similarly harvested and vitrified in liquid nitrogen without additional cryoprotectant. Diffraction data for these crystals were collected at beamline 12-1 of the Stanford Synchrotron Radiation Lightsource (Menlo Park, CA, USA) using X-rays of wavelength 0.9795 Å and a Dectris EIGER2 X 16M detector. Crystals of the G12C/Y96D-GMPPNP complex with the RAF1 RBD-CRD were harvested and vitrified in liquid nitrogen without additional cryoprotection. Diffraction data for this complex were acquired at NECAT beamline 24-ID-E of the Advanced Photon Source (Lemont, IL, USA) using X-rays of wavelength 0.9791 Å and a Dectris EIGER 16M detector.

### Structure solution and analysis

For all datasets, data reduction and scaling were carried out using XDS^61^. Phasing was accomplished via molecular replacement using either EPMR^62^ or the MoRDa pipeline^63^. For the G12C/Y96D, G12C/R68S, and G12C/Q99L mutants, the best molecular replacement solutions were obtained using PDB entry 4EPV as the search model. For the G12C/Y96C and G12C/H95Q mutants, using PDB entry 2QUZ as the search model yielded the optimal MR solutions. For the G12C/Y96D-GMPPNP complex with the RAF1 RBD-CRD, molecular replacement was performed using chain B of PDB entry 8T75 as the search model for the RAF1 RBD-CRD and PDB entry 1CTQ for KRAS G12C/Y96D bound to GMPPNP. Molecular replacement solutions were rebuilt using either ARP/wARP^64^ or the ModelCraft pipeline^65^. Structures were then subjected to iterative cycles of manual model building in Coot^66^ and automated refinement using phenix.refine version 1.21.2^67^. Comprehensive data collection and refinement statistics are presented in Table 1. All protein structural figures were prepared using PyMOL version 3.1 (Schrödinger Inc.). Crystallographic and structural analysis software support is provided by the SBGrid consortium^68^.

**Table 1:** Crystallographic data collection and refinement statistics

	KRAS G12C/R68S-GDP	KRAS G12C/H95Q-GDP	KRAS G12C/Y96C-GDP	KRAS G12C/Y96D-GDP	KRAS G12C/Q99L-GDP	KRAS G12C/Y96D-GMPPNP + RAF1 RBD-CRD
**Data collection**						
Wavelength (Å)	0.9793	0.9793	0.9795	0.9793	0.9795	0.9791
Resolution range (Å)	31.83–1.65 (1.68–1.65)^a^	30.83–1.35 (1.38–1.35)	30.18–1.5 (1.59–1.5)	42.99–1.5 (1.55–1.5)	28.19–1.16 (1.18–1.16)	48.61–2.5 (2.63–2.5)
Space group	P 1 21 1	H 3 2	H 3 2	P 3 2 1	P 21 21 21	P 6 2 2
Unit cell a, b, c (Å) α, β, γ (°)	42.84, 103.06, 56.67 90, 109.12, 90	94.23 94.23, 122.32 90, 90, 120	92.84, 92.84, 119.19 90, 90, 120	77.70, 77.70, 55.87 90, 90, 120	39.21, 40.57, 92.16 90, 90, 90	133.42, 133.42, 89.93 90, 90, 120
Total reflections	362241 (16995)	910697 (50355)	474717 (52881)	623599 (46385)	535643 (11090)	586317 (84247)
Unique reflections	55731 (2717)	45930 (2849)	31544 (5082)	31542 (2821)	49307 (1762)	16875 (2356)
Multiplicity	6.5 (6.3)	19.8 (17.7)	14.9 (10.4)	19.8 (16.4)	10.9 (6.3)	34.7 (35.8)
Completeness (%)	99.8 (97.9)	99.7 (98.8)	99.2 (95.7)	99.8 (99.5)	95.3 (62.2)	98.6 (96.0)
Mean I/sigma(I)	9.7 (1.5)	13.5 (0.63)	16.5 (1.6)	15.4 (0.94)	18.5 (4.2)	12.2 (1.2)
Wilson B-factor (Å²)	20.5	20.6	17.2	21.9	12.0	63.0
R-merge	0.115 (1.174)	0.094 (3.411)	0.102 (1.50)	0.112 (2.68)	0.065 (0.410)	0.232 (1.933)
R-meas	0.125 (1.289)	0.097 (3.512)	0.107 (1.68)	0.115 (2.77)	0.068 (0.447)	0.235 (1.953)
R-pim	0.049 (0.502)	0.022 (0.828)	0.037 (0.743)	0.026 (0.676)	0.020 (0.1756)	0.040 (0.324)
CC1/2	0.998 (0.433)	1 (0.322)	0.999 (0.546)	1 (0.307)	0.999 (0.941)	0.999 (0.82)
CC*	0.999 (0.777)	1 (0.698)	1 (0.84)	1 (0.685)	1 (0.985)	1 (0.949)
**Refinement**						
Reflections used in refinement	55706 (2713)	45814 (2819)	31529 (2671)	31478 (2806)	49303 (1761)	16637 (2264)
Reflections used for R-free	2786 (136)	2292 (141)	1578 (134)	1576 (141)	2465 (88)	1000 (135)
R-work	0.181 (0.305)	0.185 (0.458)	0.175 (0.312)	0.187 (0.352)	0.144 (0.142)	0.210 (0.408)
R-free	0.226 (0.317)	0.213 (0.489)	0.199 (0.344)	0.223 (0.350)	0.174 (0.179)	0.259 (0.509)
Number of non-hydrogen atoms	4210	1534	1573	1550	1615	2511
macromolecules	3785	1353	1339	1358	1386	2406
ligands	90	29	37	28	29	35
solvent	335	152	197	164	200	70
Protein residues	476	165	166	166	170	301
RMS bonds (Å)	0.009	0.011	0.008	0.006	0.012	0.003
RMS angles (°)	1.11	1.29	1.01	0.92	1.36	0.47
Ramachandran favored (%)	98.0	96.3	97.5	97.6	98.8	96.2
Ramachandran allowed (%)	2.0	3.7	2.5	2.4	1.2	3.8
Ramachandran outliers (%)	0	0	0	0	0	0
Rotamer outliers (%)	0	0	0.66	0	0	1.12
Clashscore	1.95	5.47	1.46	1.82	3.57	3.11
Average B-factor (Å²)	31.6	36.0	26.4	30.6	17.0	73.7
macromolecules (Å²)	31.4	34.9	25.1	29.6	15.6	74.1
ligands (Å²)	25.5	27.8	21.8	23.3	11.3	70.8
solvent (Å²)	36.2	46.4	36.3	39.6	27.3	61.2

^a^Values in parentheses refer to the highest resolution shell.

### Analytical size-exclusion chromatography

To assess stable complex formation, the RAF1 RBD at ~200 µM was mixed with each of the five GMPPNP-bound resistance mutants (G12C/R68S, G12C/H95Q, G12C/Y96C, G12C/Y96D, and G12C/Q99L) in a molar ratio of 1.1: 1. Because of the decreased solubility of the G12C/Y96D mutant, the concentrations of RAF1 RBD and G12C/Y96D used were approximately half that of the other solutions, although the 1.1:1 molar ratio was maintained. After incubation for 2 h at 4 °C, 200 µL of each mixture was passed over an S75 Increase 10/300 GL size-exclusion column at a flow rate of 0.5 mL/min. For all RAF1 RBD: resistance mutant mixtures, a single elution peak was observed at an earlier elution time than for the RAF1 RBD control alone. Collected fractions were analyzed by SDS-PAGE and Coomassie staining.

### Molecular dynamics simulations

AMBER24^69^ and AMBERTools 25^70^ were used for simulations of the G12C-GDP control structure, all five G12C resistance mutants in the GDP-bound state, and G12C/Y96D-GTP. AMBER’s *tleap* program was used to prepare all proteins for simulation. For each protein, the size of the model was 169 protein residues, either one GDP or GTP nucleotide, and one Mg^2+^ cofactor, solvated in an OPC periodic solvent box of size roughly 71 × 75 × 73 Å^3^ containing ~9000 waters; all protein atoms were at least 10 Å from the box boundary. The SLTCAP web server^71^ was used to add the appropriate number of sodium and chloride ions to bring the overall ionic concentration to 150 mM. The protein was parameterized using the ff19SB forcefield. The protonation state of histidine residues and Asn/Gln “flips” were determined using *reduce*^72^. *Antechamber* was used to parameterize both GDP and GTP nucleotides by adding AM1-BCC charges^73^ and to apply the GAFF2 force field^74^. The module *PMEMD.cuda*^75^ was used to carry out simulations using P100 GPUs.

All simulations began with a multi-step equilibration protocol. The equilibration run consisted of 4 minimizations of up to 40,000 steps each using progressively reduced restraints, followed by six 20-ps runs at constant volume during which the simulation temperature was raised from 0 to 298.15 K. Langevin dynamics^76^ were used to maintain the temperature of the simulation with a collision frequency of 2 ps^−1^. Next, a constant pressure (NPT) run allowed the volume of the box to adjust for 10.2 ns to maintain 1 bar of pressure, with the heavy atoms of the protein backbone restrained. Finally, constant-volume (NVT) simulations were performed for 10 ns under the same conditions as the subsequent production simulations. Production NVT simulations were performed for 1 µs. For all systems, equilibration and production were run in five replicas, totaling 5 µs of production simulations. The Shake algorithm^77^ was used with a 2-fs time step. Periodic boundary conditions were applied, and the Particle Mesh Ewald method^78^ was used to calculate long-range electrostatics. Simulations were analyzed to determine the proteins’ conformational fluctuations, structural dynamics, and inter-residue H-bonding interactions (see Supplementary Methods). Residues not resolved in the crystal structures were added before simulation using the *RosettaRemodel* module from the Rosetta molecular modeling suite^79^, source release #371. UCSF Chimera 1.169^80^ was used to modify GMPPNP to GTP in the structure of KRAS4b-G12C/Y96D/C118S-GNP, which was extracted from the complex with the RAF1 RBD-CRD. For all systems simulated, an RMSD plot for each individual replica simulation performed is shown in Supplementary Fig. 13.

### Nuclear magnetic resonance spectroscopy

^1^H-^15^N HSQC spectra of KRAS4b(1-169)-G12C/C118S-GDP and KRAS4b(1-169)-G12C/Y96D/C118S-GDP were recorded at 25 °C on a 700 MHz (16.4 T) Bruker Avance NEO spectrometer equipped with a helium-cooled TCI cryoprobe. The sample concentrations were 220 and 115 µM for G12C/C118S-GDP and G12C/Y96D/C118S-GDP, respectively. Before acquiring the spectra, both proteins were dialyzed overnight at 4 °C against a buffer of 20 mM HEPES (pH 7.4), 150 mM NaCl, 5 mM MgCl_2_, and 1 mM TCEP. Previously published^81^ resonance assignments for KRAS4b-G12C-GDP were obtained from the BioMagResBank^82^ under accession code 27646. Assignment mapping, spectral overlays, and chemical shift perturbation analyses were performed using the POKY^83^ software.

### Cell-based ERK and AKT phosphorylation assays

HEK293 cells were seeded in 6-well plates at 10⁵ cells per well and transiently transfected 24 h later with expression constructs encoding KRAS4b G12C or one of the G12C-based resistance variants (R68S, H95Q, Y96C, Y96D, or Q99L) using FuGENE transfection reagent. For each well, 0.8 µg of plasmid DNA was used. Cells were incubated for an additional 48 h to allow protein expression. Cells were treated for 2 h with either 0.2% DMSO or covalent KRAS G12C inhibitors. BBO-8520 was applied at final concentrations of 10 or 100 nM, whereas sotorasib and adagrasib were applied at 100 or 1000 nM. Following treatment, cells were washed twice with PBS and lysed in RBD lysis buffer. Lysates were clarified by centrifugation at 20,000 × *g* for 15 min at 4 °C, and protein concentrations were determined using a bicinchoninic acid (BCA) assay. Equal amounts of total protein were resolved by SDS-PAGE and transferred to membranes for immunoblot analysis. Membranes were probed with antibodies against phosphorylated ERK1/2 (pERK1/2), total ERK1/2, phosphorylated AKT (pAKT), total AKT, KRAS, and vinculin as a loading control. Primary antibodies were probed with corresponding fluorescent dye-tagged secondary antibodies, and images were captured by a LI-COR Odyssey M imaging system. Phosphorylated ERK1/2 or AKT (S473) band intensities were quantified using LI-COR Image Studio software analysis tools and normalized to the corresponding DMSO-treated control for each KRAS variant; for each drug and resistance variant combination, the mean and standard deviation of the normalized values from three biological replicates are plotted for pERK in Fig. 7 and for pAKT in Supplementary Fig. 12. Total ERK1/2, total AKT, KRAS expression, and vinculin levels were used to confirm comparable protein loading and expression across samples. Uncropped blots with labeled molecular weight markers are provided in the Source Data file.

### Reporting summary

Further information on research design is available in the Nature Portfolio Reporting Summary linked to this article.

## Supplementary information

Supplementary Information

Reporting Summary

Transparent Peer Review File

## Source data

Source Data

## Data Availability

Atomic coordinates and structure factors for structures determined in this study have been deposited in the Protein Data Bank (PDB) under accession codes: 11FU https://doi.org/10.2210/pdb11FU/pdb (KRAS G12C/R68S-GDP), 11ES https://doi.org/10.2210/pdb11ES/pdb (KRAS G12C/H95Q-GDP), 11FB https://doi.org/10.2210/pdb11FB/pdb (KRAS G12C/Y96C-GDP), 11FL https://doi.org/10.2210/pdb11FL/pdb (KRAS G12C/Y96D-GDP), 11GH https://doi.org/10.2210/pdb11GH/pdb (KRAS G12C/Y96D-GMPPNP in complex with RAF1 RBD-CRD), and 11FT https://doi.org/10.2210/pdb11FT/pdb (KRAS G12C/Q99L-GDP). Previously deposited structures used for analysis, comparison, or superposition include 4LDJ https://doi.org/10.2210/pdb4LDJ/pdb (KRAS G12C-GDP), 6OIM https://doi.org/10.2210/pdb6OIM/pdb (KRAS G12C-GDP bound to sotorasib), 6UT0 https://doi.org/10.2210/pdb6UT0/pdb (KRAS G12C-GDP bound to adagrasib), 9DMM https://doi.org/10.2210/pdb9DMM/pdb (KRAS G12C-GDP bound to divarasib), 7R0M https://doi.org/10.2210/pdb7R0M/pdb (KRAS G12C-GDP bound to opnurasib), 9KPN https://doi.org/10.2210/pdb9KPN/pdb (KRAS G12C-GDP bound to glecirasib), 8S8C https://doi.org/10.2210/pdb8S8C/pdb (KRAS G12C-GDP bound to calderasib), 8AFB https://doi.org/10.2210/pdb8AFB/pdb (KRAS G12C-GDP bound to BI-0474), 8V3A https://doi.org/10.2210/pdb8V3A/pdb (KRAS G12C-GDP bound to BBO-8520), and 8AZX https://doi.org/10.2210/pdb8AZX/pdb (KRAS G12C-GDP bound to BI-2865). Other structures include 8V39 https://doi.org/10.2210/pdb8V39/pdb (KRAS G12C-GMPPNP bound to BBO-8520), 7RPZ https://doi.org/10.2210/pdb7RPZ/pdb (KRAS G12D-GDP bound to MRTX1133), 9P44 https://doi.org/10.2210/pdb9P44/pdb (KRAS G12D-GDP bound to BBO-11818), 6VJJ https://doi.org/10.2210/pdb6VJJ/pdb (KRAS-RAF1 RBD), 6XHB https://doi.org/10.2210/pdb6XHB/pdb (KRAS-RAF1 RBD-CRD), and 9C15 https://doi.org/10.2210/pdb9C15/pdb (KRAS-PI3Kα). Additional data supporting the findings of this study are available from the corresponding author. Source Data are provided with this paper.
