# Supplementary material for: Structural mechanisms underlying resistance to Switch-II pocket inhibitors in KRAS
Source: Nat Commun. 2026 Sep 29;17:11229. doi: 10.1038/s41467-026-78133-4 (PMC13720030; doi:10.1038/s41467-026-78133-4)
Supplement: Supplementary file 1 [file 41467_2026_78133_MOESM1_ESM.pdf]

Structural mechanisms underlying resistance to Switch-II pocket inhibitors in KRAS

Whitley, M.J., *et al*.

Supplementary Materials

Supplementary Figures 1-13

Supplementary Tables 1 and 2

Supplementary Methods

Supplementary Figure 1. **Representative chemical scaffolds targeting the KRAS Switch-II pocket.** A central core projects substituents to G12X (warhead toward C12), to C-terminal end of Switch-II (toward R68 and Q99), and to solvent, illustrating directional engagement within the pocket. Chemical structures of covalent KRAS G12C and noncovalent KRAS G12D and pan-KRAS inhibitors are shown, with atoms engaging **R68**, **H95**, **Y96**, and **Q99** color-coded (orange, blue, red, and green) to highlight scaffold-specific interaction networks.

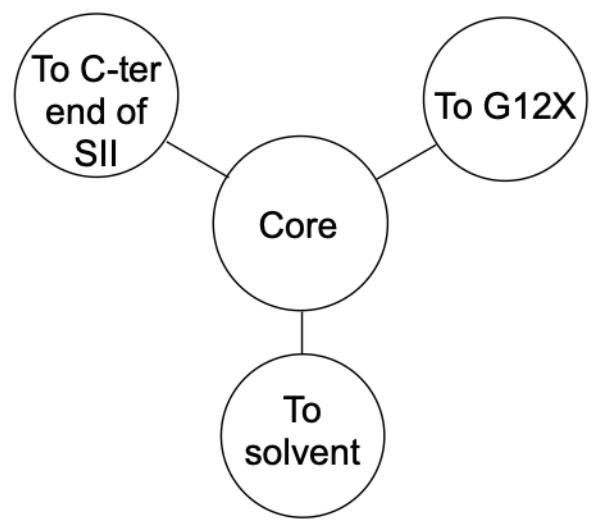

General structure

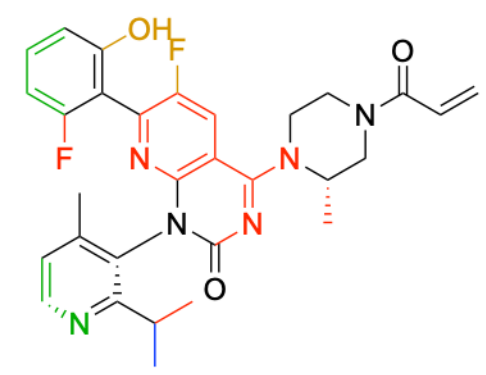
Sotorasib

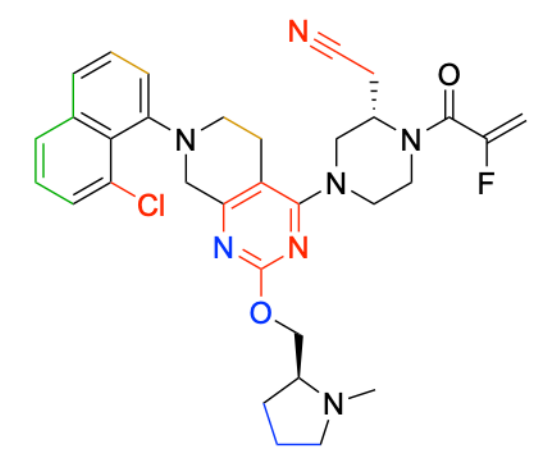
Adagrasib

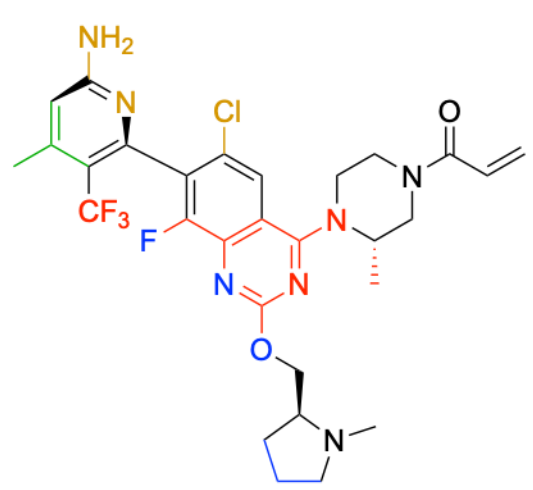
Divarasib

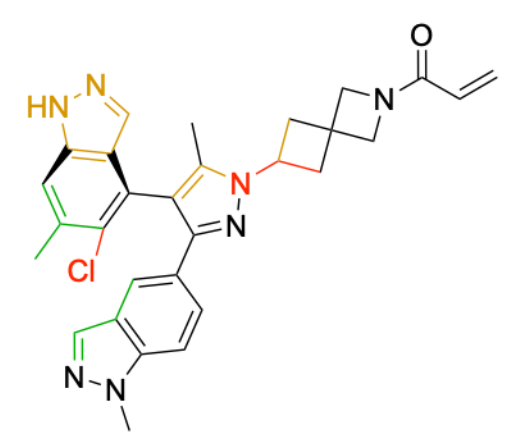
Opnurasib

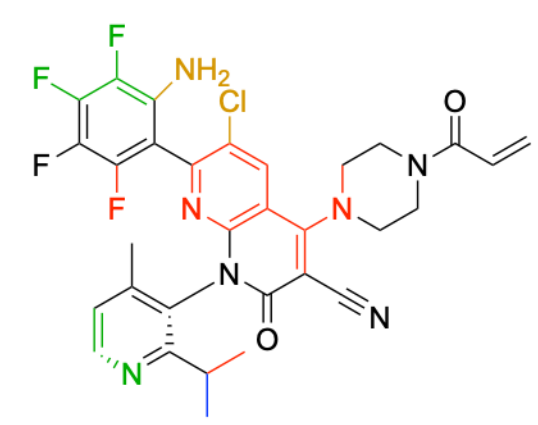
Glecirasib

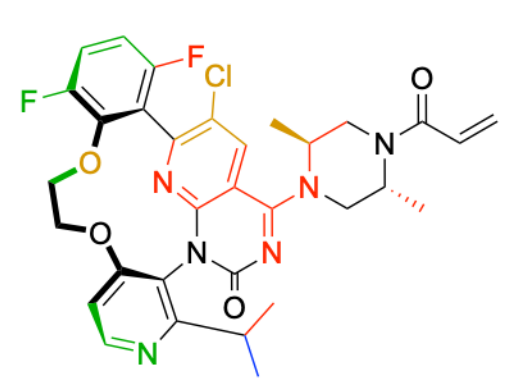
Calderasib

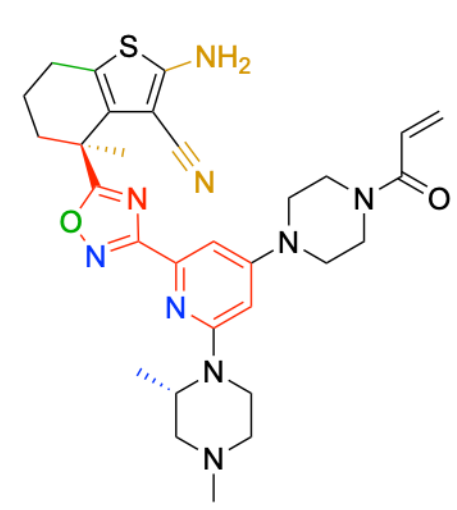
BI-0474

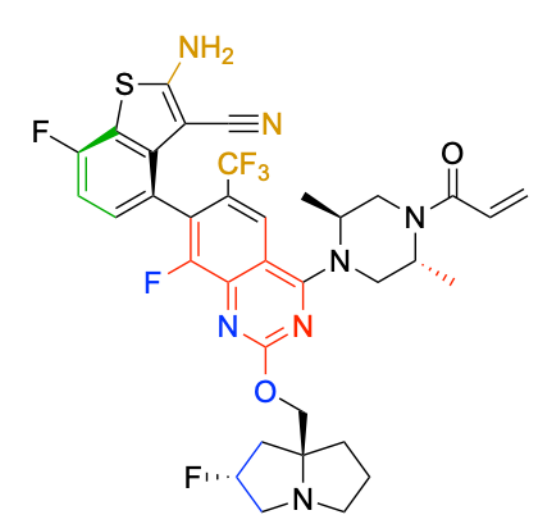
BBO-8520

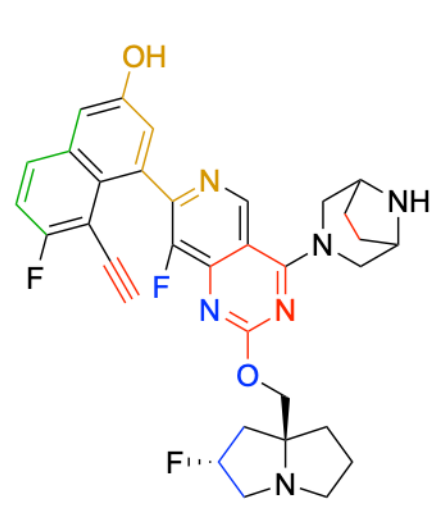
MRTX1133

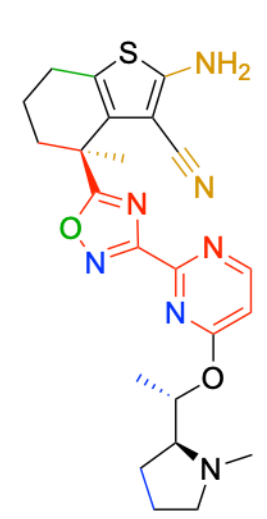
BI-2865

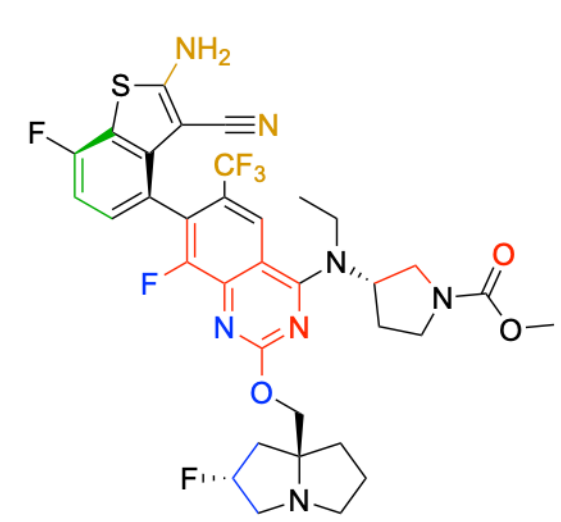
BBO-11818

Supplementary Figure 2. Differential scanning fluorimetry (DSF) analysis of KRAS G12C and resistance mutants. DSF melting curves for KRAS G12C and the five resistance mutants measured in the absence (black; three technical replicates) and presence of 250 µM MRTX1133 (grey; three technical replicates). Fluorescence on the y-axis is measured in arbitrary units. A one-tailed Welch's t-test has been used to check the significance of the T_m differences.

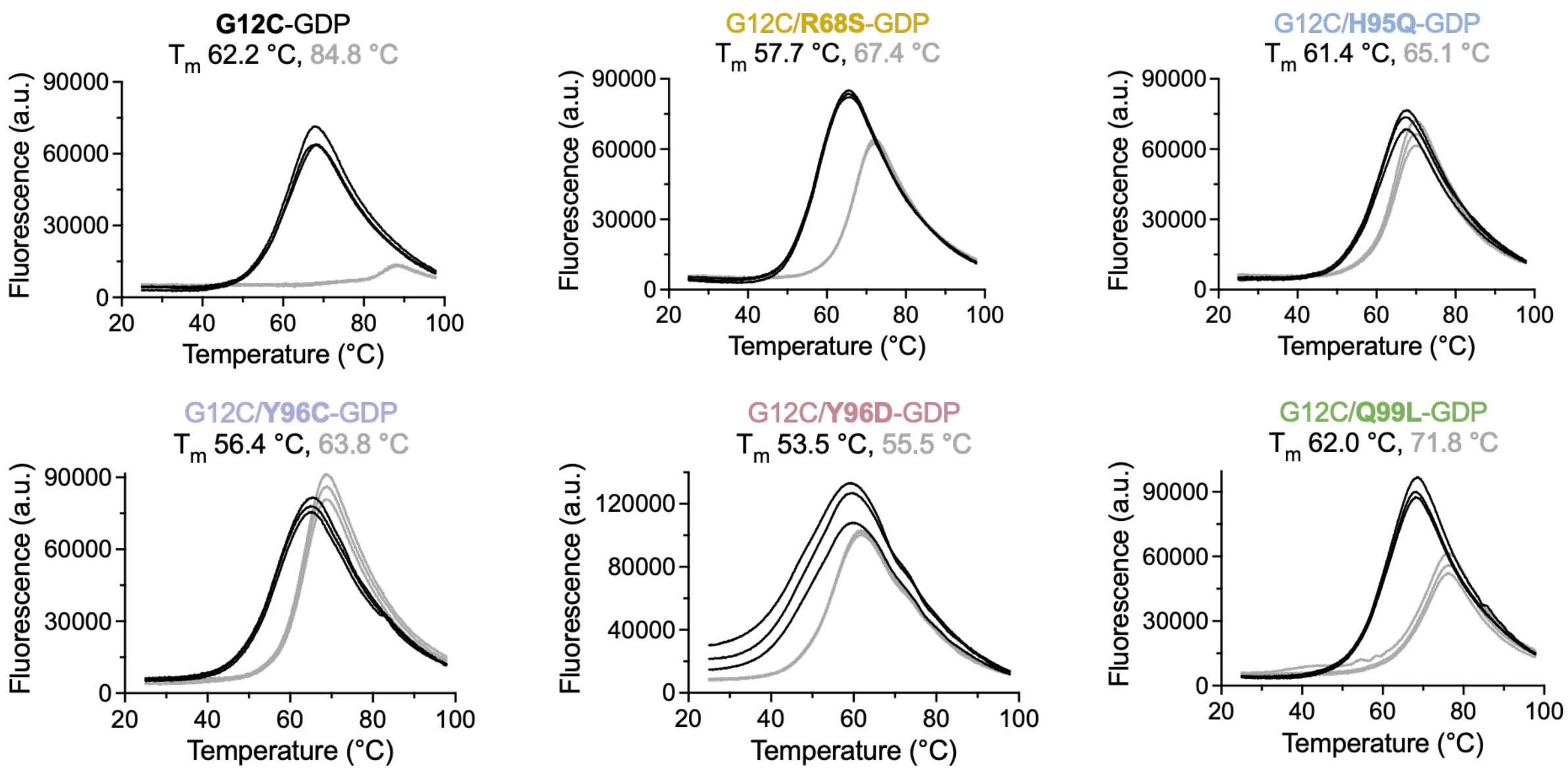

Descriptive statistics for each mutant.
SD = standard deviation, SE = standard error

	Group	N	Mean	SD	SE
G12C	0 µM MRTX1133	3	62.23416	0.30390	0.17546
	250 µM MRTX1133	3	84.76447	0.08286	0.04784
G12C/R68S	0 µM MRTX1133	3	57.70764	0.14646	0.08456
	250 µM MRTX1133	3	67.39005	0.08333	0.04811
G12C/H95Q	0 µM MRTX1133	3	61.40673	0.31330	0.18088
	250 µM MRTX1133	3	65.10266	0.17042	0.09839
G12C/Y96C	0 µM MRTX1133	3	56.39351	0.14642	0.08454
	250 µM MRTX1133	3	63.78862	0.08255	0.04766
G12C/Y96D	0 µM MRTX1133	3	53.52014	1.30121	0.75125
	250 µM MRTX1133	3	55.51508	0.14490	0.08366
G12C/Q99L	0 µM MRTX1133	3	61.99073	0.07842	0.04528
	250 µM MRTX1133	3	71.77016	0.08650	0.04994

One-tailed Welch's t-test for each mutant.
Null hypothesis: The mean T_m of the 0 µM MRTX1133 group is identical to the mean T_m of the 250 µM MRTX1133 group.
Alternative hypothesis: The mean T_m of the 0 µM MRTX1133 group is less than the mean T_m of the 250 µM MRTX1133 group.
α = 0.05
t = test statistic, df = degrees of freedom, p = p-value

	t	df	p
G12C	−123.88499	2.29574	< .00001
G12C/R68S	−99.52096	3.17206	< .00001
G12C/H95Q	−17.94910	3.08827	.00016
G12C/Y96C	−76.20201	3.15464	< .00001
G12C/Y96D	−2.63918	2.04959	.05781
G12C/Q99L	−145.07412	3.96213	< .00001

All T_m differences are statistically significant except for G12C/Y96D, which just misses the significance threshold using α = 0.05.

Supplementary Figure 3. Isothermal titration calorimetry (ITC) measurement of MRTX1133 binding to GDP-bound KRAS G12C and resistance mutants. Raw thermograms (top panels) and corresponding integrated heat plots (bottom panels) are shown for two independent experiments; the syringe and cell concentrations used in each experiment are given. The binding affinity was extracted by simultaneous global fitting of the two independent experiments (see Fig. 3e).

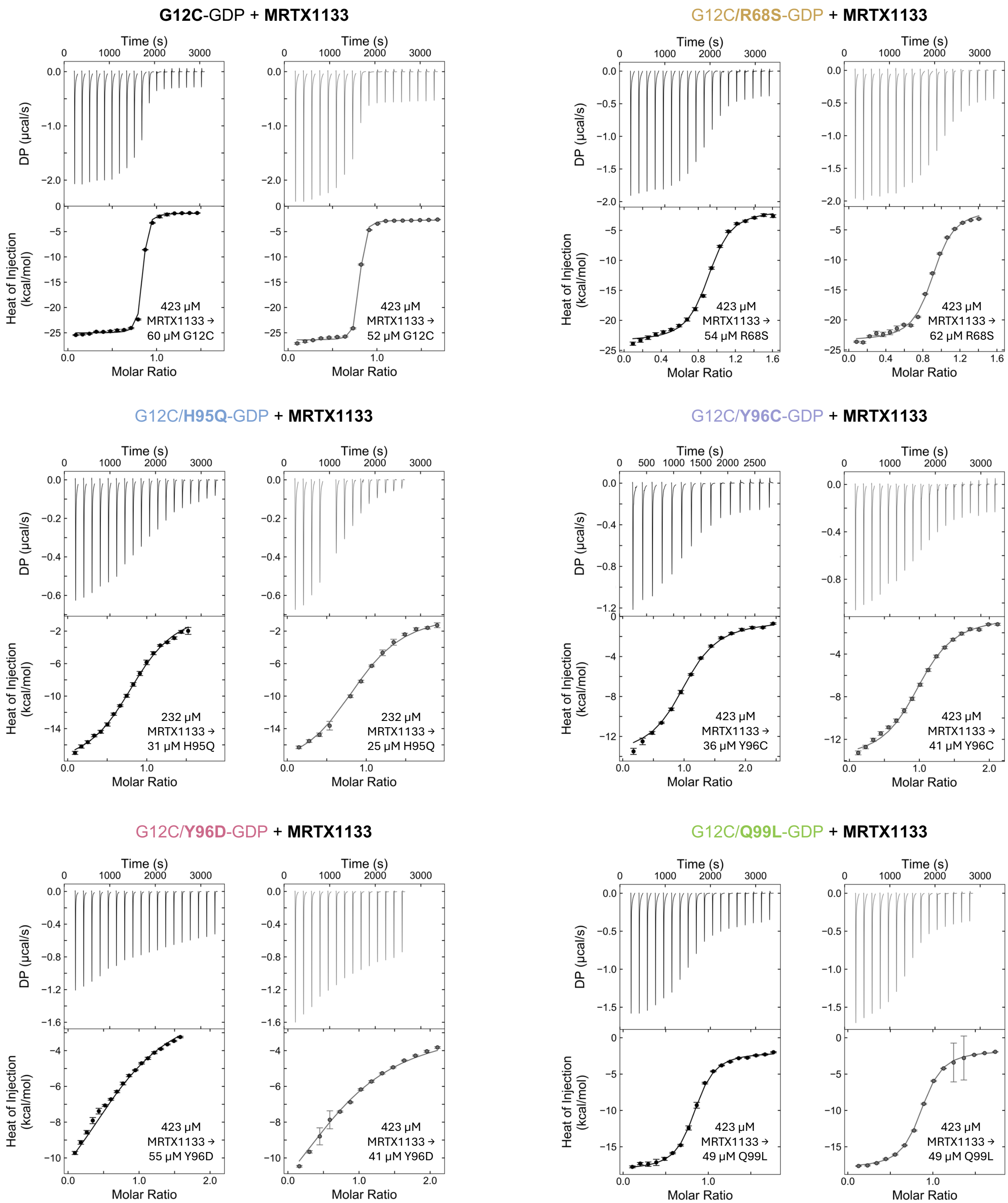

Supplementary Figure 4. ITC-derived binding affinities of RAS mutants for MRTX1133. Bar graphs of globally fitted dissociation constants (K_D) obtained from ITC measurements of MRTX1133 binding to KRAS G12C–GDP and the five resistance mutants. Error bars represent asymmetric 1σ confidence intervals from the global fitting of two independent measurements. The same data are plotted using a linear scale (left) and a logarithmic scale (right) on the y-axis. Source data are provided in the Source Data file.

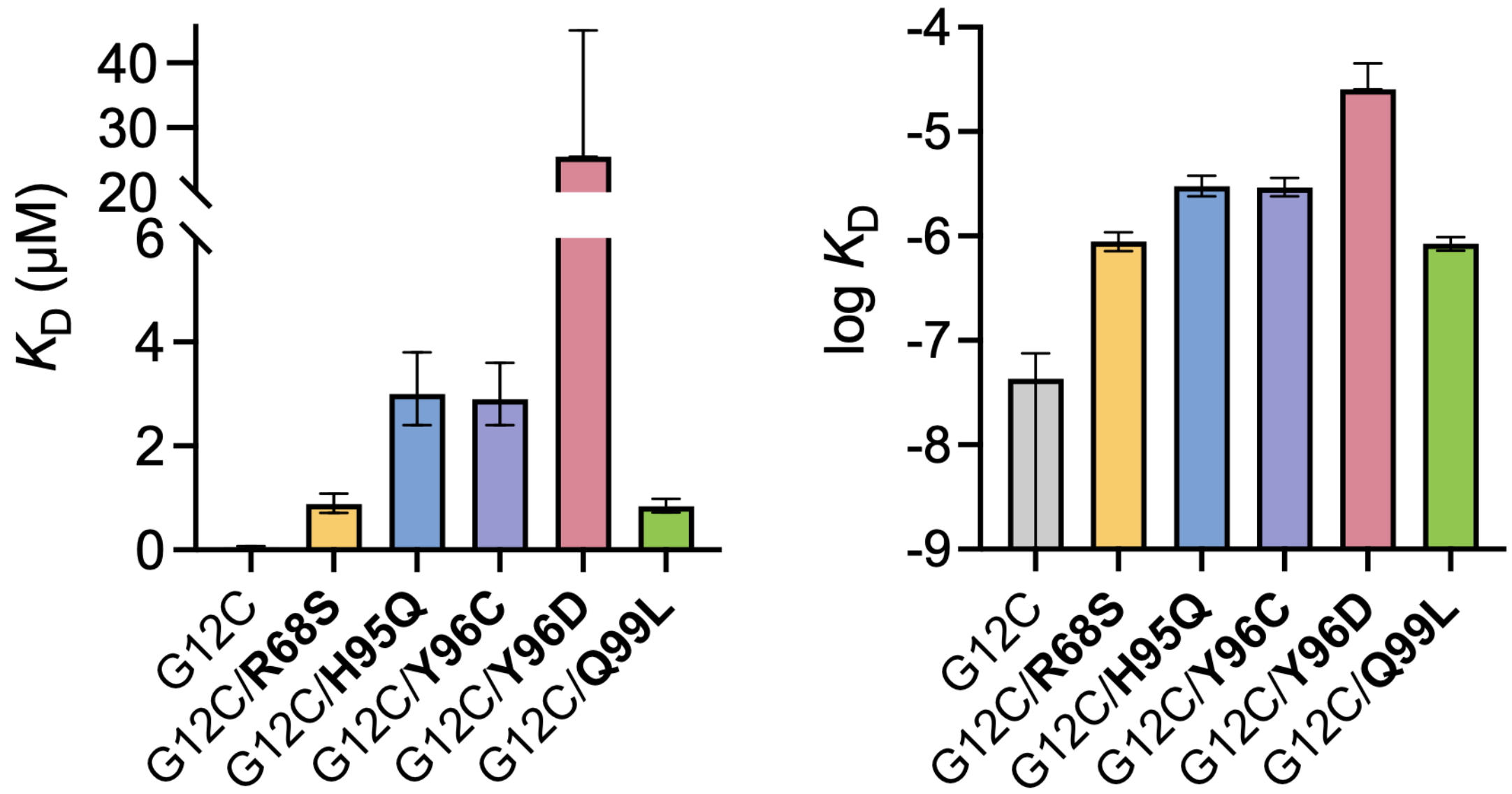

Supplementary Figure 5. Composite omit 2mFo-DFc electron density maps for the mutated residue in each KRAS G12C secondary resistance mutant. Electron density is contoured at 1σ for all mutants, except G12C/H95Q, for which density is contoured at 0.5σ due to reduced local map quality. Side chains at the mutation sites are shown as sticks and overlaid with the corresponding omit density.

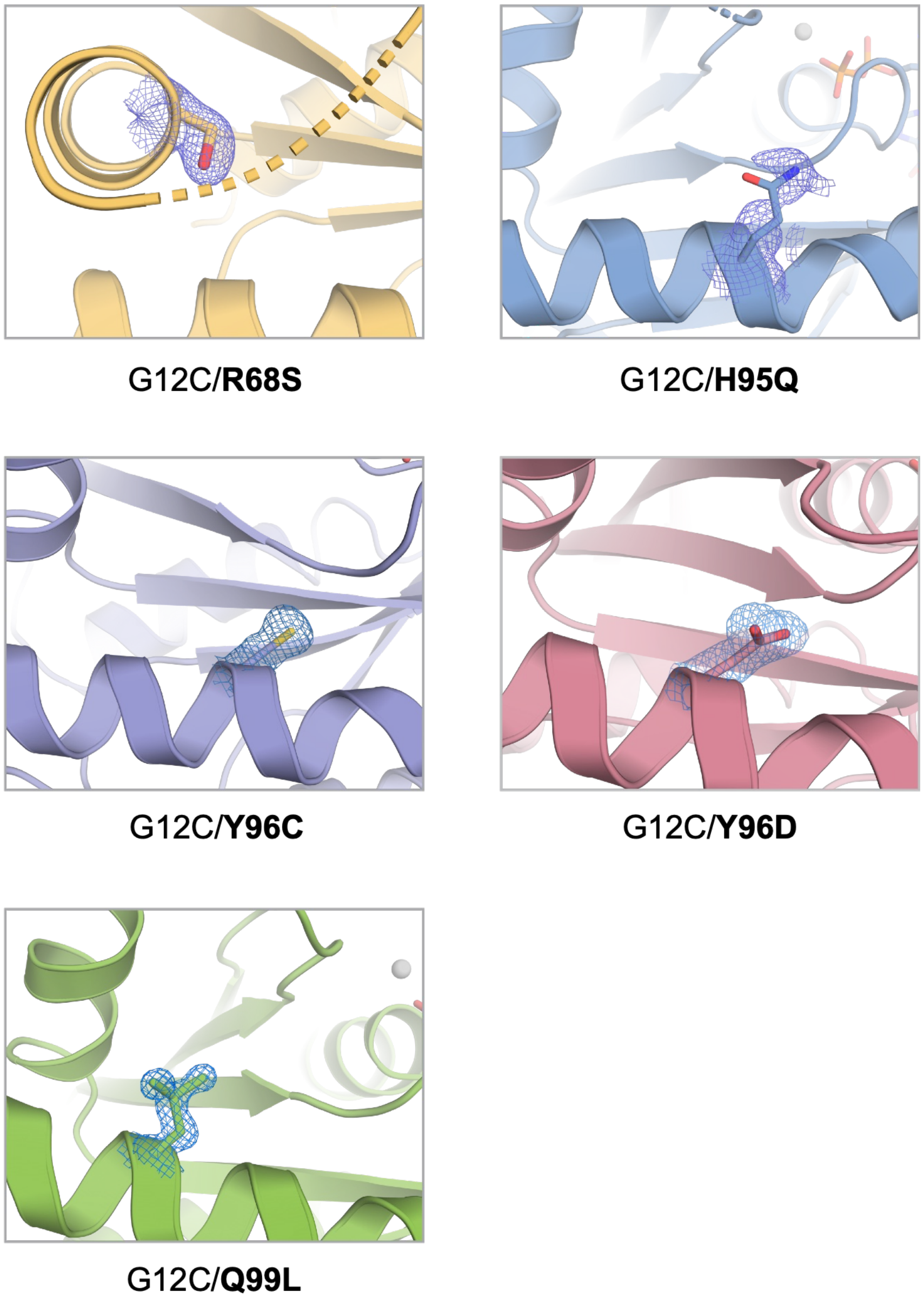

Supplementary Figure 6. Comparison of KRAS G12C/Y96D-GDP to KRAS G12D-GDP with noncovalently bound MRTX1133 (a) and KRAS G12C-GDP with covalently bound adagrasib (b) and BBO-8520 (c). The inhibitor is shown as cyan sticks within a transparent surface. The non-native D96-T58 interaction draws the backbone around T58 into the Switch-II pocket, resulting in steric occlusion of the binding pocket that reduces the binding of these three inhibitors in the same way as for sotorasib (Fig. 4c).

a

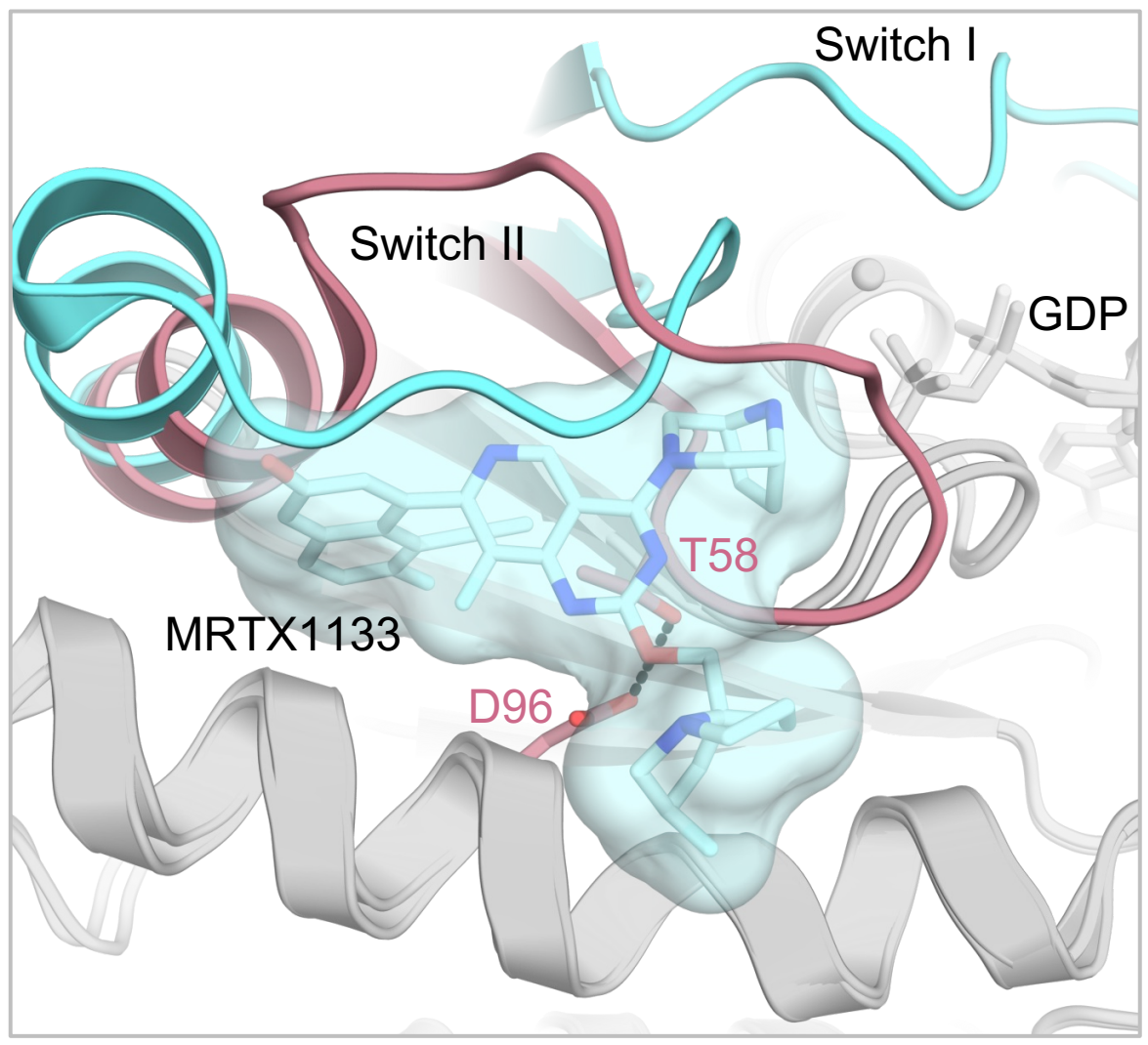

G12D (GDP)-MRTX1133
G12C/Y96D (GDP)

b

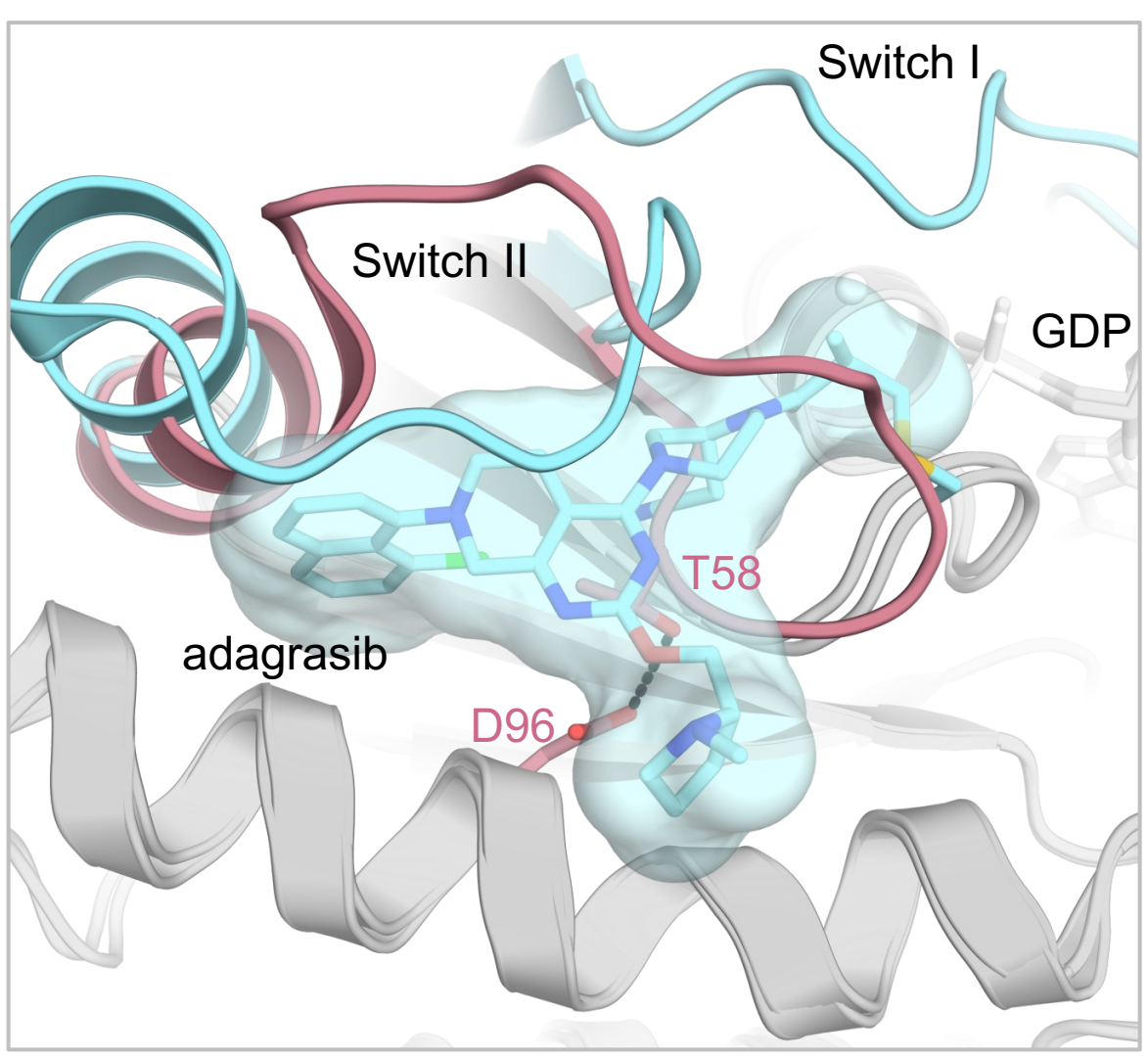

G12C (GDP)-adagrasib
G12C/Y96D (GDP)

c

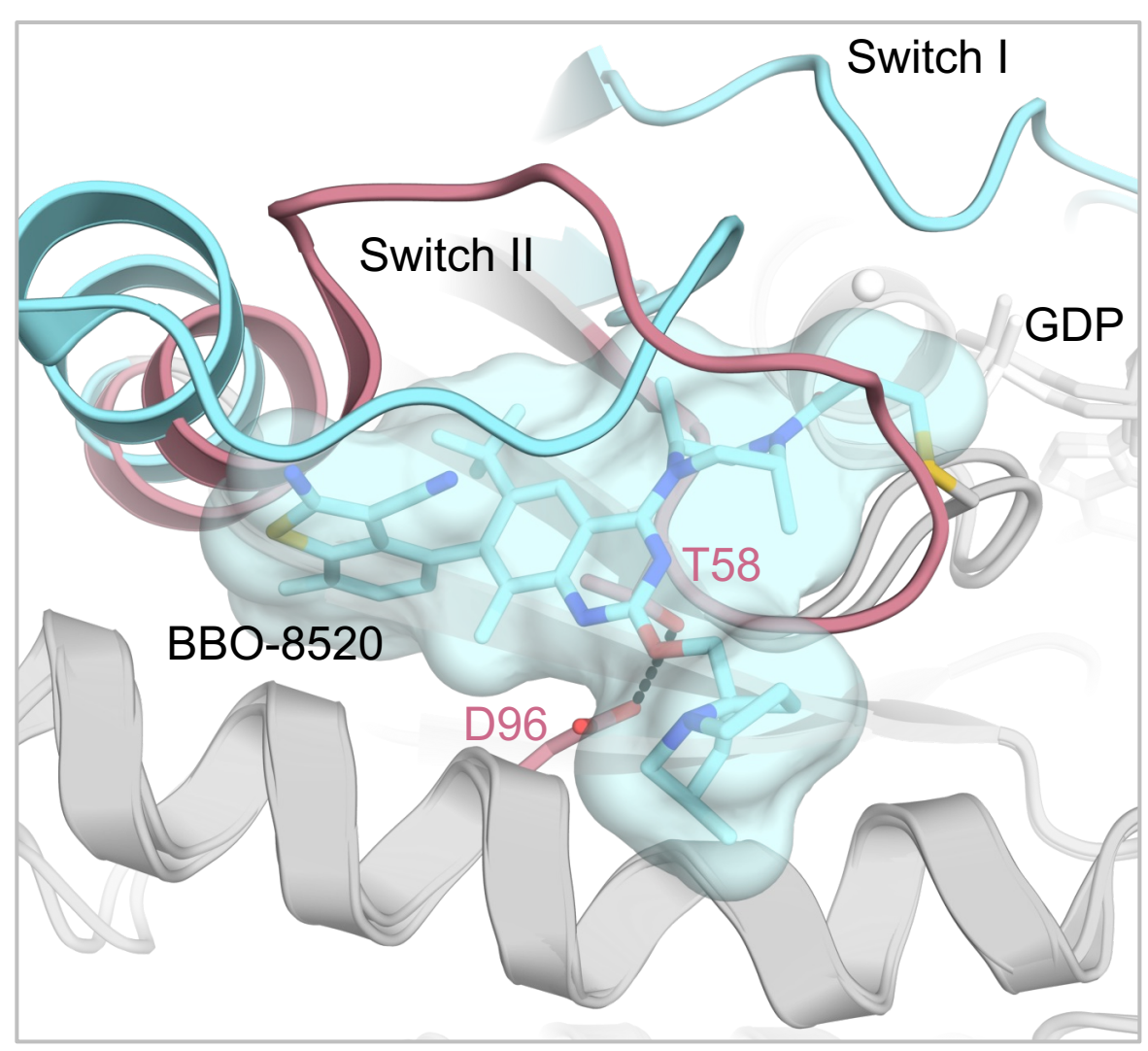

G12C (GDP)-BBO-8520
G12C/Y96D (GDP)

Supplementary Figure 7. Solution NMR analysis of the G12C/Y96D-GDP resistance mutant. Superposition of ^{1}H-^{15}N HSQC spectra of G12C-GDP (black) and G12C/Y96D-GDP (pink). The G12C/Y96D spectrum exhibits chemical shift perturbations compared to the G12C control; resonances for selected residues in either Switch-I or Switch-II are labeled in black. The G12C/Y96D spectrum also shows evidence of an alternate conformation of the protein in solution indicated by the presence of weaker secondary peaks for some resonances; these are labeled in pink.

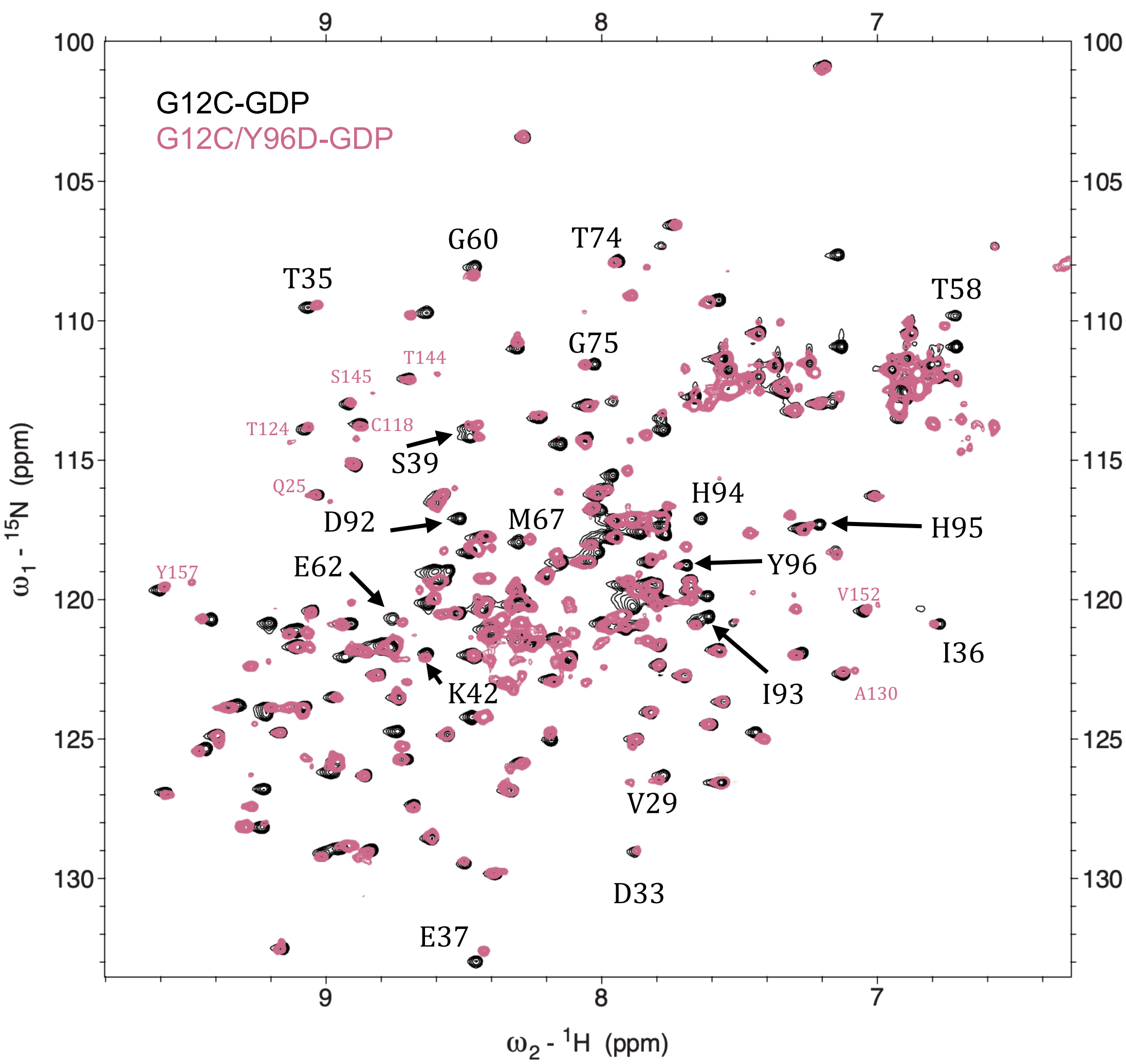

Supplementary Figure 8. Structural comparison of KRAS–GDP structures with a fully open Switch-I conformation. All KRAS–GDP structures in the PDB exhibiting a fully open Switch-I conformation were superimposed onto the G12C/Y96D–GDP structure determined in this study. The structures included are KRAS(2–169) (PDB 6M9W), KRAS-V14I (PDB 6MQG), KRAS-A146T (PDB 6BOF), KRAS(2–169)-G12D (PDB 7F0W), KRAS-A59E (PDB 7KMR), KRAS(2–169)-G13D (PDB 8CPR), KRAS-A146V (PDB 8EER), and KRAS-G12D/I55E (PDB 9C3L). Switch-II (residues 58–75) is shown in pink for G12C/Y96D and in yellow for all comparison structures. Despite the shared fully open Switch-I conformation, the Switch-II arrangement in G12C/Y96D is distinct, reflecting the non-native D96–T58 hydrogen bond that draws Switch-II into the pocket in the GDP-bound resistance mutant. (Top) Overall superposition of KRAS structures. (Bottom) Close-up view of Switch-II highlighting conformational differences.

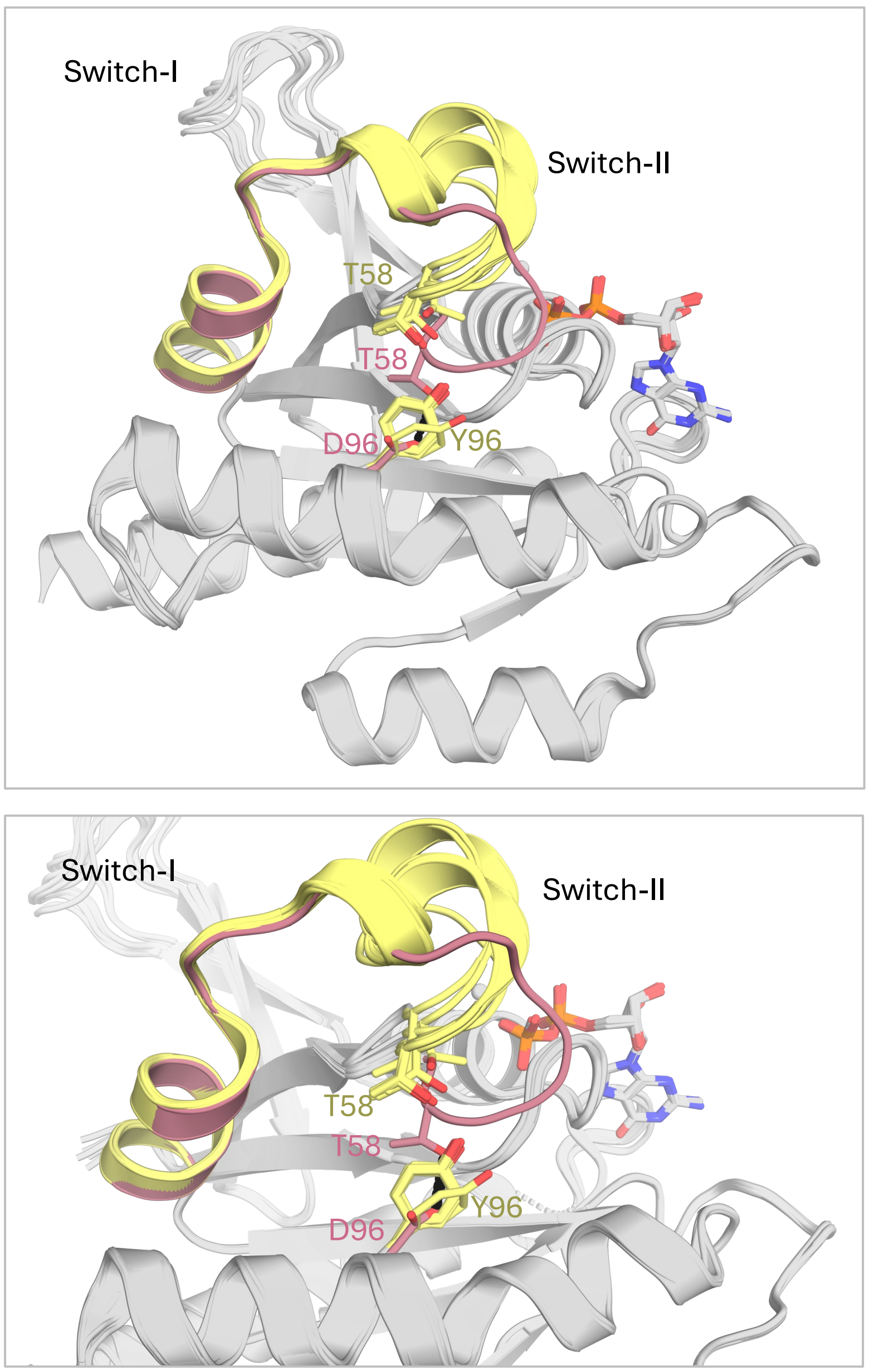

Supplementary Figure 9. ITC profiles for RAF1-RBD binding to GMPPNP-loaded KRAS G12C and secondary resistance mutants. Raw thermograms (top panels) and corresponding integrated heat plots (bottom panels) are shown for two independent experiments; the syringe and cell concentrations used in each experiment are given. The binding affinity was extracted by simultaneous global fitting of the two independent experiments (see Fig. 6b). The G12C/Q99L mutant exhibited a non-sigmoidal biphasic binding isotherm that could not be reliably described by a single-site model.

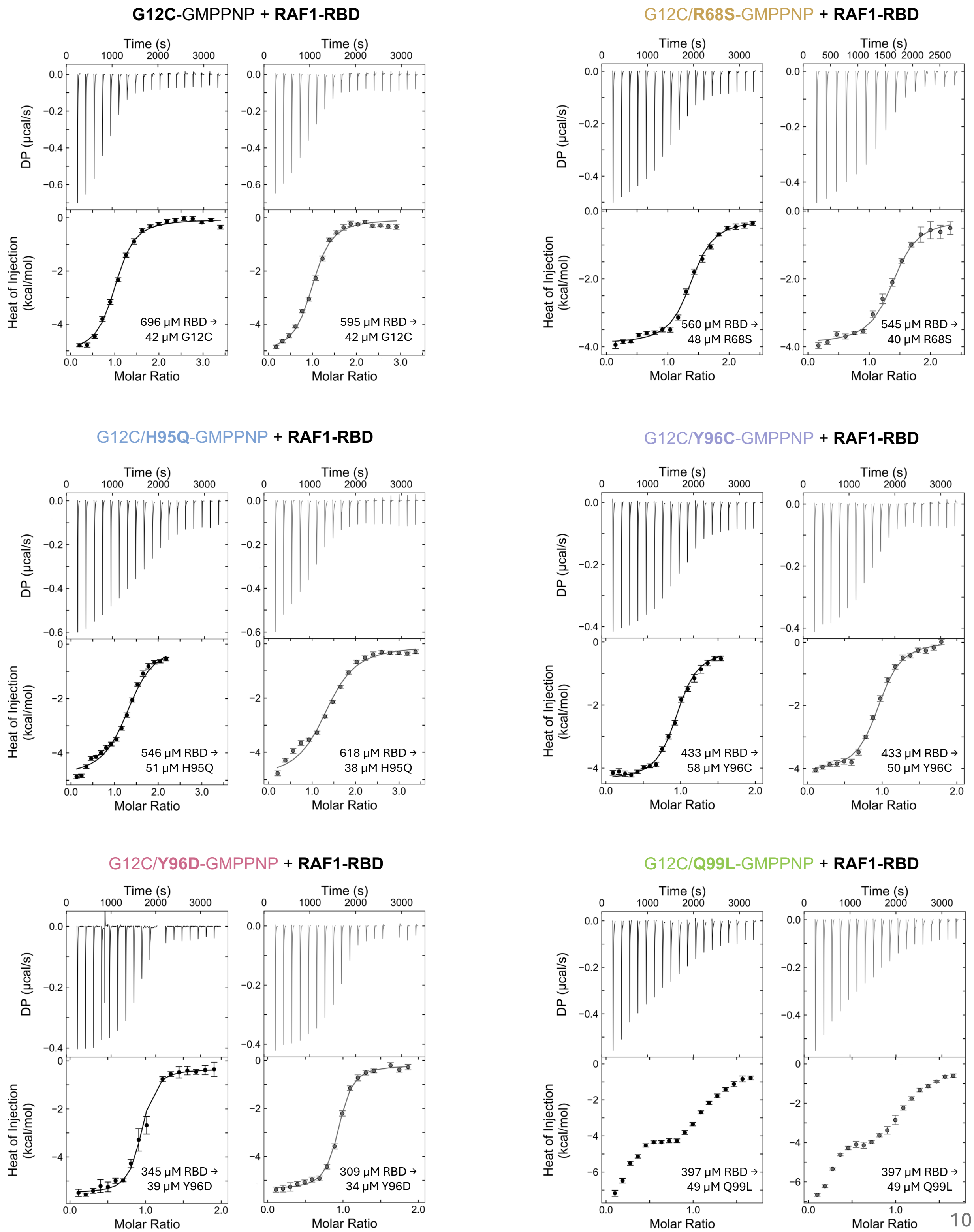

Supplementary Figure 10: SDS–PAGE analysis of KRAS / RAF1 RBD elution peaks from the analytical Superdex 75 column. In the presence of each KRAS resistance mutant, the RAF1 RBD co-elutes with KRAS, indicating stable complex formation. In the absence of KRAS, the RAF1 RBD elutes later in the chromatogram due to its smaller size. The uncropped gel image is available in the Source Data file.

Lane	Sample
1	RAF1 RBD
2	Molecular Weight Standards
3	Co-elution RAF1 RBD and G12C/R68S-GMPPNP
4	Co-elution RAF1 RBD and G12C/H95Q-GMPPNP
5	Co-elution RAF1 RBD and G12C/Y96C-GMPPNP
6	Co-elution RAF1 RBD and G12C/Y96D-GMPPNP
7	Co-elution RAF1 RBD and G12C/Q99L-GMPPNP

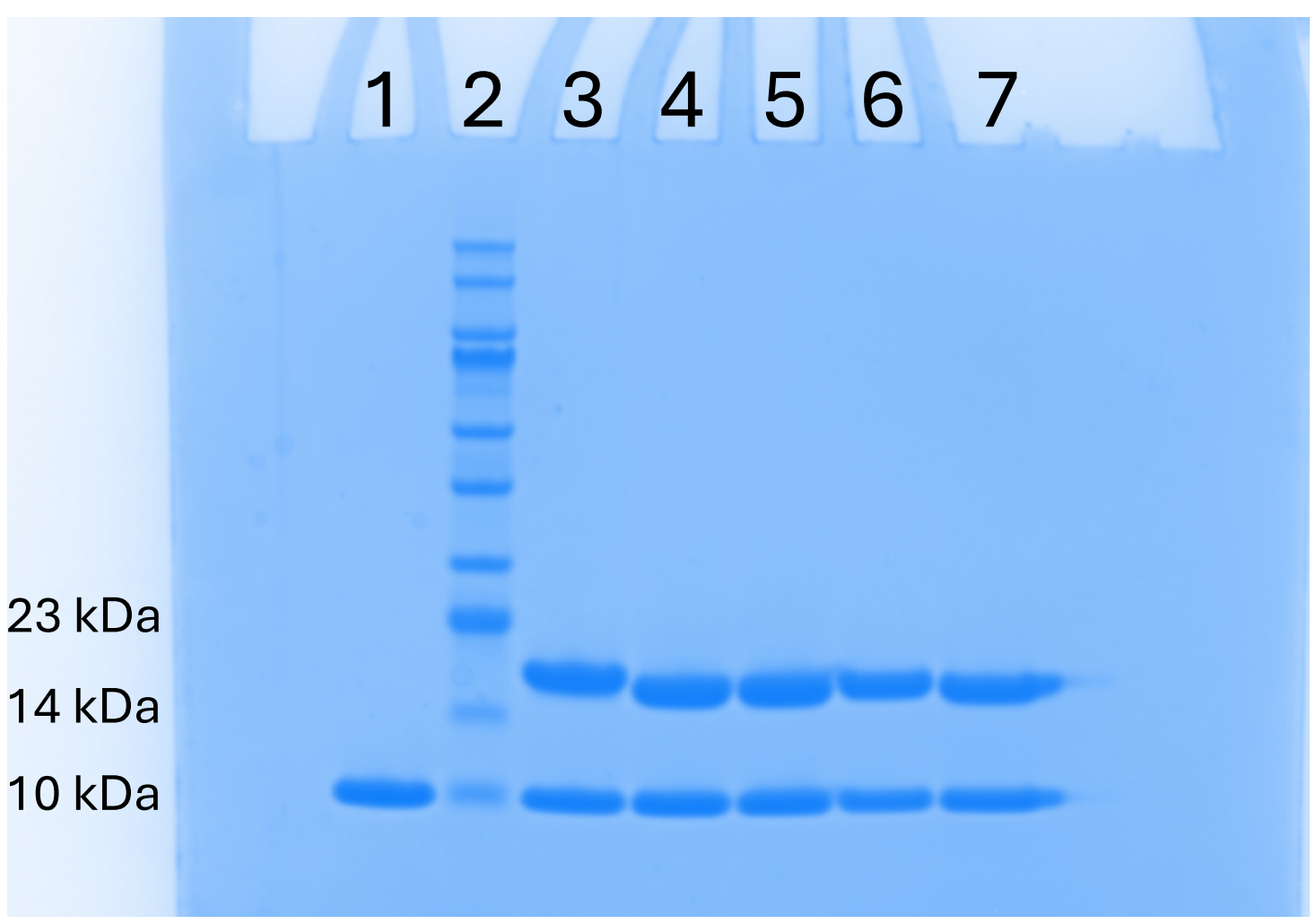

Supplementary Figure 11. ITC profiles for PI3Kα-RBD binding to GMPPNP-loaded KRAS G12C and secondary resistance mutants. Raw thermograms (top panels) and corresponding integrated heat plots (bottom panels) are shown for two independent experiments; the syringe and cell concentrations used in each experiment are given. The binding affinity was extracted by simultaneous global fitting of the two independent experiments (see Fig. 6e).

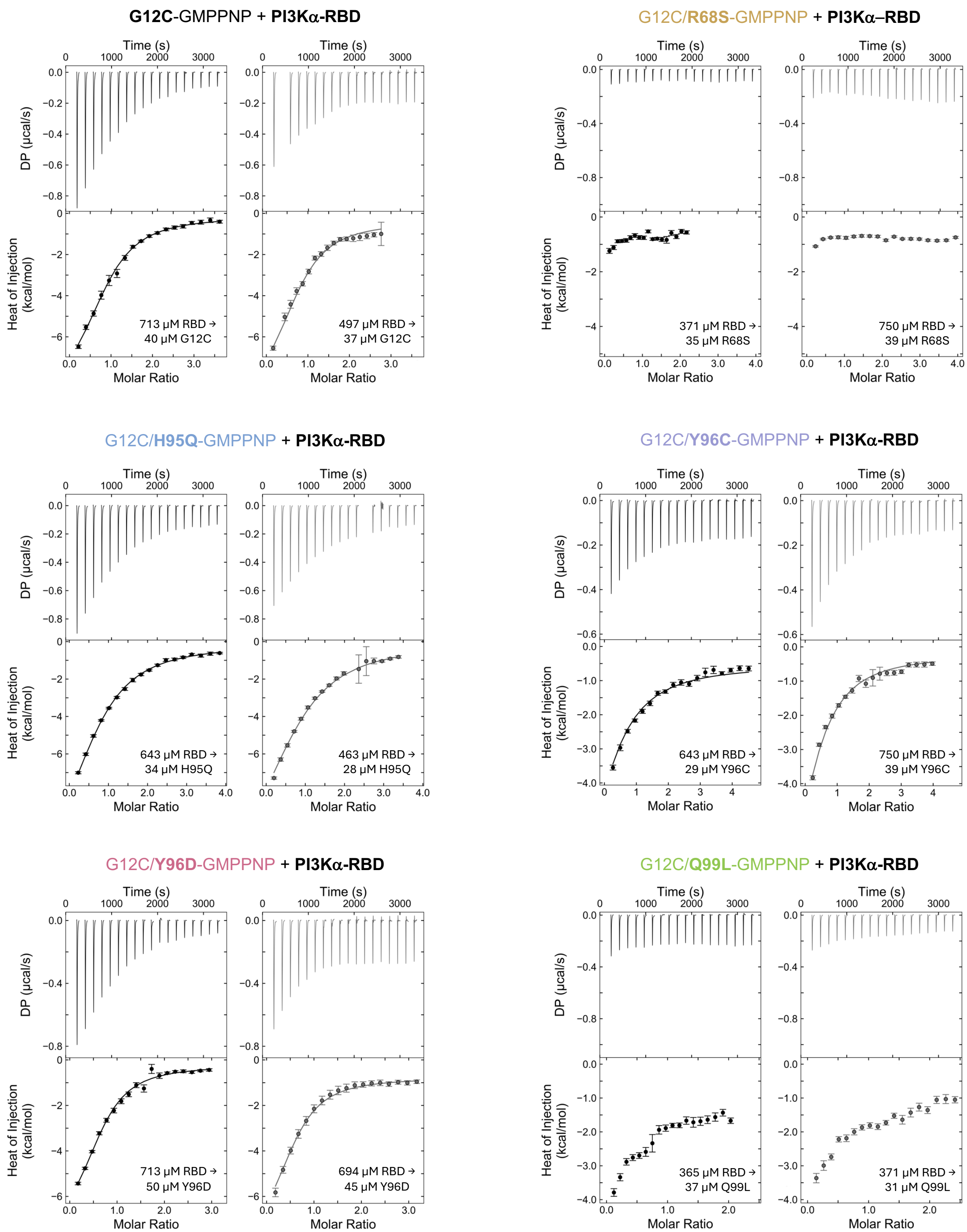

Supplementary Figure 12: AKT pathway modulation by KRAS G12C inhibitors across resistance mutants. Western blot analysis of AKT phosphorylation in HEK293 cells transiently expressing KRAS4b variants following treatment with the covalent KRAS G12C inhibitors BBO-8520, sotorasib, and adagrasib. Cells expressing (**a**) G12C, (**b**) G12C/R68S, (**c**) G12C/H95Q, (**d**) G12C/Y96C, (**e**) G12C/Y96D, and (**f**) G12C/Q99L were treated for 2 h with either DMSO or inhibitor at the indicated concentrations (BBO-8520 at 10 or 100 nM; sotorasib and adagrasib at 100 or 1000 nM). Levels of phosphorylated AKT (pAKT S473) and total AKT were assessed by immunoblotting. Total KRAS and vinculin are shown as expression and loading controls, respectively. KRAS band upshift indicates covalent target engagement, with band broadening indicating partial C12 population labeled with the drug in resistant mutants. The mean pAKT band intensity and standard deviation from three biological replicates are plotted for **(g)** G12C, **(h)** G12C/R68S, **(i)** G12C/H95Q, **(j)** G12C/Y96C, **(k)** G12C/Y96D, and **(l)** G12C/Q99L; individual data points are plotted as open circles. For each mutant, the pAKT band intensity in the presence of the drug is normalized with respect to the intensity of the DMSO-only control. Uncropped blots with labeled molecular weight standards are provided in the Source Data file.

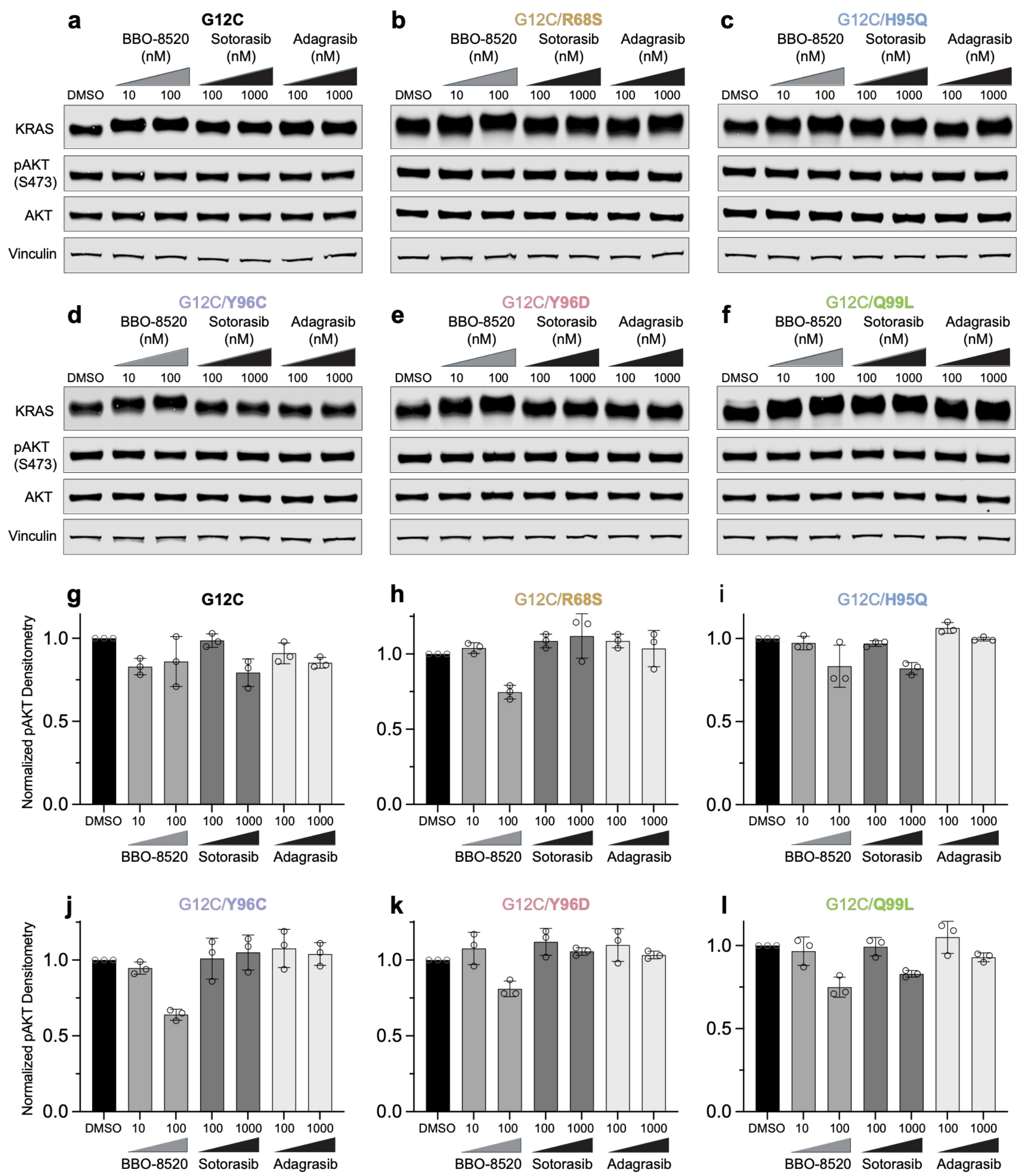

Supplementary Figure 13: RMSD plots for each of five replica MD simulations of G12C-GDP, G12C/R68S-GDP, G12C/H95Q-GDP, G12C/Y96D-GDP, G12C/Y96D-GTP, G12C/Y96C-GDP, and G12C/Q99L-GDP. Source data for all simulations are provided in the Source Data file.

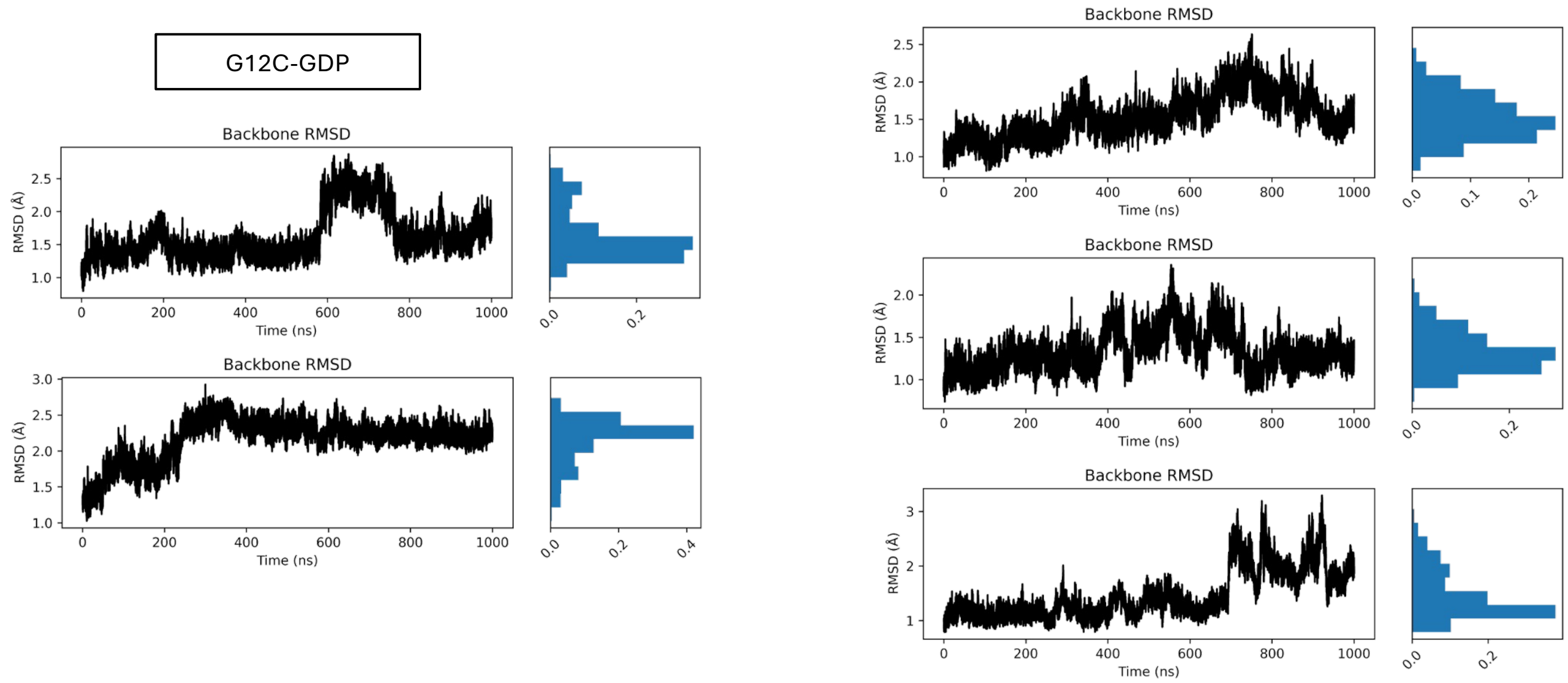

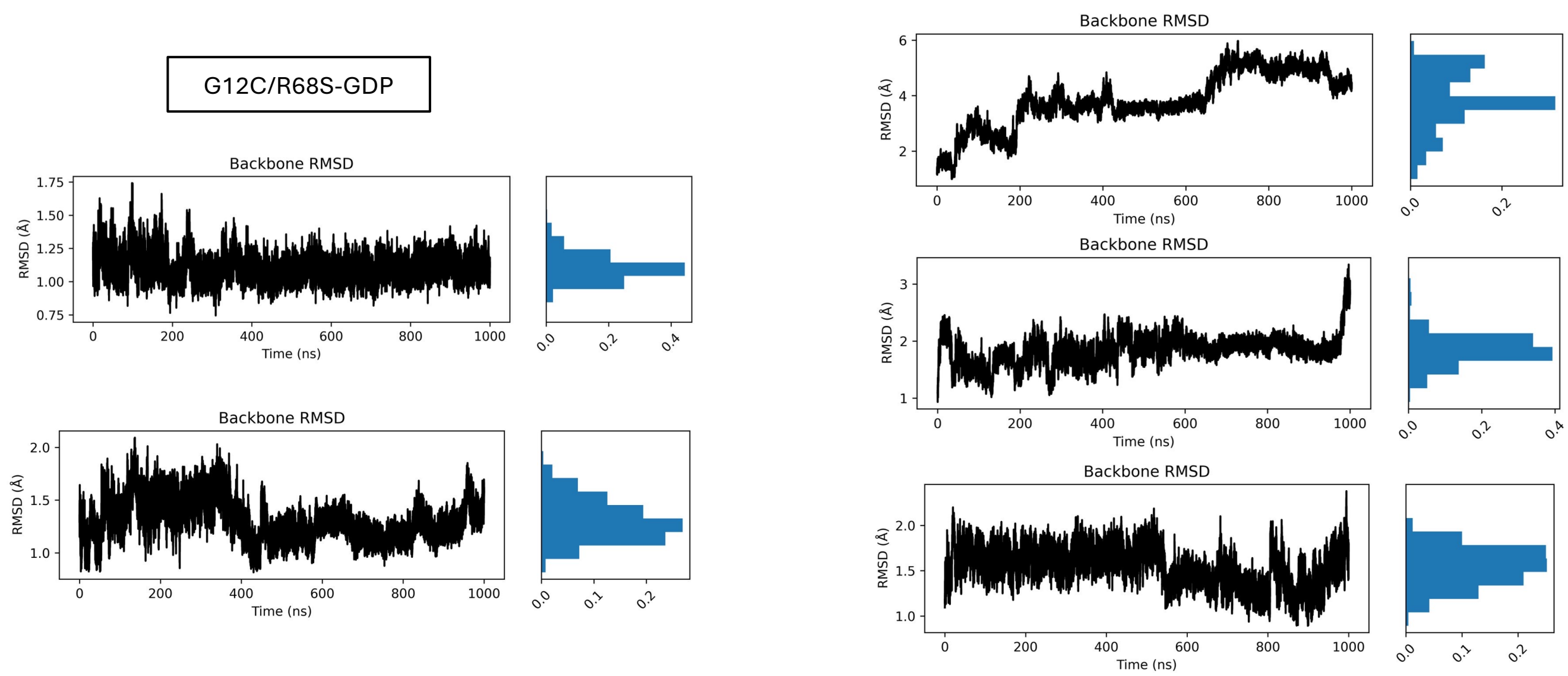

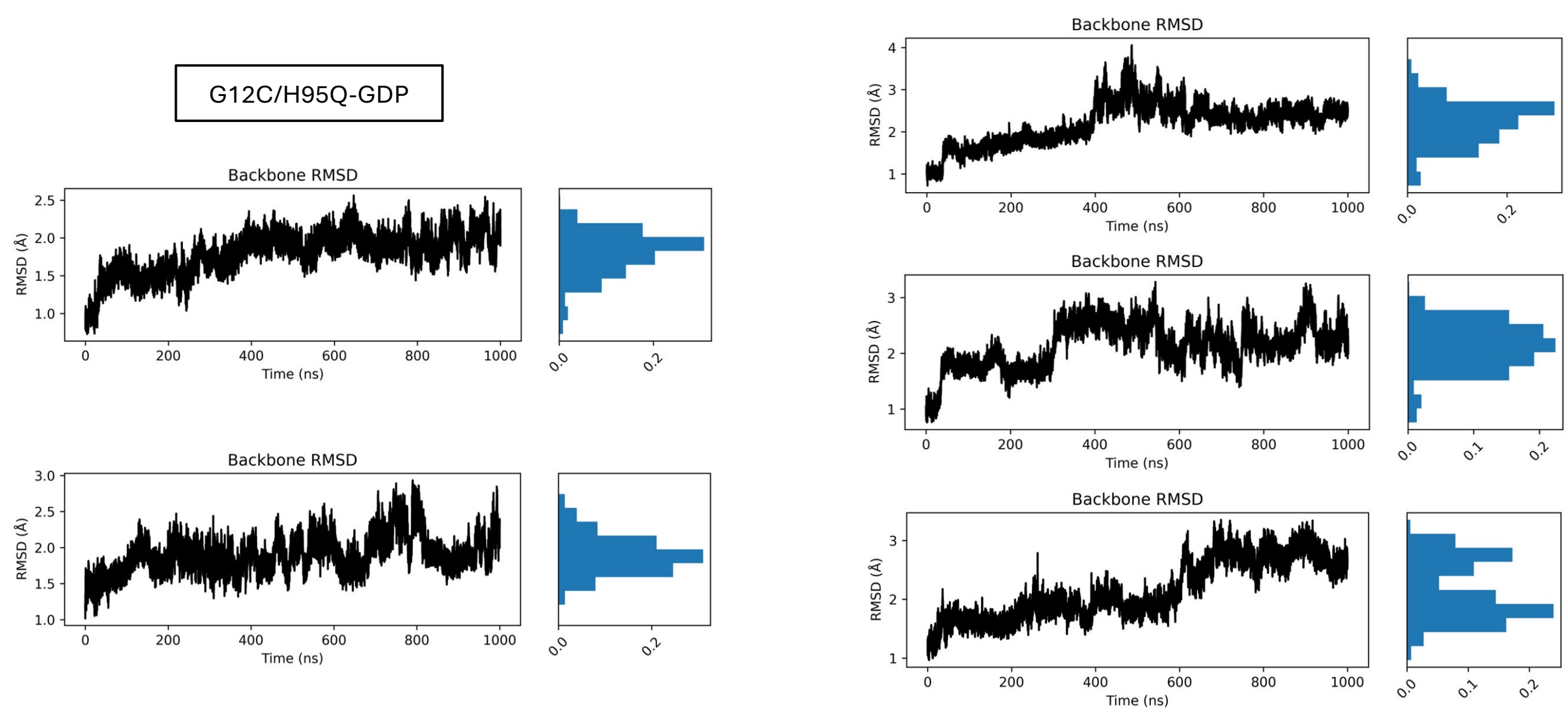

Supplementary Figure 13, continued

G12C/Y96D-GDP

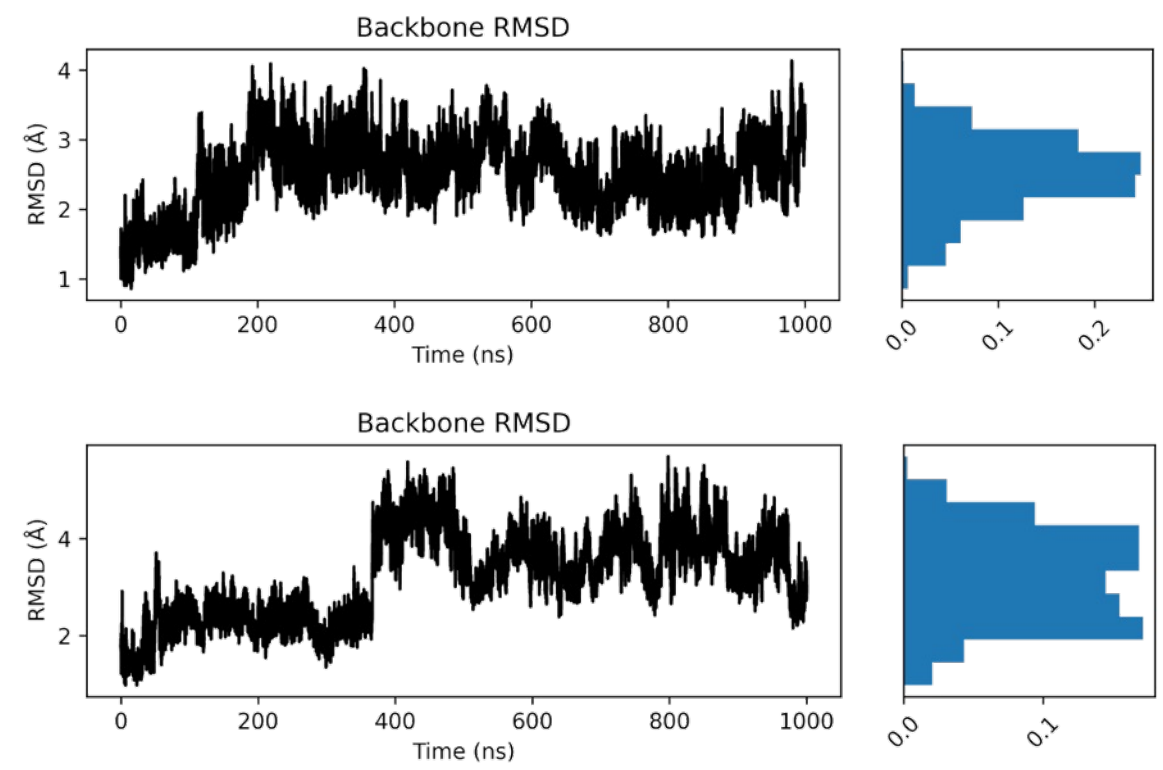

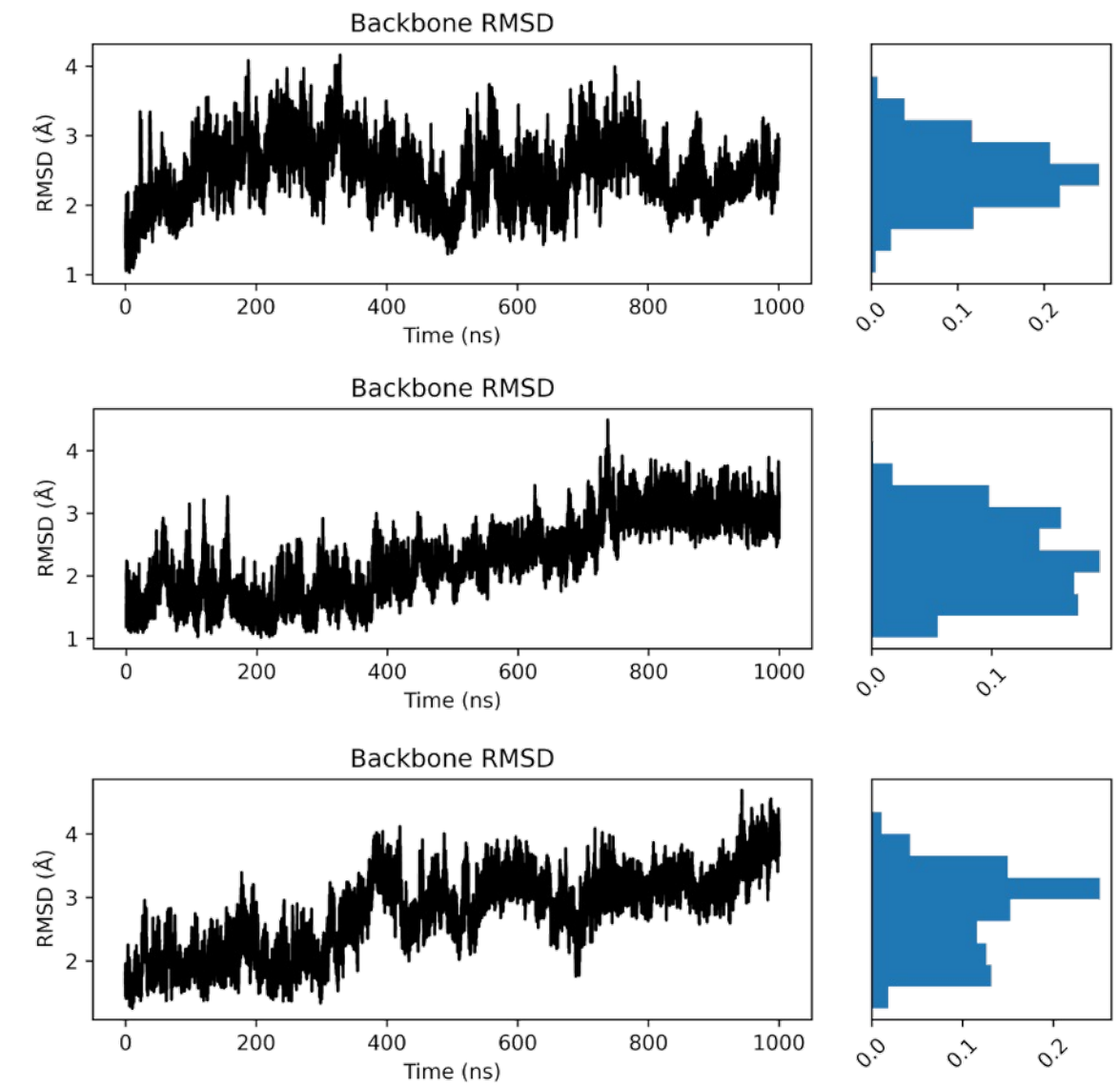

G12C/Y96D-GTP

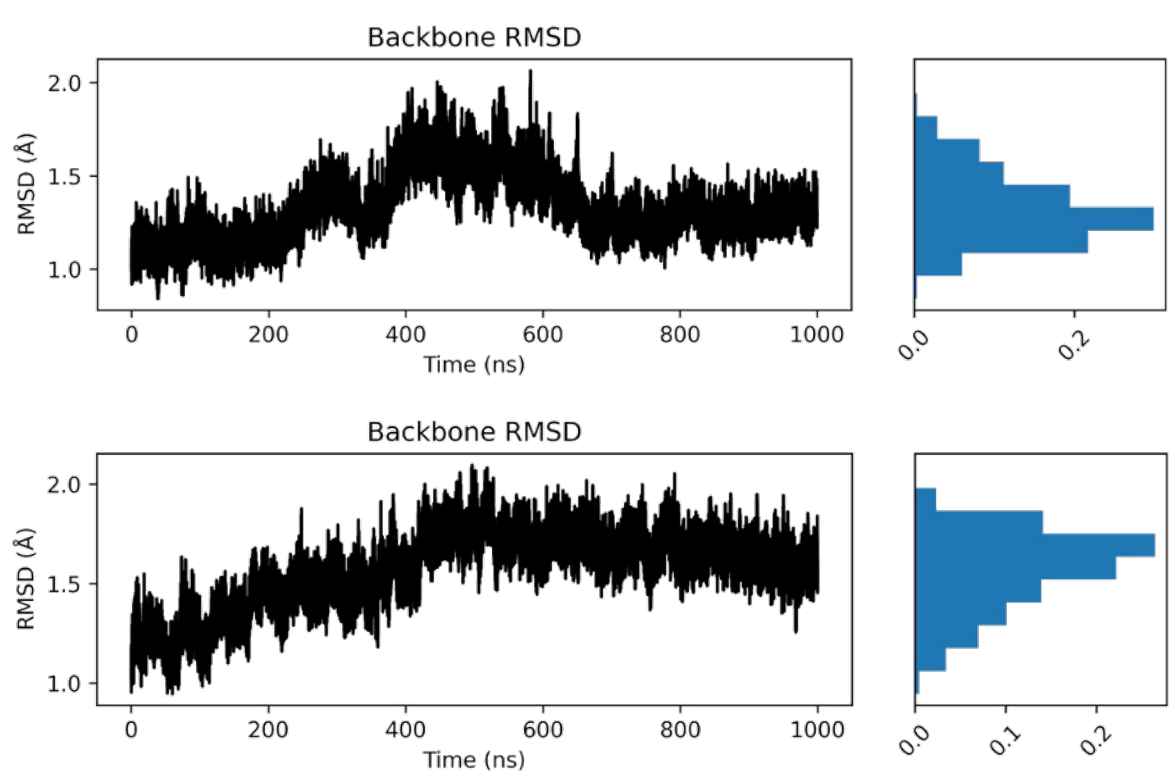

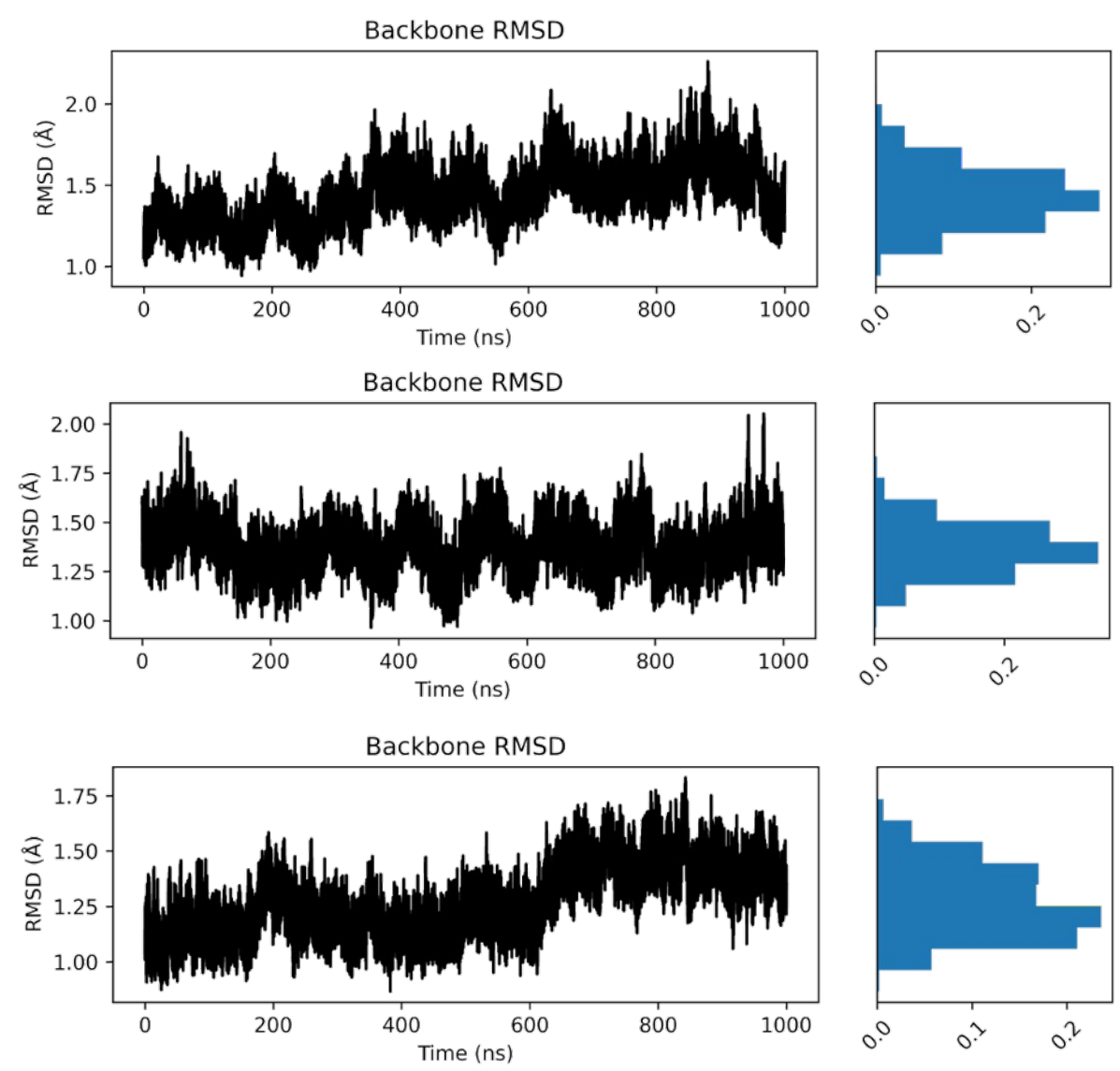

Supplementary Figure 13, continued

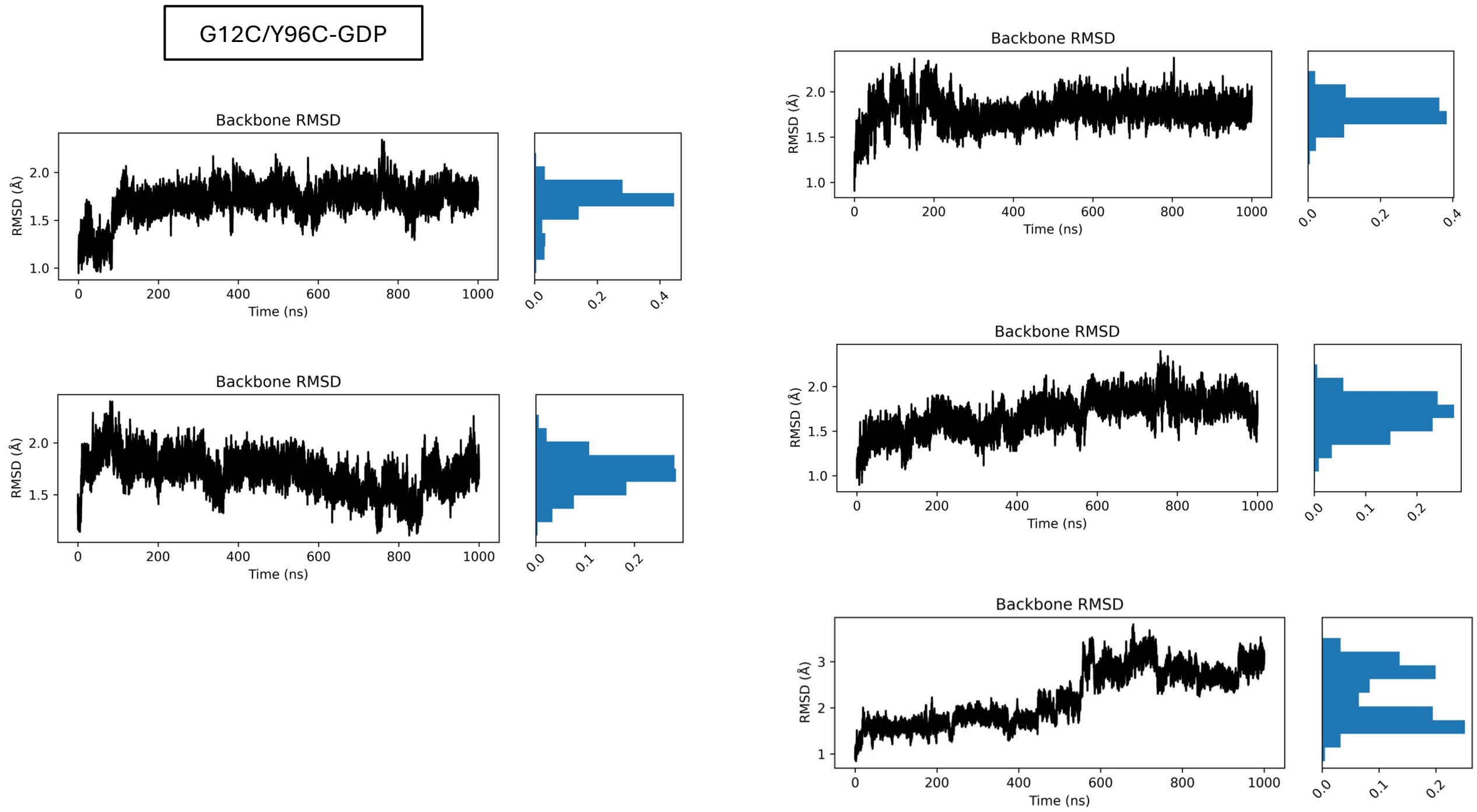

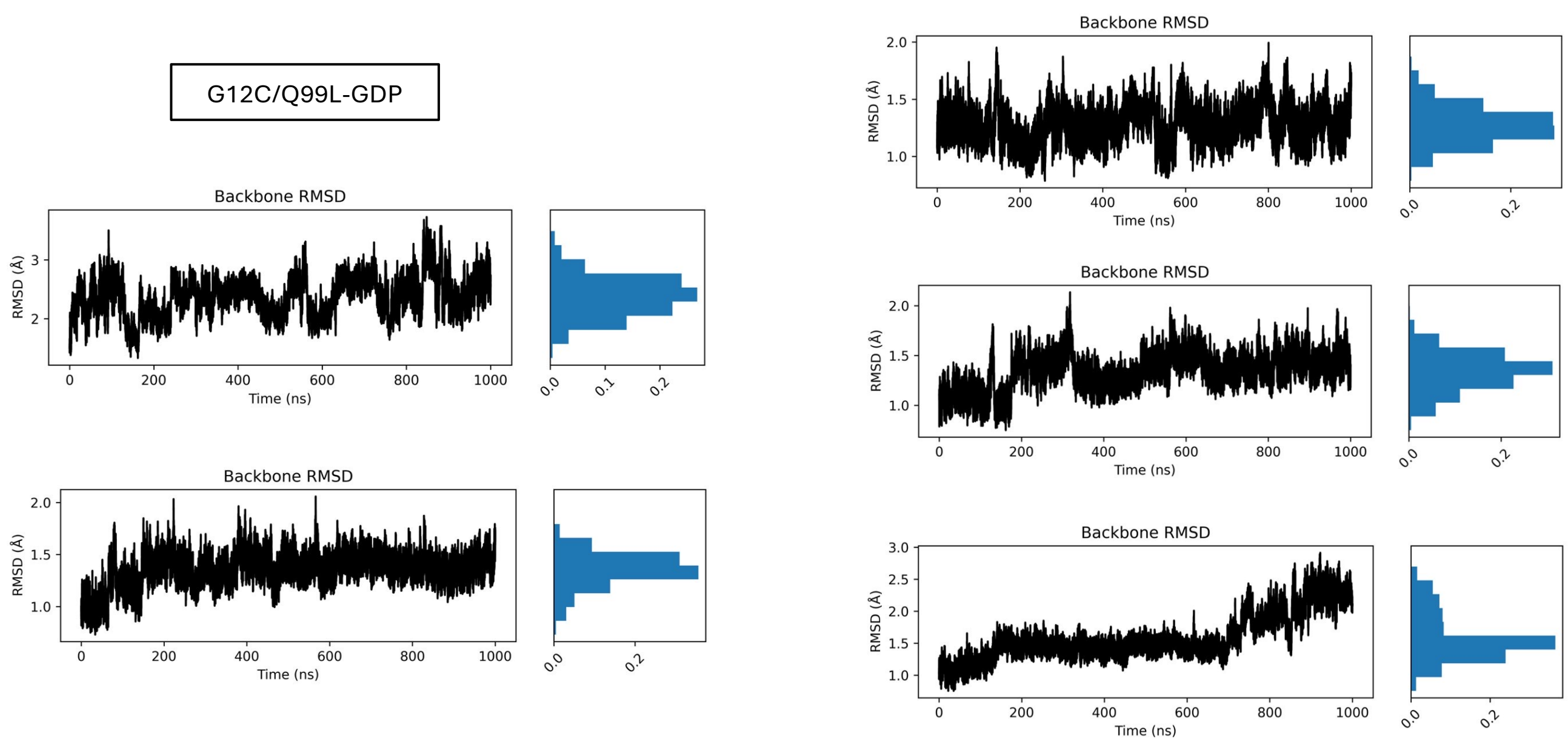

Supplementary Table 1: Protein concentrations and crystallization conditions for crystal structures solved in this study.

	Protein concentration and buffer composition	Crystallization cocktail	Incubation Temp (°C)
**KRAS4b(1-169)-G12C/R68S/C118S-GDP**	9.6 mg/mL in 40 mM MMT (pH 7.6), 50 mM NaCl, 10 mM $MgCl_2$, and 1 mM TCEP	30% w/v PEG 4000, 0.2 M $CaCl_2$, 0.2 M $MgCl_2$, and 0.1 M MES (pH 6.5)	20
**KRAS4b(1-169)-G12C/H95Q/C118S-GDP**	15 mg/mL in 10 mM MMT (pH 7.5), 10 mM magnesium formate, 40 mM NaCl, and 1 mM TCEP	1.6 M potassium sodium tartrate and 0.1 M MES (pH 6.5)	20
**KRAS4b(1-169)-G12C/Y96C/C118S-GDP**	13.7 mg/mL in 40 mM MMT (pH 7.9), 50 mM NaCl, 12 mM $MgCl_2$, and 1 mM TCEP	20% v/v glycerol ethoxylate, 3% v/v polyethyleneimine, and 0.1 M Tris (pH 8.5)	20
**KRAS4b(1-169)-G12C/Y96D/C118S-GDP**	13 mg/mL in 20 mM HEPES (pH 7.4), 150 mM NaCl, 5 mM $MgCl_2$, and 1 mM TCEP	2 M ammonium sulfate, 2% v/v PEG 400, and 0.1 M HEPES (pH 7.5)	20
**KRAS4b(1-169)-G12C/Q99L/C118S-GDP**	13.7 mg/mL in 40 mM MMT (pH 7.9), 50 mM NaCl, 10 mM $MgCl_2$, and 1 mM TCEP	30% w/v PEG MME 5000 and 0.1 M sodium acetate (pH 4.5)	20
**KRAS4b(1-169)-G12C/Y96D/C118S-GMPPNP + RAF1(RBD-CRD)**	206 µM complex in 20 mM MMT (pH 7.4), 115 mM NaCl, 25 mM magnesium acetate, and 1 mM TCEP	15% v/v PEG 400, 80 mM magnesium acetate, 50 mM sodium cacodylate (pH 6.5)	4

Supplementary Table 2: Analysis of the existence of the D96 interaction with either T58 or R68 in the G12C/Y96D resistance mutant in each of five 1-μs MD simulations.

Nucleotide	GDP		GTP (structure isolated from complex with RAF1)	
	% of frames containing interaction		% of frames containing interaction	
Replica	D96-T58	D96-R68	D96-T58	D96-R68
1	49.8	0.04	0	64.6
2	89.7	0.7	0	33.7
3	76.3	21.3	0	65.6
4	79.4	0.02	0	84.4
5	33.3	44	0	95.3

SUPPLEMENTARY METHODS

Clustering of molecular dynamics simulations and RMSF calculation

We used CPPTRAJ[1] to perform hierarchical agglomerative clustering of the MD simulation data for each system. First, trajectories from each simulation replicate were read in and backbone-fitted to their respective equilibrated reference, resulting in five separate 1-µs trajectories. During the clustering process, trajectory data across all five replicates were read and clustered together, using settings of *'epsilon 4.0 clusters 20 complete'* and *'sieve 10 random'*. Cluster representative structures were subsequently collected and visualized, providing a representation of the conformational variability of each system.

We also used CPPTRAJ to determine the Root Mean Square Fluctuation (RMSF) by fitting to the starting structure (before equilibration) and using it as the reference for an RMSF calculation. This was done by first reading in all the trajectories through *trajin*, reading in the starting structure as the reference called *startframe*, and then performing '*rms :[protein residue range] ref [startframe]* :*[protein residue range]*' to conduct an RMS fit, prior to computation of the RMSF using *'atomicfluct*' with the '*byres'* flag.

H-bonding analysis

Hydrogen bonding interactions between specific residues of interest (e.g., D96 – T58, D96 – R68) were computed using the *MDAnalysis-2.8.0* Python package[2] within a Jupyter interactive notebook environment (version 7.3.1) using Python 3.10.16.

Specifically, after loading the combined trajectories for each system into an MDAnalysis *Universe* (5 µs of sampling data), the *HydrogenBondAnalysis* module was invoked using *'from MDAnalysis.analysis.hydrogenbonds.hbond_analysis import HydrogenBondAnalysis as HBA'* and providing an appropriate set of residues and atoms for the donor, acceptor, and hydrogen atom selections, using a cutoff of 3.5 Å for an H-bond interaction, and the default angle of 150 degrees for the donor-hydrogen-acceptor angle cutoff. The per-replicate percentage of frames with H-bonds present was then quantified and tabulated.

SUPPLEMENTARY REFERENCES

1. Roe DR, Cheatham TE, 3rd. PTRAJ and CPPTRAJ: Software for Processing and Analysis of Molecular Dynamics Trajectory Data. *J Chem Theory Comput* **9**, 3084–3095 (2013).

2. Gowers R*, et al.* MDAnalysis: A Python Package for the Rapid Analysis of Molecular Dynamics Simulations. In: *Proceedings of the 15th Python in Science Conference*) (2016).
