# Supplementary material for: Structural mechanisms underlying resistance to Switch-II pocket inhibitors in KRAS
Source: Nat Commun. 2026 Sep 29;17:11229. doi: 10.1038/s41467-026-78133-4 (PMC13720030; doi:10.1038/s41467-026-78133-4)
Supplement: Supplementary file 2 [file 41467_2026_78133_MOESM2_ESM.pdf]

nature portfolio

Corresponding author(s): Dhirendra Simanshu
Last updated by author(s): Aug 17, 2026

Reporting Summary

Nature Portfolio wishes to improve the reproducibility of the work that we publish. This form provides structure for consistency and transparency in reporting. For further information on Nature Portfolio policies, see our Editorial Policies and the Editorial Policy Checklist.

Statistics

For all statistical analyses, confirm that the following items are present in the figure legend, table legend, main text, or Methods section.

n/a	Confirmed	
	x	The exact sample size (*n*) for each experimental group/condition, given as a discrete number and unit of measurement
	x	A statement on whether measurements were taken from distinct samples or whether the same sample was measured repeatedly
x		The statistical test(s) used AND whether they are one- or two-sided<br>*Only common tests should be described solely by name; describe more complex techniques in the Methods section.*
x		A description of all covariates tested
x		A description of any assumptions or corrections, such as tests of normality and adjustment for multiple comparisons
	x	A full description of the statistical parameters including central tendency (e.g. means) or other basic estimates (e.g. regression coefficient) AND variation (e.g. standard deviation) or associated estimates of uncertainty (e.g. confidence intervals)
x		For null hypothesis testing, the test statistic (e.g. *F*, *t*, *r*) with confidence intervals, effect sizes, degrees of freedom and *P* value noted<br>*Give P values as exact values whenever suitable.*
x		For Bayesian analysis, information on the choice of priors and Markov chain Monte Carlo settings
x		For hierarchical and complex designs, identification of the appropriate level for tests and full reporting of outcomes
x		Estimates of effect sizes (e.g. Cohen's *d*, Pearson's *r*), indicating how they were calculated

Our web collection on statistics for biologists contains articles on many of the points above.

Software and code

Policy information about availability of computer code

Data collection	No software was used.

Data analysis

Applied Biosystems Protein Thermal Shift software (v 1.3)
NITPIC (v 2.1.5)
SEDPHAT (v 15.2b)
GUSSI (v 2.1.6)
GraphPad Prism (v 10.6.1)
XDS (v 19 Jan 2025)
EPMR (v 16.07.1)
ARP/wARP (v 8.0)
ModelCraft (v 5.0.3)
Coot (v 0.9.8 and 1.1.19)
Phenix (v 1.21.2-5419)
PyMOL (v 1.3)
UCSF Chimera (v 1.169)
AMBER24
AMBERTools 25
Rosetta Suite (release #371)
POKY (build 20240829)
LI-COR Image Studio (v 5.2)

For manuscripts utilizing custom algorithms or software that are central to the research but not yet described in published literature, software must be made available to editors and reviewers. We strongly encourage code deposition in a community repository (e.g. GitHub). See the Nature Portfolio guidelines for submitting code & software for further information.

Data

Policy information about availability of data

All manuscripts must include a data availability statement. This statement should provide the following information, where applicable:

- Accession codes, unique identifiers, or web links for publicly available datasets
- A description of any restrictions on data availability
- For clinical datasets or third party data, please ensure that the statement adheres to our policy

Atomic coordinates and structure factors for structures determined in this study have been deposited in the Protein Data Bank (PDB) under accession codes 11FU (KRAS G12C/R68S-GDP), 11ES (KRAS G12C/H95Q-GDP), 11FB (KRAS G12C/Y96C-GDP), 11FL (KRAS G12C/Y96D-GDP), 11GH (KRAS G12C/Y96D-GMPPNP with RAF1 RBD-CRD), and 11FT (KRAS G12C/Q99L-GDP). Previously deposited structures used for analysis, comparison, or superposition included KRAS G12C-GDP (PDB 4LDJ); KRAS G12C-GDP bound to sotorasib (PDB 6OIM), adagrasib (PDB 6UT0), divarasib (PDB 9DMM), opnurasib (PDB 7R0M), glecirasib (PDB 9KPN), calderasib (PDB 8S8C), BI-0474 (PDB 8AFB), and BBO-8520 (PDB 8V3A); KRAS G12C-GMPPNP bound to BBO-8520 (PDB 8V39); KRAS G12D-GMPPCP bound to MRTX1133 (PDB 7T47); KRAS G12C-GDP bound to BI-2865 (PDB 8AZX); KRAS G12D-GDP bound to BBO-11818 (PDB 9P44); KRAS-RAF1 RBD (PDB 6VJJ); KRAS-RAF1 RBD-CRD (PDB 6XHB); and KRAS-PI3Ka (PDB 9C15). Additional data supporting the findings of this study are available from the corresponding author upon reasonable request.

Research involving human participants, their data, or biological material

Policy information about studies with human participants or human data. See also policy information about sex, gender (identity/presentation), and sexual orientation and race, ethnicity and racism.

Reporting on sex and gender

Use the terms sex (biological attribute) and gender (shaped by social and cultural circumstances) carefully in order to avoid confusing both terms. Indicate if findings apply to only one sex or gender; describe whether sex and gender were considered in study design; whether sex and/or gender was determined based on self-reporting or assigned and methods used.
Provide in the source data disaggregated sex and gender data, where this information has been collected, and if consent has been obtained for sharing of individual-level data; provide overall numbers in this Reporting Summary. Please state if this information has not been collected.
Report sex- and gender-based analyses where performed, justify reasons for lack of sex- and gender-based analysis.

Reporting on race, ethnicity, or other socially relevant groupings

Please specify the socially constructed or socially relevant categorization variable(s) used in your manuscript and explain why they were used. Please note that such variables should not be used as proxies for other socially constructed/relevant variables (for example, race or ethnicity should not be used as a proxy for socioeconomic status).
Provide clear definitions of the relevant terms used, how they were provided (by the participants/respondents, the researchers, or third parties), and the method(s) used to classify people into the different categories (e.g. self-report, census or administrative data, social media data, etc.)
Please provide details about how you controlled for confounding variables in your analyses.

Population characteristics

Describe the covariate-relevant population characteristics of the human research participants (e.g. age, genotypic information, past and current diagnosis and treatment categories). If you filled out the behavioural & social sciences study design questions and have nothing to add here, write "See above."

Recruitment

Describe how participants were recruited. Outline any potential self-selection bias or other biases that may be present and how these are likely to impact results.

Ethics oversight

Identify the organization(s) that approved the study protocol.

Note that full information on the approval of the study protocol must also be provided in the manuscript.

Field-specific reporting

Please select the one below that is the best fit for your research. If you are not sure, read the appropriate sections before making your selection.

- [x] Life sciences
- [ ] Behavioural & social sciences
- [ ] Ecological, evolutionary & environmental sciences

For a reference copy of the document with all sections, see nature.com/documents/nr-reporting-summary-flat.pdf

Life sciences study design

All studies must disclose on these points even when the disclosure is negative.

Sample size	*Describe how sample size was determined, detailing any statistical methods used to predetermine sample size OR if no sample-size calculation was performed, describe how sample sizes were chosen and provide a rationale for why these sample sizes are sufficient.*
Data exclusions	No data were excluded.
Replication	kinact/KI measurements were made using 4-7 individual protein:drug ratios. All ITC experiments involved two technical replicates and were analyzed via global fitting. DSF measurements involved three technical replicates. Details of all replicate measurements are found in the Methods section of the manuscript.
Randomization	*Describe how samples/organisms/participants were allocated into experimental groups. If allocation was not random, describe how covariates were controlled OR if this is not relevant to your study, explain why.*
Blinding	Blinding not relevant to this study.

Reporting for specific materials, systems and methods

We require information from authors about some types of materials, experimental systems and methods used in many studies. Here, indicate whether each material, system or method listed is relevant to your study. If you are not sure if a list item applies to your research, read the appropriate section before selecting a response.

Materials & experimental systems

n/a	Involved in the study	
☐	☒	Antibodies
☐	☒	Eukaryotic cell lines
☒	☐	Palaeontology and archaeology
☒	☐	Animals and other organisms
☒	☐	Clinical data
☒	☐	Dual use research of concern
☒	☐	Plants

Methods

n/a	Involved in the study	
☒	☐	ChIP-seq
☒	☐	Flow cytometry
☒	☐	MRI-based neuroimaging

Antibodies

Antibodies used	Phospho-Akt (Ser473) (D9E) Rabbit mAb, Cell Signaling Technology, cat# 4060, dilution 1:1000<br>Akt (pan) (40D4) Mouse mAb, Cell Signaling Technology, cat# 2920, dilution 1:1000<br>Phospho-p44/42 MAPK (Erk1/2) (Thr202/Tyr204) (D13.14.4E) Rabbit mAb, Cell Signaling Technology, cat #4370, dilution 1:1000<br>p44/42 MAPK (Erk1/2) (L34F12) Mouse mAb, Cell Signaling Technology, cat# 4696, dilution 1:1000<br>Vinculin (E1E9V) Rabbit mAb, Cell Signaling Technology, cat# 13901, dilution 1:1000<br>KRAS (3B10-2F2) Mouse mAb, Novus Biologicals, cat# H00003845-M01, dilution 1:500
Validation	For all antibodies, quality control and validation were performed by the manufacturers.

Eukaryotic cell lines

Policy information about cell lines and Sex and Gender in Research

Cell line source(s)	HEK293 and HEK293T cells, female origin, were acquired internally from the FNL Cell Development Group.
Authentication	Cell lines were authenticated using STR profiling.
Mycoplasma contamination	All cells were tested for mycoplasma contamination.
Commonly misidentified lines (See ICLAC register)	*Name any commonly misidentified cell lines used in the study and provide a rationale for their use.*

Plants

Seed stocks	*Report on the source of all seed stocks or other plant material used. If applicable, state the seed stock centre and catalogue number. If plant specimens were collected from the field, describe the collection location, date and sampling procedures.*
Novel plant genotypes	*Describe the methods by which all novel plant genotypes were produced. This includes those generated by transgenic approaches, gene editing, chemical/radiation-based mutagenesis and hybridization. For transgenic lines, describe the transformation method, the number of independent lines analyzed and the generation upon which experiments were performed. For gene-edited lines, describe the editor used, the endogenous sequence targeted for editing, the targeting guide RNA sequence (if applicable) and how the editor was applied.*
Authentication	*Describe any authentication procedures for each seed stock used or novel genotype generated. Describe any experiments used to assess the effect of a mutation and, where applicable, how potential secondary effects (e.g. second site T-DNA insertions, mosiacism, off-target gene editing) were examined.*
