# Supplementary material for: Structural mechanisms underlying resistance to Switch-II pocket inhibitors in KRAS
Source: Nat Commun. 2026 Sep 29;17:11229. doi: 10.1038/s41467-026-78133-4 (PMC13720030; doi:10.1038/s41467-026-78133-4)
Supplement: Supplementary file 3 [file 41467_2026_78133_MOESM3_ESM.pdf]

nature portfolio

Peer Review File

Structural mechanisms underlying resistance to Switch-II pocket inhibitors in KRAS

Corresponding Author: Dr Dhirendra Simanshu

This file contains all reviewer reports in order by version, followed by all author rebuttals in order by version.

Version 0:

Reviewer comments:

Reviewer #1

(Remarks to the Author)
NCOMMS-26-019185
Structural mechanisms underlying resistance to Switch-II pocket inhibitors in KRAS

In this manuscript, Whitley et al. characterize five of the mutations that cause resistance to KRAS G12C inhibitors. They use biophysical techniques as well as structural biology and experiments in cells. They find that mutations fall into two categories, either disrupting interactions with the drugs or occluding the pocket itself. Different drugs have different sensitivities to different mutants, and these are explained from a mechanistic perspective. Finally, different mutations have different effects on downstream signaling.

This is a solid paper, and while the results are not necessarily unexpected, they do further contribute to our understanding of this important cancer drug target. Also, the paper is clearly written. I have just a couple suggestions.

First, it would be instructive to know how often the five mutations studied actually occur in cancer patients, and how these fit into the broader landscape of KRAS resistance. Although approved KRAS inhibitors are still relatively new in clinical practice, some data exist, and the authors should state how frequently each of the five mutations occur in response to various treatments where that information is available.

Second, on page 11 the authors note that “ITC measurements report a weaker apparent affinity for KRAS G12C (KD ~9 μM), suggesting that the reduced affinity observed by ITC reflects methodological effects rather than truly impaired effector engagement.” Given the published (and referenced) SPR data, this seems like a reasonable statement, but it is strange and calls into question the rest of the ITC data. What might these “methodological effects” be, and how do we know they would not affect the other protein constructs studied?

Reviewer #2

(Remarks to the Author)
I co-reviewed this manuscript with one of the reviewers who provided the listed reports. This is part of the Nature Communications initiative to facilitate training in peer review and to provide appropriate recognition for Early Career Researchers who co-review manuscripts.

Reviewer #3

(Remarks to the Author)
In this work, Whitley et al. used a wide range of biophysical and structural techniques to examine the mechanistic consequences of KRAS Switch II pocket resistance mutations following treatment with G12C covalent inhibitors. The authors first usefully documented compound- and mutation-dependent variations in binding affinities using ITC and TS; then solved the structures of selected resistance mutations in the G12C background in complex with the inhibitors and with or without effectors Raf and PI3K using X-Ray crystallography and NMR; examined the impact of the resistance mutations on dynamics using MD simulations; and conducted cell signaling assays to assess effects of the mutations in attenuating

inhibitor effect on MAPK signaling. The combined results provided a compelling basis for proposing what the authors describe as type I and type II resistance mechanisms. This is rigorously done research and an important contribution overall. Still, there are a few issues that require attention.

1. The authors chose to focus on R68S, H95Q, Y96C, Y96D and Q99L because these are clinically documented resistance mutations. It is therefore expected that the results are discussed in relation to clinical data as this would strengthen the conclusions. Some examples to consider:
a. Is there information (such as PMID: 40293709?) about which of the five clinical resistance mutants is most or least frequent in patients who have developed resistance to G12C inhibitors? If, for example, G12C/Y96D is most frequent in patients who developed resistance to G12Ci, then the G12C/Y96D story would be more compelling.
b. Do the authors see any correlation between the covalent kinetics (Fig 2) and the overall response rates (ORR) or reported clinical outcomes for sotorasib, adagrasib, and BBO-8520 (e.g., does BBO-8520 show the best ORR in patients among the three?)
c. Given the increased covalent efficiency of sotorasib in G12C/H95Q, is this Type I resistance mutant absent or less present in patients who developed resistance to sotorasib?

2. On BBO-8520:
a. How do the kinact/KI values in Fig 2 compare with reported values (e.g., BB8520 showed almost 2x kinact/KI here than in PMID: 41790032).
b. Despite reductions in its covalent kinetics, BBO-8520 still showed remarkably better kinact/KI in the resistance mutants than those of sotorasib and adagrasib for G12C alone. It would be interesting to know how BBO-8520 affects Raf/PI3K binding in the resistance mutants.

3. The authors sought to explain the effect of the resistance mutants on covalent kinetics by focusing on the reversible binding (KI) of covalent inhibitors:
a. In pages 5-6: “Because most direct KRAS inhibitors, whether covalent or noncovalent, bind to the Switch- II pocket, noncovalent inhibitors provide a direct readout of how resistance substitutions alter pocket recognition, independent of covalent chemistry.” Since this is the premise to justify the use of the noncovalent inhibitor MRTX1133 as a surrogate for KI in the covalent ones, can the authors present data or reference(s) for this observation (for instance reports on binding or activity of MRTX1133 in G12C or non-G12D mutants)? Similarly, data or reference(s) are missing for “although developed as a KRAS G12D inhibitor, MRTX1133 also binds KRAS G12C with high affinity, and is among the most structurally and biochemically well-characterized noncovalent Switch-II pocket inhibitors.” (page 6)
b. Presenting this said premise without data or reference implies the following assumptions that the authors did not establish first: (1) Focusing on reversible binding (KI) to explain the effect on covalent kinetics in resistance mutants assumes that all three inhibitors have the same kinact. Were they the same? (2) While the authors reported kinact/KI, the individual KI values for the covalent inhibitors in Fig 2 could be different. Does the effect of the resistance mutants on KD of MRTX1133 represent the effect on irreversible binding for all the covalent inhibitors (Figure 3A), especially since it was as noted that they expect the covalent inhibitors to have different Switch II engagements? Is there similar data for the BBO-11818/BBO-8520 and BI0474/BI2865 covalent/noncovalent pairs? Without these data, it would seem that MRTX1133 as a surrogate is most reliable only for adagrasib as its parent compound (e.g., KI = 3.7 uM in PMID: 32250617). (3) Related to the above “These findings suggest that disruption of reversible inhibitor binding is the major contributor to resistance conferred by mutations at these sites and provide a mechanistic basis for the differential kinetic defects observed with the covalent KRAS G12C inhibitors (Fig. 2)” seems a strong claim backed by evidence from only one noncovalent/covalent inhibitor pair (MRTX1133/adagrasib).

4. In page 7: “Superposition with sotorasib-bound G12C-GDP (Fig. 4c) shows that the repositioned Switch-II segment directly overlaps the space occupied by the inhibitor. This structural occlusion explains both the substantial loss of covalent engagement for BBO-8520 and unmeasurable engagement for sotorasib and adagrasib (Fig. 2), as well as the ~600-fold reduction in MRTX1133 affinity relative to G12C (Fig. 3e). Since the authors already established inhibitor- and mutant-specific differences in Switch II pocket engagement, this statement requires separate superimposed structures for BBO8520, adagrasib, and MRTX1133.

5. The authors made considerable effort to resolve crystal structures of all G12C resistance mutants that will benefit the field. In these structures, they showed that the fully open Switch I conformation is unique only to the KRAS G12C/Y96D (Fig. 4), i.e., all other mutants preserved Switch I conformation (Fig. 5). While the authors provided confirmatory evidence of the Switch I fully open conformation in G12C/Y96D, little is said about any relationship between this and the Y96D-induced Switch II pocket occlusion. A comment on whether the Y96D mutation contributed in any way to this Switch I fully open conformation would be helpful. And given the improved binding of G12C/Y96D to Raf1 than G12C (Fig 6B), is it plausible that the Switch I fully open conformation in its GDP-state somehow better facilitates activation and more effective Raf1 binding?

6. If G12C/Y96D exhibits both Type I and Type II resistance and the worst effect on covalent kinetics of sotorasib and adagrasib, why are the pERK levels higher for sotorasib in G12C/R68S than Y96D and for adagrasib in G12C/R68S and G12C/H95Q than Y96D (Fig 7)? Can the authors comment on why G12C/H95Q showed lower pERK levels with sotorasib despite a similar or greater covalent engagement efficiency than G12C alone (Fig 2)?

Minor comments:

a) In Figures 2D-F, the authors may want to consider using the same y-axis scale (with a break) so that it is easier to appreciate differences in kinact/KI (both baseline and mutant covalent kinetics) across the 3 inhibitors.

b) Figure 3A: Considering that the authors are explaining the covalent kinetics data in GDP-bound KRAS through noncovalent binding (see comment #3), is there a particular reason why they used the GTP-bound MRTX1133 in Fig 3A modeling and GDP-bound KRAS in 3B and 3C? For better clarity, can the authors also specify in Figure 3 caption and results if the KRAS used was GDP-bound?

c) In Figure 3D, does DFS typically require this high concentration of inhibitor relative to target to detect effect on melting temperature? Are the differences between control and MRTX1133-bound proteins statistically significant?

d) Figure 4B: Consider including the 2.7Å vs >6 Å for H-bond comparison in the figure itself or in the caption.

e) In page 7, "The fully open Switch-I conformation observed in G12C/Y96D has been reported in multiple GDP-bound KRAS structures and is consistently associated with crystallization in space group P321 with similar unit cell parameters, suggesting that this lattice is diagnostic of the open state. As in prior examples…" Please mention "Supplementary Figure 7" in these earlier statements to reference these results.

f) How does the G12C/Y96D effect in GTP-KRAS relate to a reported off-state G12Ci escape/bypass into GTP-bound fraction (PMID: 39215000)?

g) On page 14, the authors state that Y96D preserves near-native binding to both Raf1 and PI3Kα. This is true for PI3Kα (Figure 6E) but affinity to Raf1 is much higher (>10 x). The Kd for G12C and Raf-RBD is 9.4 µM while that for G12C-Y96D is 0.315 µM. It is also interesting, but a bit confusing, that the mutants (except Q99L) have better binding affinities to Raf1.

Reviewer #4

(Remarks to the Author)
This manuscript provides a comprehensive structural and mechanistic analysis of resistance to KRAS G12C Switch-II pocket inhibitors in KRAS G12C. Using a combination of crystallography, NMR, molecular dynamics, biochemical assays, and cell signaling data, the authors define two major resistance mechanisms: (1) loss of key inhibitor interactions (Type I mutants) and (2) conformational rearrangement and increased dynamics of the Switch-II pocket (Type II mutants). These mutations reduce both covalent and noncovalent inhibitor engagement while largely preserving downstream MAPK and AKT signaling. The study offers valuable guidance for next-generation inhibitor design. Major strengths include the integration of structural, biophysical, and cellular data, classification of resistance mechanisms, and meaningful implications for drug design strategies. However, there exist concerns and points for clarification.

1. Mutant selection and scope.

The frequencies of clinically observed secondary mutations should be provided. Moreover, the mutations studied are biased toward adagrasib resistance. Hence, inclusion or discussion of sotorasib-specific resistance mutations would strengthen conclusions. The rationale for testing G12D inhibitors and G12D-associated resistance mutations in a G12C context should be clarified.

2. Structural analyses/interpretation.

Additional consideration/analyses are needed for potential warhead misalignment vs. affinity-driven resistance. Please state whether structural evidence suggests a "warhead misalignment" in any of the mutants.

The study suggests that "distributing binding interactions" across the pocket can mitigate Type I resistance. Could the authors suggest which specific residues within the Switch-II area are most "mutation-proof" based on their structural data? Identification of "mutation-resistant" residues for drug design may aid in future drug discovery efforts.

Type II resistance appears to rely on a non-native D96-T58 H-bond. From MD analysis, does the mutation trap a "structural water" that further prevents drug entry? The discovery of the nucleotide-dependent intramolecular interactions (D96-T58 in GDP-bound vs. D96-R68 in GTP-bound) is intriguing. A comparison of intramolecular interactions (e.g., D96-R68 vs. R68-E37 competition) and their impact on Switch-I stability should be included.

The authors observe a unique phenomenon where the H95Q mutation slightly enhances the covalent engagement of sotorasib (kinact/KI increase from 10,800 to 14,400 M-1s-1), whereas it severely impairs adagrasib and BBO-8520. A structural overlay of the sotorasib-bound model with the H95Q mutant structure will aid in visualization of whether this preference is driven by the relief of a specific van der Waals clash or the formation of a new polar network.

3. ITC data.
The affinity for RAF RBD with KRAS G12C appears low compared to previous reports (e.g. Alexander et al, JBC (2025) by the RAS NCI group). It would be helpful to compare ITC-derived affinities (e.g., KRAS–RAF) with the literature for consistency and whether the constructs differ. The heat of dilution is not subtracted in many Wiseman plots with some

datasets appearing truncated or poorly fit. A subset of the figures provides replicates (Fig 3, 6) while others do not (Fig 2, 7). In Fig 1 I , the labeling of G12C-GDP/GTP is misleading, considering the structure shown is of GDP. In Fig 3E , the heat of dilution for the 1st one is subtracted, while the other 5 are not. For Fig 6 B and E, should the Wiseman plots start at the same point when the plateau doesn't go to zero (heat of dilution not subtracted in select plots, specifically E). In SI Fig 3, only the 1st one is correct, which is the same one shown in Fig 3E. In SI Fig 8, the grey replicate for the G12C-RAF1 is truncated and linear, neither fit shows a plateau. In SI Fig 10, several replicates do not have heat of dilution subtracted. dH values are not trustworthy when heats of dilution have not been subtracted. The biphasic binding behavior (e.g., Q99L) may indicate aggregation or multiple binding modes and should be investigated.

4. Methodology.

Methodological details are lacking for the DSF fluorophore, statistical reporting, and replicate consistency.

MD simulation convergence (e.g., RMSD) should be shown to support conclusions. The authors performed 5 x 1 µs simulations for all variants. More details regarding the convergence of these simulations (e.g., RMSD plots) to ensure the observed populations of "occluded" states are statistically robust are needed.

Error quantification is needed for the cell signaling data in the absence and presence of the inhibitors.

5. NMR data.

HSQC data suggest possible conformational heterogeneity in certain mutants. Some figure labeling and presentation require correction for clarity. For example, the HSQC spectrum for G12C/Y96D-GDP shows a potential 2nd conformation from the population of weak peaks.

6. Cell signaling.

Error quantification is lacking for the signaling assays, making it difficult to interpret the data presented. While RAS resistance mutants appear to maintain MAPK signaling in the presence of RAS inhibitors, AKT effects are weaker and require further discussion. It is unclear whether limited pAKT suppression may reflect pathway redundancy or cell-line-specific biology rather than KRAS effects alone. It is also unclear whether the RAS resistance mutants studied alter signaling in the absence of inhibitors.

In summary, this study represents a strong and impactful study that advances understanding of KRAS inhibitor resistance. However, issues related to data analysis, methodological details, and mechanistic interpretation should be addressed to strengthen confidence in the conclusions.

Reviewer #5

(Remarks to the Author)
I co-reviewed this manuscript with one of the reviewers who provided the listed reports. This is part of the Nature Communications initiative to facilitate training in peer review and to provide appropriate recognition for Early Career Researchers who co-review manuscripts.

Version 1:

Reviewer comments:

Reviewer #1

(Remarks to the Author)
In my opinion, the authors have adequately addressed my concerns and the manuscript is now suitable for publication.

Reviewer #2

(Remarks to the Author)
I co-reviewed this manuscript with one of the reviewers who provided the listed reports. This is part of the Nature Communications initiative to facilitate training in peer review and to provide appropriate recognition for Early Career Researchers who co-review manuscripts.

Reviewer #3

(Remarks to the Author)
The authors have made admirable effort in addressing reviewers' concerns, and the revision and additional data have considerably strengthened the manuscript. Just a few minor issues remain:

• In Introduction (pages 2-4) and elsewhere, it would help to be consistent if the justification for the choice of resistance mutations (especially the non-Y96D) was based on “most recurrent” (i.e., occurring in different studies) or “most common” (which denotes mutational prevalence). The two terms are different but appeared to be used interchangeably.

• In the rebuttal the authors stated, “Structural studies additionally show that the MRTX1133 binding pose is highly conserved across KRAS alleles and closely resembles the pre-covalent binding mode of clinical KRAS G12C inhibitors” (page 4). This is an important justification that should be included in the results (with references if possible), especially since they recognized that MRTX1133 “does not fully recapitulate the binding properties of all covalent inhibitors” (page 7).

• Clarity (based on the overall MRTX1133 data) if disruption of Switch II (or p2; btw please acknowledge and cite papers that characterized the pocket before it was renamed) is “a major” (rebuttal, pages 7-8) or “the major” (results, page 7) contributor to resistance conferred by the resistance mutations.

• Do the authors believe the “Type I/Type II resistance” classification might be applicable to other prevalent G12X mutants?

Reviewer #4

(Remarks to the Author)
In the revised manuscript, the authors have addressed the majority of the concerns raised in the original review. However, a few minor issues should still be addressed before publication:

The authors state that Figure 3A has been updated to show GDP-bound KRAS. However, the figure remains labeled as GMPPCP, and it does not appear that the figure has been revised accordingly. The authors should ensure that the figure and its labeling accurately reflect the intended nucleotide state.

The authors propose that the Y96D mutation adopts a fully open Switch-I conformation that is independent of crystal lattice contacts, stating that "Widespread chemical shift perturbations, including residues in Switch-I and Switch-II, indicate that the observed conformational rearrangements are not solely crystal lattice-induced...". However, the NMR data presented in Supplementary Figure 7 do not appear to fully support this conclusion. Only modest chemical shift perturbations are observed for residues within the Switch-I region, whereas more pronounced effects, including evidence of multiple conformational populations, are primarily observed for residues proximal to the nucleotide-binding motifs. The authors should either provide additional evidence supporting extensive Switch-I rearrangement in solution or temper their interpretation to better reflect the experimental data

Open Access This Peer Review File is licensed under a Creative Commons Attribution 4.0 International License, which permits use, sharing, adaptation, distribution and reproduction in any medium or format, as long as you give appropriate credit to the original author(s) and the source, provide a link to the Creative Commons license, and indicate if changes were made.
In cases where reviewers are anonymous, credit should be given to 'Anonymous Referee' and the source.
The images or other third party material in this Peer Review File are included in the article’s Creative Commons license,

unless indicated otherwise in a credit line to the material. If material is not included in the article's Creative Commons license and your intended use is not permitted by statutory regulation or exceeds the permitted use, you will need to obtain permission directly from the copyright holder.
To view a copy of this license, visit https://creativecommons.org/licenses/by/4.0/

Point-by-point response to reviewer's comments:

We appreciate the reviewers' thorough and constructive feedback. In this revised manuscript, we have addressed all concerns raised, resulting in an improved and more robust version. Below, we provide point-by-point responses to the reviewers' comments (highlighted in green).

Reviewer #1:

In this manuscript, Whitley et al. characterize five of the mutations that cause resistance to KRAS G12C inhibitors. They use biophysical techniques as well as structural biology and experiments in cells. They find that mutations fall into two categories, either disrupting interactions with the drugs or occluding the pocket itself. Different drugs have different sensitivities to different mutants, and these are explained from a mechanistic perspective. Finally, different mutations have different effects on downstream signaling. This is a solid paper, and while the results are not necessarily unexpected, they do further contribute to our understanding of this important cancer drug target. Also, the paper is clearly written. I have just a couple suggestions.

First, it would be instructive to know how often the five mutations studied actually occur in cancer patients, and how these fit into the broader landscape of KRAS resistance. Although approved KRAS inhibitors are still relatively new in clinical practice, some data exist, and the authors should state how frequently each of the five mutations occur in response to various treatments where that information is available.

Response: We thank the reviewer for this suggestion and have added a paragraph to the Introduction addressing the clinical prevalence of these mutations. We note that precise per-mutation frequencies are not currently available. Available clinical datasets remain relatively limited, and most studies have focused on identifying resistance mechanisms rather than establishing mutation prevalence. In addition, many patients develop heterogeneous resistance involving multiple concurrent alterations, making it difficult to determine the contribution of individual mutations.

Within these limitations, the available evidence indicates that:

- Y96D is the most frequently observed clinically acquired Switch-II pocket mutation and was the most common mutation in this class in a 143-patient post-progression cohort in which Switch-II pocket mutations emerged in 14% of patients overall (Riedl et al., 2025).
- R68S, H95Q, and Y96C were first identified in adagrasib-treated patients (Awad et al., 2021), each occurring in individual patients within a 38-patient cohort.

- Q99L appears to be less common clinically but was the dominant secondary mutation identified in adagrasib-resistant Ba/F3 models (Koga et al., 2021) and has subsequently been reported in patients.

These observations place the mutations examined in our study within the broader landscape of clinically acquired resistance to KRAS inhibition. Importantly, our findings provide mechanistic insight into both the most prevalent mutation in this class (Y96D) and several less common but recurrent resistance mutations that alter inhibitor engagement through distinct structural mechanisms.

We have now included this information in the revised Introduction as follows:

"*Several clinically acquired resistance mutations have been identified within the KRAS Switch-II pocket following treatment with KRAS(G12C) inhibitors. Among these, Y96D is the most frequently observed mutation, representing the most common Switch-II pocket substitution in a 143-patient post-progression cohort in which Switch-II pocket mutations emerged in 14% of patients overall (Riedl et al., 2025). Additional recurrent mutations, including R68S, H95Q, Y96C, and Q99L, have also been reported in patients or preclinical resistance models (Awad et al., 2021; Koga et al., 2021).*"

Second, on page 11 the authors note that "ITC measurements report a weaker apparent affinity for KRAS G12C (KD ~9 μM), suggesting that the reduced affinity observed by ITC reflects methodological effects rather than truly impaired effector engagement." Given the published (and referenced) SPR data, this seems like a reasonable statement, but it is strange and calls into question the rest of the ITC data. What might these "methodological effects" be, and how do we know they would not affect the other protein constructs studied?

Response: As the reviewer noted, the apparent affinity of KRAS G12C for RAF1-RBD measured by ITC was weaker than expected based on previously reported SPR measurements and earlier ITC experiments. In our earlier measurements, the titration curves did not extend sufficiently into the saturation regime to tightly constrain the fit, resulting in a relatively large uncertainty in the fitted KD (~9 ± 5 μM). To address this issue, we repeated the experiments using optimized protein concentration ratios designed to produce more complete binding isotherms and improve curve fitting. Under these optimized conditions, the measured affinity was substantially better constrained, yielding a K_D of ~2 μM, which is close to the affinities reported for other activated KRAS mutants. Figure 6B has been updated with the optimized ITC experiments for G12C with the RAF1-RBD.

At the same time, we cannot completely rule out methodological limitations inherent to ITC measurements for this interaction. For this reason, we included an orthogonal analytical gel filtration assay to directly assess complex formation independent of

calorimetric fitting. Importantly, these experiments confirmed stable complex formation between RAF1-RBD and all resistance mutants examined. Thus, our primary conclusion is that the resistance mutants retain the ability to engage RAF1-RBD, even though the precise absolute K_D values may vary somewhat depending on the experimental method and conditions.

Reviewer #3:

In this work, Whitley et al. used a wide range of biophysical and structural techniques to examine the mechanistic consequences of KRAS Switch II pocket resistance mutations following treatment with G12C covalent inhibitors. The authors first usefully documented compound- and mutation-dependent variations in binding affinities using ITC and TS; then solved the structures of selected resistance mutations in the G12C background in complex with the inhibitors and with or without effectors Raf and PI3K using X-Ray crystallography and NMR; examined the impact of the resistance mutations on dynamics using MD simulations; and conducted cell signaling assays to assess effects of the mutations in attenuating inhibitor effect on MAPK signaling. The combined results provided a compelling basis for proposing what the authors describe as type I and type II resistance mechanisms. This is rigorously done research and an important contribution overall. Still, there are a few issues that require attention.

1. The authors chose to focus on R68S, H95Q, Y96C, Y96D and Q99L because these are clinically documented resistance mutations. It is therefore expected that the results are discussed in relation to clinical data as this would strengthen the conclusions. Some examples to consider:

a. Is there information (such as PMID: 40293709?) about which of the five clinical resistance mutants is most or least frequent in patients who have developed resistance to G12C inhibitors? If, for example, G12C/Y96D is most frequent in patients who developed resistance to G12Ci, then the G12C/Y96D story would be more compelling.

Response: We thank the reviewer for highlighting the recent review by Ebright et al. (2025, *Cancer Discovery*; PMID: 40293709). This study provides comprehensive reviews of clinical and preclinical mechanisms of resistance to RAS inhibitors, integrating data from multiple studies on acquired resistance to KRAS(G12C) inhibitors. Importantly, the authors note that resistance is highly heterogeneous and that many patients develop multiple concurrent resistance alterations, making precise determination of individual mutation frequencies challenging.

Within these limitations, the available evidence indicates that Y96D is the most frequently observed clinically acquired Switch-II pocket mutation, representing the most common mutation in this class in a 143-patient post-progression cohort in which Switch-II

pocket mutations emerged in 14% of patients overall (Riedl et al., 2025, ref. 5). In contrast, R68S, H95Q, Y96C, and Q99L have been reported less frequently, with Q99L initially identified as the dominant secondary mutation in adagrasib-resistance Ba/F3 models before subsequently being observed clinically.

These observations place the mutations examined in our study within the broader landscape of clinically acquired resistance to KRAS inhibition and further highlight the importance of understanding the structural basis of resistance associated with Y96D, the most prevalent mutation in this class. We have now added the following text to the revised Introduction:

"*Several clinically acquired resistance mutations have been identified within the KRAS Switch-II pocket following treatment with KRAS(G12C) inhibitors. Among these, Y96D is the most frequently observed mutation, representing the most common Switch-II pocket substitution in a 143-patient post-progression cohort in which Switch-II pocket mutations emerged in 14% of patients overall (Riedl et al., 2025). Additional recurrent mutations, including R68S, H95Q, Y96C, and Q99L, have also been reported in patients or preclinical resistance models (Awad et al., 2021; Koga et al., 2021).*"

b. Do the authors see any correlation between the covalent kinetics (Fig 2) and the overall response rates (ORR) or reported clinical outcomes for sotorasib, adagrasib, and BBO-8520 (e.g., does BBO-8520 show the best ORR in patients among the three?).

Response: We thank the reviewer for this insightful question. The kinact/KI values measured in this study for KRAS G12C, 10,800 $M^{-1}s^{-1}$ for sotorasib, 175,000 $M^{-1}s^{-1}$ for adagrasib, and 4,792,000 $M^{-1}s^{-1}$ for BBO-8520 (Fig. 2) are directionally consistent with the rank order of clinical ORRs reported across available trials, though a direct quantitative correlation cannot be established given the different trial contexts and patient populations.

Sotorasib demonstrated an ORR of 37.1% in Phase II CodeBreaK 100 (n = 126; Skoulidis et al., N Engl J Med, 2021) and 28.1% in Phase III CodeBreaK 200 (de Langen et al., Lancet, 2023). Adagrasib showed an ORR of 42.9% in Phase II KRYSTAL-1 (n = 112 evaluable; Jänne et al., N Engl J Med, 2022) and 32% in Phase III KRYSTAL-12 (Mok et al., J Clin Oncol, 2024). Despite the ~16-fold difference in kinact/KI between sotorasib and adagrasib, a matching-adjusted indirect comparison using Phase III data found comparable overall efficacy between the two agents (Chopra et al., Adv Ther, 2025; 42:4300–4317). This is mechanistically consistent with their shared fundamental limitation of engaging only the GDP-bound, inactive KRAS state, which dominates clinical resistance regardless of kinetic differences within that state.

BBO-8520 (kinact/KI = 4,792,000 $M^{-1}s^{-1}$), whose ~440-fold kinetic advantage over adagrasib is further extended by its capacity to engage GTP-bound KRAS G12C, showed a

preliminary ORR of 65% (11/17 patients; confirmed ~58% excluding one unconfirmed response) in the Phase 1 ONKORAS-101 trial as of November 2025 (BBOT Press Release, January 7, 2026; NCT06343402). These early data are directionally consistent with the hypothesis that higher intrinsic covalent engagement efficiency, combined with dual-state inhibition, translates to improved clinical response, an observation also reflected in the cellular signaling data of Figure 7, where BBO-8520 retains meaningful pERK suppression at 10–100 nM across all resistance mutants while sotorasib and adagrasib lose most activity at concentrations up to 1,000 nM. We note that the BBO-8520 clinical data are preliminary (n = 17, Phase 1, spanning multiple dose levels), and confirmatory Phase 2/3 data will be required to establish whether these advantages translate to durable improvements in ORR and PFS.

We have added pertinent points summarizing these issues to the Discussion section of the manuscript.

c. Given the increased covalent efficiency of sotorasib in G12C/H95Q, is this Type I resistance mutant absent or less present in patients who developed resistance to sotorasib?

Response: Unlike adagrasib, relatively limited clinical data are currently available regarding acquired Switch-II pocket resistance mutations in sotorasib-treated patients, making it difficult to draw robust mutation-specific frequency trends at present. However, the available evidence is consistent with our biochemical findings that H95 substitutions are not associated with resistance to sotorasib. Awad et al. (*NEJM*, 2021) identified acquired H95D/Q/R mutations in adagrasib-resistant patients and experimentally demonstrated using engineered Ba/F3 cell models that, although these mutations conferred marked resistance to adagrasib, "the G12C/H95D, G12C/H95Q, and G12C/H95R mutants remained sensitive to sotorasib." More recently, Riedl et al. (*Annals of Oncology,* 2025) further showed that KRAS H95L conferred complete resistance to adagrasib and divarasib while remaining highly sensitive to sotorasib, and notably reported that all clinically observed H95 resistance events in their cohort emerged in patients treated with adagrasib or divarasib rather than sotorasib. These findings are consistent with our structural and kinetic data, which show that sotorasib has comparatively fewer interactions with H95 than adagrasib and therefore retains activity against the H95Q mutant.

2. On BBO-8520:

a. How do the kinact/KI values in Fig 2 compare with reported values (e.g., BB8520 showed almost 2x kinact/KI here than in PMID: 41790032).

Response: As the reviewer noted, the kinact/KI value reported here for BBO-8520 is somewhat higher than previously reported (PMID: 41790032). In the present study, these values were derived from additional data points collected under conditions optimized for this inhibitor, including expanded time-course measurements and lower protein concentrations for KRAS G12C to better resolve the rapid covalent engagement kinetics. Importantly, however, the overall magnitude, mutation-dependent trends, and relative behavior of BBO-8520 compared with other KRAS G12C inhibitors remain largely consistent with the prior report. We have added clarifying text to the manuscript.

b. Despite reductions in its covalent kinetics, BBO-8520 still showed remarkably better kinact/KI in the resistance mutants than those of sotorasib and adagrasib for G12C alone. It would be interesting to know how BBO-8520 affects Raf/PI3K binding in the resistance mutants.

Response: Direct measurements of RAF1 and PI3Kα binding to BBO-8520-labeled resistance mutants could, in principle, provide additional mechanistic insight. However, the cellular signaling experiments included in the original submission (Fig. 7 and Supplementary Fig. 12) were specifically designed to assess the functional consequences of inhibitor engagement on downstream MAPK- and PI3K-dependent signaling across the resistance mutants in a cellular context. We also anticipate substantial technical limitations in directly measuring effector binding to BBO-8520-labeled resistance mutants. While mass spectrometry can quantify the extent of covalent modification, it cannot establish whether the inhibitor remains productively engaged within the Switch-II pocket after labeling. This issue is further complicated by the substantially different covalent engagement efficiencies observed across the resistance mutants, which make it difficult to achieve comparable levels of inhibitor labeling under a common set of experimental conditions. Consequently, differences in measured effector binding could reflect varying extents of labeling, heterogeneous inhibitor-bound states, and mutation-dependent conformational effects, making mechanistic interpretation ambiguous. The cellular signaling data therefore provide a more biologically relevant assessment of how resistance mutations influence downstream RAF1- and PI3Kα-dependent signaling in the presence of these inhibitors.

3. The authors sought to explain the effect of the resistance mutants on covalent kinetics by focusing on the reversible binding (KI) of covalent inhibitors:

a. In pages 5-6: "Because most direct KRAS inhibitors, whether covalent or noncovalent, bind to the Switch- II pocket, noncovalent inhibitors provide a direct readout of how resistance substitutions alter pocket recognition, independent of covalent chemistry." Since this is the premise to justify the use of the noncovalent inhibitor MRTX1133 as a surrogate

for KI in the covalent ones, can the authors present data or reference(s) for this observation (for instance, reports on binding or activity of MRTX1133 in G12C or non-G12D mutants)? Similarly, data or reference(s) are missing for “although developed as a KRAS G12D inhibitor, MRTX1133 also binds KRAS G12C with high affinity, and is among the most structurally and biochemically well-characterized noncovalent Switch-II pocket inhibitors.” (page 6).

Response: We thank the reviewer for this important point. Our rationale for using MRTX1133 was not that it quantitatively represents the K_I of all covalent inhibitors, but rather that it serves as a well-characterized noncovalent probe of Switch-II pocket recognition independent of covalent chemistry. Because productive engagement by all Switch-II pocket inhibitors requires an initial reversible binding step, noncovalent binding measurements provide a direct readout of how resistance substitutions perturb pocket recognition. Although developed as a KRAS G12D inhibitor, MRTX1133 has been shown to bind multiple KRAS variants, including KRAS G12C and WT KRAS, with high affinity via a conserved Switch-II pocket-binding mode. In our study, KRAS G12C bound MRTX1133 with a K_D of ~43 nM. Alexander et al. (J Biol Chem 2025) further showed that MRTX1133 affinity varies by less than twofold across several GDP-loaded KRAS mutants, including G12C, G12V, G13D, Q61H, and A146T. Structural studies additionally show that the MRTX1133 binding pose is highly conserved across KRAS alleles and closely resembles the pre-covalent binding mode of clinical KRAS G12C inhibitors. Importantly, the mutation-dependent trends we observe with MRTX1133 closely parallel the covalent engagement defects observed across multiple inhibitor scaffolds, supporting the conclusion that disruption of Switch-II pocket recognition is a major contributor to resistance conferred by these mutations. We have updated the manuscript to clarify that MRTX1133 binds multiple KRAS-GDP mutants with high affinity.

b. Presenting this said premise without data or reference implies the following assumptions that the authors did not establish first:

(1) Focusing on reversible binding (KI) to explain the effect on covalent kinetics in resistance mutants assumes that all three inhibitors have the same kinact. Were they the same?

Response: We thank the reviewer for this important question. Our interpretation does not assume that k_{inact} is identical across inhibitors or mutants. Rather, we propose that the dominant effect of these resistance mutations is on the reversible binding step (KI), based on both structural and biochemical considerations. The mutated residues are distal to C12 and primarily affect the geometry and accessibility of the Switch-II pocket, which is expected to impair formation of the initial noncovalent encounter complex.

While we cannot exclude the possibility that k_{inact} may also be affected in some cases, the consistent reduction or loss of engagement across structurally distinct inhibitors, together with the MRTX1133 noncovalent binding data, supports disruption of the

noncovalent binding step as the primary driver. We have revised the manuscript to clarify this point and to avoid implicitly implying that k_{inact} is unchanged.

(2) While the authors reported kinact/KI, the individual KI values for the covalent inhibitors in Fig 2 could be different. Does the effect of the resistance mutants on KD of MRTX1133 represent the effect on irreversible binding for all the covalent inhibitors (Figure 3A), especially since it was as noted that they expect the covalent inhibitors to have different Switch II engagements? Is there similar data for the BBO-11818/BBO-8520 and BI0474/BI2865 covalent/noncovalent pairs? Without these data, it would seem that MRTX1133 as a surrogate is most reliable only for adagrasib as its parent compound (e.g., KI = 3.7 uM in PMID: 32250617).

Response: We thank the reviewer for this insightful comment. We agree that individual covalent inhibitors exhibit distinct binding modes within the Switch-II pocket and may therefore differ in their sensitivity to specific resistance mutations. Our intention in using MRTX1133 was not to imply that it quantitatively represents the KI of all covalent inhibitors, but rather to provide a general readout of how mutations perturb Switch-II pocket recognition.

Importantly, we observe consistent mutation-dependent trends between the noncovalent binding data (MRTX1133) and covalent engagement kinetics across multiple inhibitors, with Y96D showing the strongest impairment, followed by H95Q/Y96C and then R68S/Q99L. This concordance supports the conclusion that disruption of the reversible binding step is a major contributor to reduced covalent engagement.

We agree that matched covalent/noncovalent inhibitor pairs (e.g., BBO-8520/BBO-11818 or BI-0474/BI-2865) would provide additional insight into scaffold-specific effects. However, such datasets are not currently available for all systems examined here. We have revised the manuscript to clarify that MRTX1133 serves as a representative probe of pocket recognition rather than a universal surrogate for all covalent inhibitors.

(3) Related to the above "These findings suggest that disruption of reversible inhibitor binding is the major contributor to resistance conferred by mutations at these sites and provide a mechanistic basis for the differential kinetic defects observed with the covalent KRAS G12C inhibitors (Fig. 2)" seems a strong claim backed by evidence from only one noncovalent/covalent inhibitor pair (MRTX1133/adagrasib).

Response: We thank the reviewer for this important point. We agree that our original wording was too strong, given the limited number of direct noncovalent–covalent comparisons. We have therefore revised the statement to clarify that disruption of reversible binding is an

important contributor to resistance, rather than a universally established mechanism across all inhibitor chemotypes. In particular, the noncovalent–covalent comparison using the MRTX1133/adagrasib pair provides direct support for this model, while the consistency of mutation-dependent trends across multiple inhibitors suggests that this mechanism is broadly applicable, albeit with inhibitor-specific differences. The manuscript has been revised accordingly.

4. In page 7: "Superposition with sotorasib-bound G12C-GDP (Fig. 4c) shows that the repositioned Switch-II segment directly overlaps the space occupied by the inhibitor. This structural occlusion explains both the substantial loss of covalent engagement for BBO-8520 and unmeasurable engagement for sotorasib and adagrasib (Fig. 2), as well as the ~600-fold reduction in MRTX1133 affinity relative to G12C (Fig. 3e)." Since the authors already established inhibitor- and mutant-specific differences in Switch II pocket engagement, this statement requires separate superimposed structures for BBO8520, adagrasib, and MRTX1133.

Response: As suggested, we have performed new structural superpositions of our experimental G12C/Y96D-GDP structure with structures containing covalently bound (i) adagrasib, (ii) BBO-8520, and (iii) non-covalently bound MRTX1133; these superpositions are included in the newly created Supplementary Figure 6. The figures demonstrate that the observed pocket occlusion created by the non-native D96-T58 hydrogen bond is broadly incompatible with both covalent and noncovalent Switch-II pocket inhibitors. The main text has been updated to indicate this.

5. The authors made considerable effort to resolve crystal structures of all G12C resistance mutants that will benefit the field. In these structures, they showed that the fully open Switch I conformation is unique only to the KRAS G12C/Y96D (Fig. 4), i.e., all other mutants preserved Switch I conformation (Fig. 5). While the authors provided confirmatory evidence of the Switch I fully open conformation in G12C/Y96D, little is said about any relationship between this and the Y96D-induced Switch II pocket occlusion. A comment on whether the Y96D mutation contributed in any way to this Switch I fully open conformation would be helpful. And given the improved binding of G12C/Y96D to Raf1 than G12C (Fig 6B), is it plausible that the Switch I fully open conformation in its GDP-state somehow better facilitates activation and more effective Raf1 binding?

Response: We thank the reviewer for these questions. The fully open Switch-I conformation observed in the GDP-bound G12C/Y96D structure is not unique to this mutant and has previously been observed in multiple KRAS-GDP structures spanning a range of different KRAS constructs and mutants that all crystallized in the same lattice environment.

Importantly, however, the Switch-II arrangement in G12C/Y96D is distinct from those previously reported. Specifically, the non-native D96–T58 hydrogen bond draws the Switch-II backbone into the Switch-II pocket, partially occluding the inhibitor-binding cavity in a manner not observed in other fully open Switch-I KRAS structures. The preserved RAF1 binding observed for Y96D is therefore unlikely to arise from the fully open GDP-bound conformation itself. Instead, both the co-crystal structure of G12C/Y96D-GMPPNP bound to RAF1 RBD-CRD and the biochemical binding data indicate that the mutation remains fully compatible with an effector-binding active-state architecture. In the GMPPNP-bound structure, the D96–T58 interaction is absent, and Switch-II adopts a conformation highly similar to the WT KRAS–RAF1 complex (backbone RMSD 0.22 Å). We therefore favor the interpretation that the enhanced apparent RAF1 affinity reflects altered active-state conformational or dynamic properties associated with the Y96D mutation rather than the fully open GDP-bound state.

6. If G12C/Y96D exhibits both Type I and Type II resistance and the worst effect on covalent kinetics of sotorasib and adagrasib, why are the pERK levels higher for sotorasib in G12C/R68S than Y96D and for adagrasib in G12C/R68S and G12C/H95Q than Y96D (Fig 7)? Can the authors comment on why G12C/H95Q showed lower pERK levels with sotorasib despite a similar or greater covalent engagement efficiency than G12C alone (Fig 2)?

Response: We thank the reviewer for highlighting this important point. While Y96D exhibits the strongest impairment in covalent engagement, cellular MAPK output does not scale linearly with k_{inact}/K_I. As also noted in the manuscript, pERK reflects the net level of active KRAS signaling after drug treatment and is influenced by multiple factors, including baseline inhibitor potency, intracellular drug exposure, KRAS nucleotide-state distribution, and the nonlinear relationship between fractional KRAS occupancy and MAPK pathway activation.

In this context, the observation that R68S can display higher residual pERK levels than Y96D, despite less severe biochemical impairment, likely reflects mutation-specific effects on KRAS conformational dynamics and signaling output. R68S perturbs the electrostatic and structural framework of the Switch-II/helix α3 interface and increases Switch-region flexibility, potentially altering the population of signaling-competent conformations or biasing signaling toward the MAPK pathway. In contrast, Y96D primarily confers resistance through pocket occlusion without strongly enhancing intrinsic signaling output, resulting in comparatively lower pERK suppression despite more severe impairment of inhibitor binding.

The behavior of H95Q further supports the importance of inhibitor-specific interactions. Sotorasib makes relatively limited contacts with H95 and retains efficient engagement in this mutant, resulting in effective MAPK suppression despite comparable or

slightly improved covalent engagement kinetics. In contrast, inhibitors that rely more strongly on H95 interactions are more sensitive to this substitution.

We have revised the manuscript to explicitly clarify that biochemical engagement and cellular signaling output are not directly proportional and to provide mutation-specific interpretations that reconcile the observed differences between kinetic and cellular data.

Minor comments:

a) In Figures 2D-F, the authors may want to consider using the same y-axis scale (with a break) so that it is easier to appreciate differences in kinact/KI (both baseline and mutant covalent kinetics) across the 3 inhibitors.

Response: We thank the reviewer for this suggestion and have attempted to use a common y-axis scale. However, because the k_{inact}/K_I values span several orders of magnitude across the three inhibitors, a common y-axis scale was not practical. Instead, we have revised Figures 2D–F to incorporate axis breaks, which improve visualization of the mutation-dependent effects while maintaining clarity. Additionally, the figure legend draws the reader's attention to the different y-axis scales.

b) Figure 3A: Considering that the authors are explaining the covalent kinetics data in GDP-bound KRAS through noncovalent binding (see comment #3), is there a particular reason why they used the GTP-bound MRTX1133 in Fig 3A modeling and GDP-bound KRAS in 3B and 3C? For better clarity, can the authors also specify in Figure 3 caption and results if the KRAS used was GDP-bound?

Response: We thank the reviewer for pointing out the confusing nature of the labeling in Fig. 3A-C. In Figure 3A, the experimental structure used (PDB 7T47) actually contains both GDP and GMPPCP bound to the same protein, likely due to incomplete nucleotide exchange before crystallization trials. We have updated this figure to show only bound GDP to be consistent with figures 3B-C. We have also updated the label of Fig. 3C to indicate that it is the GDP-bound form that is depicted, although BBO-11818 binds both the GDP- and GTP-bound forms. All DSF (Fig. 3D) and ITC (Fig. 3D) experiments in this figure were performed with GDP-bound RAS proteins. We have updated the figure legend to state this explicitly.

c) In Figure 3D, does DFS typically require this high concentration of inhibitor relative to target to detect effect on melting temperature?

Response: The magnitude of the effect of MRTX1133 binding on T_m will vary as a function of MRTX1133 concentration. 250 µM inhibitor was chosen in order to approach the fully-inhibitor-bound state for all mutants so as to observe the maximum possible impact of

inhibitor on T_m. The amount of inhibitor required to achieve this depends on the K_D value for MRTX1133 binding. The G12C/Y96D mutant has the weakest K_D for MRTX1133, about 25.5 μM; with inhibitor at 250 μM, protein at 7.5 μM, and a K_D of 25.5 μM, 90% of the G12C/Y96D protein should have bound inhibitor. For the other mutants, which all bind MRTX1133 more tightly than G12C/Y96D, the percentage of inhibitor-bound protein under the given experimental conditions is approximately 99%. We have added a sentence to the Materials and Methods to explain the selection of 250 μM MRTX1133 concentration.

Are the differences between control and MRTX1133-bound proteins statistically significant?

Response: For each mutant, we have performed a one-tailed Welch's t-test to determine whether the difference between the mean T_m at 0 μM MRTX1133 and at 250 μM MRTX1133 is statistically significant. For all mutants except G12C/Y96D, the T_m at 0 μM MRTX1133 is statistically significantly lower than the T_m at 250 μM MRTX1133. For G12C/Y96D, the difference just misses statistical significance at a threshold of $\alpha = 0.05$. The details and results of the statistical analysis have been added to Supplementary Figure 2, and the text of the manuscript has been updated accordingly.

d) Figure 4B: Consider including the 2.7Å vs >6 Å for H-bond comparison in the figure itself or in the caption.

Response: We have updated the legend of Figure 4B to explicitly state the 2.7 Å and 6.7 Å distances between residues 96 and 58 in the G12C/Y96D and G12C structures, respectively. We have also labeled the 2.7 Å H-bond distance in Figure 4B.

e) In page 7, "The fully open Switch-I conformation observed in G12C/Y96D has been reported in multiple GDP-bound KRAS structures and is consistently associated with crystallization in space group P321 with similar unit cell parameters, suggesting that this lattice is diagnostic of the open state. As in prior examples…" Please mention "Supplementary Figure 7" in these earlier statements to reference these results.

Response: As requested, we have moved the reference to Supplementary Figure 7 (now Supplementary Figure 8 in the revised manuscript) to a point a few sentences earlier in the text to explicitly link this statement to the structural comparison supporting the association between the fully open Switch-I conformation and the *P*321 crystal lattice.

f) How does the G12C/Y96D effect in GTP-KRAS relate to a reported off-state G12Ci escape/bypass into GTP-bound fraction (PMID: 39215000)?

Response: We thank the reviewer for this question and for highlighting the recent study (PMID: 39215000), which shows that resistance to KRAS G12C (OFF) inhibitors can arise through increased KRAS-GTP loading, rendering the inactive state inaccessible to these drugs. Our findings with the Y96D mutation are consistent with and complementary to this mechanism. Y96D structurally occludes the Switch-II pocket in the GDP-bound state, impairing inhibitor binding, while preserving the competence of the GTP-bound conformation to interact with effectors. Thus, unlike adaptive resistance driven by increased GTP loading, Y96D confers resistance by limiting inhibitor access even in the GDP state while maintaining signaling in the active state. We have added text to the manuscript to clarify this relationship.

g) On page 14, the authors state that Y96D preserves near-native binding to both Raf1 and PI3Kα. This is true for PI3Kα (Figure 6E) but affinity to Raf1 is much higher (>10 x). The Kd for G12C and Raf-RBD is 9.4 μM while that for G12C-Y96D is 0.315 μM. It is also interesting, but a bit confusing, that the mutants (except Q99L) have better binding affinities to Raf1.

Response: We thank the reviewer for this careful observation. As noted in the manuscript, the apparent affinity measured for KRAS G12C–RAF1 RBD by ITC is weaker than values previously reported using SPR and related approaches. In our earlier ITC measurements, the KRAS G12C titration curves did not extend sufficiently into the saturation regime, resulting in a weakly constrained fit with large uncertainty (~9 ± 5 μM). We therefore repeated these experiments using optimized protein concentration ratios, yielding a substantially better-constrained KD of ~2 ± 0.3 μM for KRAS G12C with RAF1 RBD. With this revised value taken into account, the resistance mutants generally show comparable or weaker RAF1 binding relative to KRAS G12C. Thus, the apparent tighter binding of several mutants relative to the initial G12C value primarily reflects uncertainty in the earlier parental measurement rather than a true enhancement of RAF1 engagement. The notable exception is Y96D, which reproducibly exhibited stronger apparent binding by ITC (K_D = 0.315 μM), consistent with our conclusion that it preserves near-native RAF1 engagement. This is further supported by the co-crystal structure of G12C/Y96D-GMPPNP bound to RAF1 RBD-CRD (Fig. 4D), which closely superimposes with the WT KRAS–RAF1 complex (backbone RMSD 0.22 Å).

Importantly, our primary conclusion does not depend on precise absolute K_D values, but rather on the observation that all resistance mutants retain stable RAF1 binding. Orthogonal analytical size-exclusion chromatography experiments (Fig. 6C) independently confirmed complex formation for all resistance variants examined.

Reviewer #4:

This manuscript provides a comprehensive structural and mechanistic analysis of resistance to KRAS G12C Switch-II pocket inhibitors in KRAS G12C. Using a combination of crystallography, NMR, molecular dynamics, biochemical assays, and cell signaling data, the authors define two major resistance mechanisms: (1) loss of key inhibitor interactions (Type I mutants) and (2) conformational rearrangement and increased dynamics of the Switch-II pocket (Type II mutants). These mutations reduce both covalent and noncovalent inhibitor engagement while largely preserving downstream MAPK and AKT signaling. The study offers valuable guidance for next-generation inhibitor design. Major strengths include the integration of structural, biophysical, and cellular data, classification of resistance mechanisms, and meaningful implications for drug design strategies. However, there exist concerns and points for clarification.

1. Mutant selection and scope.

The frequencies of clinically observed secondary mutations should be provided.

Response: We thank the reviewer for this suggestion. Please see our response to Reviewer 1 for a detailed discussion of the currently available evidence regarding the occurrence of these resistance mutations in patients. We have now added a discussion of this topic to the Introduction. Although precise per-mutation frequencies have not yet been established, available clinical data indicate that Y96D is the most frequently observed clinically acquired Switch-II pocket mutation, representing the most common mutation in this class in a 143-patient post-progression cohort in which Switch-II pocket mutations emerged in 14% of patients overall (Riedl et al., 2025, ref. 5). In contrast, R68S, H95Q, Y96C, and Q99L have been reported less frequently, with Q99L initially identified as the dominant secondary mutation in adagrasib-resistance Ba/F3 models before subsequently being observed clinically. We have added the following text to the revised Introduction:

"Several clinically acquired resistance mutations have been identified within the KRAS Switch-II pocket following treatment with KRAS(G12C) inhibitors. Among these, Y96D is the most frequently observed mutation, representing the most common Switch-II pocket substitution in a 143-patient post-progression cohort in which Switch-II pocket mutations emerged in 14% of patients overall (Riedl et al., 2025). Additional recurrent mutations, including R68S, H95Q, Y96C, and Q99L, have also been reported in patients or preclinical resistance models (Awad et al., 2021; Koga et al., 2021)."

Moreover, the mutations studied are biased toward resistance to adagrasib. Hence, inclusion or discussion of sotorasib-specific resistance mutations would strengthen conclusions.

Response: We thank the reviewer for this observation. The mutation panel is enriched for mutations initially identified in adagrasib resistance studies, reflecting the availability of the earliest comprehensive post-progression clinical datasets (Awad et al., 2021; Koga et al., 2021). However, the mutations examined here were selected because they represent the major recurrent Switch-II pocket resistance hotspots identified across clinical and preclinical studies rather than because they are specific to a single inhibitor. In addition, resistance mutations exhibit clear inhibitor-specific effects. Clinical and preclinical studies have shown that R68S and Y96 substitutions impair both adagrasib and sotorasib, whereas H95 substitutions predominantly affect adagrasib but have little effect on sotorasib, consistent with differences in how these inhibitors engage the Switch-II pocket (Awad et al., 2021; Koga et al., 2021; Rubinson et al., 2024). This inhibitor-selective pattern is directly reflected in our k_{inact}/K_I and cellular signaling data. We have expanded the Discussion to place our findings in the context of inhibitor-specific resistance mechanisms and their relevance to the broader class of Switch-II pocket inhibitors. We have added a brief discussion of these points to the revised manuscript:

"Although the resistance mutations examined here were initially identified in adagrasib resistance studies (Awad et al., 2021; Koga et al., 2021), they represent the major recurrent Switch-II pocket hotspots observed across clinical and preclinical resistance datasets. Importantly, these mutations exhibit distinct effects across KRAS(G12C) inhibitor scaffolds. R68S and Y96 substitutions impair both adagrasib and sotorasib, whereas H95 substitutions preferentially affect adagrasib and have comparatively little effect on sotorasib (Riedl et al., 2025; Mehran et al., 2024; Rubinson et al., 2024). Consistent with these observations, our kinetic, structural, and cellular analyses reveal both shared and inhibitor-specific resistance mechanisms, highlighting how differences in Switch-II pocket engagement influence mutational vulnerability and shape the emergence of resistance under therapeutic pressure."

The rationale for testing G12D inhibitors and G12D-associated resistance mutations in a G12C context should be clarified.

Response: We appreciate the reviewer's comment and have clarified our rationale in the revised manuscript. Although MRTX1133 was originally developed as a KRAS-G12D-selective inhibitor, it also binds KRAS-G12C and numerous other oncogenic KRAS mutants with high affinity and is among the best-characterized noncovalent Switch-II pocket inhibitors available. We therefore used MRTX1133 as a tool compound to assess the effects of resistance mutations on noncovalent Switch-II pocket recognition, independent of the covalent chemistry used by KRAS-G12C inhibitors such as adagrasib and sotorasib. Thus, MRTX1133 was included as a mechanistic probe of Switch-II pocket engagement rather than

as a prospective therapeutic agent in the KRAS-G12C setting. We have clarified this rationale in the revised manuscript.

2. Structural analyses/interpretation.

Additional consideration/analyses are needed for potential warhead misalignment vs. affinity-driven resistance. Please state whether structural evidence suggests a "warhead misalignment" in any of the mutants.

Response: We thank the reviewer for this insightful suggestion. Our structural and biochemical data indicate that the resistance mechanisms represented in this mutant panel are not solely explained by reduced noncovalent affinity. Several mutations, particularly R68S, H95Q, Y96C, and Y96D, alter the geometry of the Switch-II pocket and disrupt interactions that help position the inhibitor for productive covalent engagement. Consistent with this interpretation, these mutants often exhibit substantially larger effects on kinact/KI than on noncovalent binding affinity alone. These observations are consistent with a model in which resistance can arise from a combination of weakened pocket recognition and, in some cases, warhead misalignment, which reduces the efficiency of covalent bond formation with KRAS(G12C). We have incorporated these ideas into sections of the results and discussion in the revised manuscript.

The study suggests that "distributing binding interactions" across the pocket can mitigate Type I resistance. Could the authors suggest which specific residues within the Switch-II area are most "mutation-proof" based on their structural data? Identification of “mutation-resistant” residues for drug design may aid in future drug discovery efforts.

Response: We thank the reviewer for this insightful question. Our data do not point to any single residue as being “mutation-proof.” Instead, they suggest that resistance is most likely to emerge when inhibitor binding depends heavily on a few hotspot residues, such as H95, Y96, R68, or Q99. A more robust design strategy may be to distribute binding interactions across multiple pocket residues and conserved backbone atoms, thereby reducing dependence on any single side-chain interaction and improving resilience to resistance mutations.

Type II resistance appears to rely on a non-native D96-T58 H-bond. From MD analysis, does the mutation trap a "structural water" that further prevents drug entry? The discovery of the nucleotide-dependent intramolecular interactions (D96-T58 in GDP-bound vs. D96-R68 in GTP-bound) is intriguing. A comparison of intramolecular interactions (e.g., D96-R68 vs. R68-E37 competition) and their impact on Switch-I stability should be included.

Response: We interpret the reviewer's reference to "structural water" as either a crystallographically observed water or a highly occupied (persistent) water observed during the course of our MD simulations. Upon investigating the crystal structure and the simulations, there are no obvious water molecules that would occlude ligand binding; however, because we are simulating the inhibitor-free states of these mutants, and inhibitor binding causes substantial restructuring of the binding pocket residues, it is difficult to state this with confidence. To address this, we have looked for waters near residues D96 and T58, particularly in the frames where the D96-T58 H-bonding interaction occurs. We observe long-lived water interactions with D96, which occur alongside the continued interaction between D96 and T58.

Regarding the nucleotide dependence of D96 interactions with either T58 or R68, we have analyzed how often these interactions occur in our simulations. The following table summarizing the results has been added as the new Supplementary Table 2, and the main text has been updated accordingly:

Nucleotide	**GDP**		**GTP** (structure isolated from complex with RAF1)	
	% of frames containing interaction		% of frames containing interaction	
Replica	**D96-T58**	**D96-R68**	**D96-T58**	**D96-R68**
1	49.8	0.04	0	64.6
2	89.7	0.7	0	33.7
3	76.3	21.3	0	65.6
4	79.4	0.02	0	84.4
5	33.3	44	0	95.3

Nucleotide	**GDP**		**GTP** (structure isolated from complex with RAF1)	
	% of frames containing interaction		% of frames containing interaction	
Replica	**D96-T58**	**D96-R68**	**D96-T58**	**D96-R68**
1	49.8	0.04	0	64.6
2	89.7	0.7	0	33.7
3	76.3	21.3	0	65.6
4	79.4	0.02	0	84.4
5	33.3	44	0	95.3

Nucleotide	GDP		GTP (structure isolated from complex with RAF1)	
	% of frames containing interaction		% of frames containing interaction	
Replica	D96-T58	D96-R68	D96-T58	D96-R68
1	49.8	0.04	0	64.6
2	89.7	0.7	0	33.7
3	76.3	21.3	0	65.6
4	79.4	0.02	0	84.4
5	33.3	44	0	95.3

Nucleotide	GDP		GTP (structure isolated from complex with RAF1)	
	% of frames containing interaction		% of frames containing interaction	
Replica	D96-T58	D96-R68	D96-T58	D96-R68
1	49.8	0.04	0	64.6
2	89.7	0.7	0	33.7
3	76.3	21.3	0	65.6
4	79.4	0.02	0	84.4
5	33.3	44	0	95.3

As shown in the above table, the D96-T58 interaction is quite stable across all GDP-bound simulations. In contrast, the D96-R68 interaction does exist but is much less prevalent. In the GTP-bound simulations, the D96-T58 interaction is not observed, whereas the D96-R68 interaction has become dominant.

Regarding R68-E37 interactions, none are observed in the simulations of the GDP-bound state. In the active-state Y96D simulations, this interaction occurs in only 24 of 500,000 frames, all of which are from one of the five simulation replicas. From a structural analysis of the conformational states corresponding to this interaction, we observe that the dynamics of Switch I are not significantly perturbed relative to the state in which R68 interacts with D96. Therefore, we consider it unlikely that the R68-E37 interaction plays a major role in either the GDP-bound or GTP-bound states. Rather, it is the addition of an RBD binding partner that enables the stable interaction of R68-E37 in the crystal structure.

The authors observe a unique phenomenon where the H95Q mutation slightly enhances the covalent engagement of sotorasib (kinact/KI increase from 10,800 to 14,400 M-1s-1), whereas it severely impairs adagrasib and BBO-8520. A structural overlay of the sotorasib-bound model with the H95Q mutant structure will aid in visualization of whether this preference is driven by the relief of a specific van der Waals clash or the formation of a new polar network.

Response: We acknowledge the reviewer's observation regarding the effect of H95Q on sotorasib. However, the observed change is modest compared with the pronounced resistance phenotypes seen for other mutants, and in the absence of a structure of sotorasib bound to the H95Q mutant, we prefer not to overinterpret this observation. While subtle changes in local packing or pocket interactions may contribute, we do not currently have sufficient structural evidence to distinguish among potential mechanisms. We have

therefore limited our discussion to noting that sotorasib is largely insensitive to H95 substitutions, consistent with its distinct mode of engagement within the Switch-II pocket.

3. ITC data.

The affinity for RAF RBD with KRAS G12C appears low compared to previous reports (e.g. Alexander et al, JBC (2025) by the RAS NCI group). It would be helpful to compare ITC-derived affinities (e.g., KRAS–RAF) with the literature for consistency and whether the constructs differ.

Response: As also noted in our responses to Reviewers 1 and 2, the apparent affinity measured for KRAS G12C–RAF1 RBD by our initial ITC experiments was weaker than values previously reported using SPR and related approaches, including recent measurements from the NCI RAS Initiative (Alexander et al., J Biol Chem 2025), which generally place KRAS–RAF1 RBD interactions in the sub-micromolar range. Consistent with this, our recent study by Bonsor et al. (Nat Commun 2026) measured WT KRAS–RAF1 RBD binding by optimized ITC at ~200 nM. The same study also reported ITC-derived affinities of ~5 nM and ~32 nM for MRTX1133 binding to WT and G12D KRAS-GDP, respectively, demonstrating that high-affinity KRAS interactions can be robustly measured by ITC under optimized conditions.

In our earlier KRAS G12C–RAF1 RBD measurements, the titration curves did not extend sufficiently into the saturation regime, resulting in a weakly constrained fit with relatively large uncertainty (~9 ± 5 μM). We therefore repeated the experiments using optimized protein concentration ratios that produced improved binding isotherms and substantially better-constrained fits, yielding a KD of ~2 ± 0.3 μM for KRAS G12C with RAF1-RBD. This revised value is considerably more aligned with the broader literature.

The heat of dilution is not subtracted in many Wiseman plots, with some datasets appearing truncated or poorly fit.

Response: We wish to emphasize that no ITC dataset anywhere in the manuscript has been truncated. Instead, the two measurements for each pair of binding partners were often performed at different ratios of the binding partner concentrations in the ITC sample cell and injection syringe, an approach that is mathematically superior to simply repeating an experiment multiple times at the same concentration ratio. As a result of the varied concentration ratios, the two experiments for a given pair of binding partners cover different ranges of the x-axis variable (Molar Ratio). To make this explicit, we have reformatted Supplementary Figures 8 and 10 (now Supplementary Figures 9 and 11 in the revised manuscript) to show each individual dataset and have labeled them with the specific concentrations used in each experiment.

Furthermore, no separate experiments to measure and then subtract heats of dilution were performed; instead, the heat of dilution is accounted for by an “offset” term that is fit as part of the data-fitting process. The Wiseman plots displayed in the manuscript result from the integration of the raw data without any truncation or other data manipulation.

A subset of the figures provides replicates (Fig 3, 6) while others do not (Fig 2, 7).

Response: The replicate measurements for Fig. 2 are shown on the bar plots in panels D, E, and F. Regarding the cell-based assays of MAPK pathway suppression in Figure 7, we have now performed two additional biological replicates and provided statistical details of the results.

In Fig 1 I, the labeling of G12C-GDP/GTP is misleading, considering the structure shown is of GDP.

Response: We apologize for the confusion. The figure was labeled “GDP/GTP” to indicate that BBO-8520 is a dual-state inhibitor. As the reviewer indicated, however, the specific structure shown contains only GDP. We have updated Figure 1I and the Figure 1 legend to reflect this.

In Fig 3E , the heat of dilution for the 1st one is subtracted, while the other 5 are not.

Response: All thermograms were analyzed using a fitting procedure that includes an offset term to account for residual heats of dilution and baseline differences between experiments. The apparent difference in the first dataset therefore reflects the fitted offset parameter rather than differential processing of the data. No manual subtraction of the heat of dilution was performed for any of the ITC datasets. All datasets were processed and analyzed in an identical manner.

In SI Fig 3, only the 1st one is correct, which is the same one shown in Fig 3E.

Response: As discussed earlier, no separate experiments to measure and subtract the heat of dilution were performed. The plotted data come directly from the integrated raw data.

For Fig 6 B and E, should the Wiseman plots start at the same point when the plateau doesn’t go to zero (heat of dilution not subtracted in select plots, specifically E).

Response: The ITC experiments shown in Fig. 6B and 6E were performed at different protein concentrations and with varying injection volumes and numbers of injections to obtain reliable replicate datasets across the measured affinity range. Consequently, the Wiseman

plots are not expected to begin at the same absolute value. Importantly, the reported K_D values were obtained by global fitting across replicate experiments, rather than from individual isotherms, thereby providing a more robust estimate of the binding affinity. Therefore, the apparent differences in the starting points or plateaus of the individual Wiseman plots do not affect the reported binding parameters.

In SI Fig 8, the grey replicate for the G12C-RAF1 is truncated and linear, neither fit shows a plateau.

Response: As discussed previously, no ITC plots in this manuscript are truncated. To alleviate the concern about the lack of plateau in the ITC data for the G12C-RAF1 RBD pair, we have performed two new measurements of the binding of this interaction pair at syringe and cell concentrations that sample much higher molar ratios on the x-axis; the plateau is now obvious. The fit K_D value is 2 μM, which is within the uncertainty of the previously measured value of 10 μM. The confidence interval of the new value of 2 μM is now very narrow (1.6 – 2.7 μM), reflecting the high quality of the data. Figure 6B and Supplementary Figure 8 (now Supplementary Figure 9 in the revised manuscript) have been updated with the new data.

In SI Fig 10, several replicates do not have heat of dilution subtracted. dH values are not trustworthy when heats of dilution have not been subtracted.

Response: We emphasize that we have made no arguments in this manuscript based on fitted ΔH values.

The biphasic binding behavior (e.g., Q99L) may indicate aggregation or multiple binding modes and should be investigated.

Response: At present, we have no data that enable us to interpret the biphasic binding curve for Q99L observed in Figure 6B. Our chief concern was whether Q99L is still capable of binding downstream signaling partners, which is a necessary capability for the Q99L mutation to successfully impart resistance. This is why we undertook the analytical size exclusion experiments (Fig. 6C) as an orthogonal method to verify binding. From the elution chromatograms, we conclude that the affinity of Q99L for the RAF1 RBD cannot be substantially weaker than the other resistance mutants, as all samples were mixed at the same concentration ratio and there is no evidence of residual unbound RBD in the Q99L chromatogram. Neither is there any sign of high-molecular-weight aggregates in the elution profile.

4. Methodology.

Methodological details are lacking for the DSF fluorophore, statistical reporting, and replicate consistency.

Response: The identity and concentration of the DSF fluorophore have been added to the Materials and Methods section.

In Figure 3D, all bars and error bars represent the mean and standard deviation, respectively, of three technical replicate measurements. This information is given in the figure legend. In many cases, the magnitude of the standard deviation is small enough that the error bars are not readily apparent on the graph; we have updated the figure legend text to state this explicitly.

MD simulation convergence (e.g., RMSD) should be shown to support conclusions. The authors performed 5 x 1 μs simulations for all variants. More details regarding the convergence of these simulations (e.g., RMSD plots) to ensure the observed populations of "occluded" states are statistically robust are needed.

Response: To address the reviewer's comment, we now provide a new Supplementary Figure 13, which contains the RMSD plots for each of five replica simulations of the reference structure (G12C-GDP), the G12C/R68S-GDP mutant, the G12C/H95Q-GDP mutant, the G12C/Q99L-GDP mutant, the G12C/Y96C-GDP mutant, the G12C/Y96D-GDP mutant, and the G12C/Y96D-GTP structure extracted from the complex with RAF1-RBD-CRD.

As shown in the RMSD plots, RMSD values are generally stable between 1 and 3 Å across all simulations, with some isolated excursions past 3 Å, especially in certain replicas of the G12C/Y96D-GDP and G12C/R68S-GDP mutant simulations, the two mutations that are the most deleterious with respect to inhibitor efficacy. The G12C/Y96D-GDP simulations exhibit the largest conformational variability among all simulations, likely due to the presence of the fully open Switch-I conformation. Overall, the RMSD results demonstrate that our systems are sampling the equilibrium ensemble and show that the most dynamic mutants have a greater impact on inhibitor efficacy.

5. NMR data.

HSQC data suggest possible conformational heterogeneity in certain mutants. Some figure labeling and presentation require correction for clarity. For example, the HSQC spectrum for G12C/Y96D-GDP shows a potential 2nd conformation from the population of weak peaks.

Response: We have updated Supplementary Figure 6 (now Supplementary Figure 7 in the revised manuscript) with assignment labels for some of the identifiable crosspeaks that

exhibit a minor secondary conformation. The identified peaks are not found on Switch-I, which adopts the fully open conformation in the Y96D(GDP) structure.

6. Cell signaling.

Error quantification is lacking for the signaling assays, making it difficult to interpret the data presented.

Response: To address this concern, we have expanded the analysis of signaling assays to include three independent biological replicates for each condition. We also performed statistical analyses of the resulting data. Importantly, the overall conclusions remain unchanged, with all replicates showing the same rank order of inhibitor sensitivity/resistance across the mutant panel. The revised figure and accompanying statistical analyses have now been incorporated into the revised manuscript.

While RAS resistance mutants appear to maintain MAPK signaling in the presence of RAS inhibitors, AKT effects are weaker and require further discussion. It is unclear whether limited pAKT suppression may reflect pathway redundancy or cell-line-specific biology rather than KRAS effects alone.

Response: The most pronounced and consistent signaling effects observed in this study were on the MAPK pathway, whereas effects on pAKT were more modest and variable. Moreover, even in KRAS G12C-expressing cells, the dual GDP/GTP-state inhibitor BBO-8520 produced only modest pAKT suppression, while the GDP-state-selective inhibitors sotorasib and adagrasib had even smaller effects. We therefore interpret the pAKT results as reflecting both the partial dependence of AKT signaling on KRAS signaling and the contribution of additional upstream inputs and pathway redundancy in this cellular context. Accordingly, we have focused our mechanistic conclusions on the more robust and direct effects observed for MAPK signaling. We have described these observations regarding pAKT signaling in both the Results and Discussion sections.

It is also unclear whether the RAS resistance mutants studied alter signaling in the absence of inhibitors.

Response: With respect to signaling in the absence of inhibitor, the resistance mutants did not produce notable differences in basal pathway activity in our signaling assays. Rather than substantially altering signaling output under uninhibited conditions, their primary effect was to preserve downstream signaling in the presence of inhibitor, consistent with a role in mediating resistance rather than broadly rewiring signaling.

In summary, this study represents a strong and impactful study that advances understanding of KRAS inhibitor resistance. However, issues related to data analysis, methodological details, and mechanistic interpretation should be addressed to strengthen confidence in the conclusions.

Response: We thank the reviewer for the constructive feedback and positive assessment of the study. In response to the reviewer's comments, we have further strengthened the manuscript by conducting additional ITC measurements, including three biological replicates of the cell signaling assays, performing statistical analyses for the signaling assays, clarifying experimental and analytical procedures, expanding the discussion of the clinical relevance of the resistance mutations, and refining several mechanistic interpretations. We believe these revisions improve the rigor of the study and reinforce the conclusions presented.

Point-by-point response to reviewer's comments:

We appreciate the reviewers' thorough and constructive feedback. In this revised manuscript, we have addressed all concerns raised. Below, we provide point-by-point responses to the reviewers' comments (in blue).

Reviewer 3

In the Introduction (pages 2-4) and elsewhere, it would help to be consistent if the justification for the choice of resistance mutations (especially the non-Y96D) was based on "most recurrent" (i.e., occurring in different studies) or "most common" (which denotes mutational prevalence). The two terms are different but appeared to be used interchangeably.

Response: We thank the reviewer for this helpful suggestion. Throughout the revised manuscript, we now consistently describe the mutations selected for this study as the most recurrent clinically acquired Switch-II pocket resistance mutations, while reserving most frequently observed specifically for Y96D, for which clinical data support its higher prevalence.

The rebuttal states that "Structural studies additionally show that the MRTX1133 binding pose is highly conserved across KRAS alleles..." This important justification should be included in the Results.

Response: We thank the reviewer for this suggestion and agree that this rationale belongs in the manuscript. We have added a brief explanation to the Results noting that, although MRTX1133 was developed for KRAS(G12D), its binding pose is highly conserved across KRAS alleles and closely resembles the pre-covalent binding mode of clinical KRAS(G12C) inhibitors. We also note that MRTX1133 is a well-characterized noncovalent probe for assessing how resistance mutations affect Switch-II pocket recognition.

Clarify whether disruption of the Switch-II pocket is "a major" or "the major" contributor to resistance.

Response: We agree that our data support the disruption of Switch-II pocket engagement as a major contributor to resistance, rather than as the exclusive mechanism. We have revised the manuscript throughout to use this more accurate wording.

Do the authors believe the Type I/Type II classification might be applicable to other prevalent G12X mutants?

Response: We thank the reviewer for this insightful question. It is likely that the Type I/Type II classification also applies to other KRAS G12X mutants, provided the inhibitors bind the

Switch-II pocket. In that context, resistance mutations would likely act through the two general mechanisms described here: disruption of inhibitor-pocket interactions (Type I) or stabilization of pocket-occluding conformations (Type II). We have added a sentence to the Discussion acknowledging this possibility while noting that additional studies will be needed to establish its broader applicability.

Reviewer 4

Figure 3A remains labeled as GMPPCP.

Response: We thank the reviewer for noticing the discrepancy in Fig. 3A. To remove any ambiguity, we have recreated the panel using a different crystal structure (PDB 7RPZ) of KRAS-G12D in complex with MRTX-1133; this structure contains only GDP-bound KRAS. Additionally, we have updated the manuscript text and figure legend to indicate the PDB code of the replacement structure.

The authors propose that the Y96D mutation adopts a fully open Switch-I conformation that is independent of crystal lattice contacts, stating that "Widespread chemical shift perturbations, including residues in Switch-I and Switch-II, indicate that the observed conformational rearrangements are not solely crystal lattice-induced…". However, the NMR data presented in Supplementary Figure 7 do not appear to fully support this conclusion. Only modest chemical shift perturbations are observed for residues within the Switch-I region, whereas more pronounced effects, including evidence of multiple conformational populations, are primarily observed for residues proximal to the nucleotide-binding motifs. The authors should either provide additional evidence supporting extensive Switch-I rearrangement in solution or temper their interpretation to better reflect the experimental data.

Response: We thank the reviewer for this thoughtful observation. We agree that our original wording overstated the conclusions that can be drawn from the NMR data. While the observed chemical shift perturbations support the conclusion that the Y96D mutation induces conformational changes in solution that are not solely attributable to crystal lattice contacts, the largest perturbations and evidence of multiple conformational populations are observed primarily near the nucleotide-binding region and Switch-II, with more modest effects in Switch-I. We have therefore revised the text to better reflect the experimental data by tempering our interpretation of the solution-state conformation while maintaining the conclusion that the Y96D mutation induces conformational changes beyond those imposed by crystal packing.
